# Optimal design of dose and drug pharmacokinetic characteristics to achieve the desired pharmacodynamic profile in repeated drug dosing

**DOI:** 10.1371/journal.pone.0354029

**Published:** 2026-07-22

**Authors:** Martin Dodek, Zuzana Vitková, Anton Vitko, Eva Miklovičová

**Affiliations:** Institute of Robotics and Cybernetics, Faculty of Electrical Engineering and Information Technology Slovak University of Technology in Bratislava, Ilkovičova, Bratislava, Slovakia; University of Milano Bicocca, ITALY

## Abstract

Oral drug therapy requires achieving a delicate balance between therapeutic efficacy and patient safety, yet current dosing strategies often rely on empirical trial-and-error methods that overlook the complex nonlinear dynamic nature of drug behavior in the human body. Conventional pharmacokinetic/pharmacodynamic (PK–PD) approaches provide valuable insights but lack a systematic method for designing dose sequences and formulations that achieve an optimal therapeutic response. This work introduces a structured optimization framework that combines PK–PD modeling, impulsive dosing concepts, and nonlinear optimization to determine optimal repeated oral dosing regimens. We model each orally administered dose as an impulsive input in a linear compartmental PK system and couple the resulting drug concentration profile with a nonlinear Hill-type PD model. To enable efficient optimization, we derive sensitivity functions describing how the therapeutic effect depends on dose size and adjustable drug-formulation parameters, allowing to construct the Jacobian required by the Gauss–Newton nonlinear least-squares algorithm. The proposed method jointly optimizes dose magnitude and formulation-dependent liberation (release) rate to match a clinically meaningful therapeutic effect trajectory. Using a four-compartment pharmacokinetic model, we demonstrate in silico that the method achieves rapid onset, stable long-term therapeutic effect, and reduced fluctuations of the therapeutic effect across repeated dosing cycles.

## Introduction

Executing effective oral drug therapy for various diseases lies at the heart of modern medicine, yet achieving the right balance between efficacy and safety remains a persistent challenge. Pharmacokinetics (PK)—the study of how a drug is absorbed, distributed, and eliminated by the body—traditionally provides a good quantitative framework to understand drug behavior over time. Complementing this, pharmacodynamics (PD) describes how drugs exert their therapeutic effects through receptors. Despite decades of research, translating PK–PD knowledge into precise, truly optimal dosing regimens remains limited [1].

Currently, standard PK–PD research relies heavily on in vitro and in vivo experiments. Although these experiments generate valuable scientific data, their results are often incorporated into clinical practice through rather simplistic (empirical) dosing guidance strategies and protocols. These strategies rely on a pure trial-and-error approach, or simplistic models and assumptions, leading to suboptimal therapeutic outcomes [2].[2] While standard PK–PD methodologies, which assume parameters like bioavailability, clearance, AUC, Cmax, Tmax, still remain relevant in drug development and clinical pharmacology due to their interpretability, they have inherent limitations in achieving optimal therapeutic control [2,3]. A major limitation of current drug treatments is the lack of variable-size dosing, where an initial leading dose is followed by a sequence of maintenance doses [4,5]. Another potential for improvement lies in the dosing with variable release (liberation) rate per dose, which is critical for drugs requiring rapid onset of action while minimizing toxicity and side effects.

Classical PK–PD methodologies for dose optimization have been widely applied in antimicrobial therapy and drug development. A large body of work, summarized in [6], emphasizes the identification of key PK–PD indices such as the ratio of area under the concentration curve to minimum inhibitory concentration (AUC/MIC), peak concentration to MIC (Cmax/MIC), or the duration of time that drug concentration remains above the MIC (T > MIC). These indices are typically determined through dose-fractionation studies and subsequently used as targets for regimen design, with dosing strategies selected to maximize the probability of target attainment in simulated patient populations. This framework has proven highly valuable in clinical pharmacology because it provides a simple and robust way to link drug exposure with antimicrobial efficacy while accounting for interpatient variability through Monte Carlo simulations. However, the PK–PD index paradigm inherently reduces the complex pharmacokinetic and pharmacodynamic dynamics to a small number of scalar exposure metrics and therefore provides limited ability to shape the therapeutic effect. In particular, such approaches typically assume fixed dosing structures and fixed formulation characteristics, and do not explicitly address transient treatment behavior such as the speed of therapeutic onset, suppression of oscillations between repeated doses, or avoidance of pharmacodynamic overshoot. In contrast, the present work formulates repeated oral dosing as a structured PK–PD optimization problem in which repeated oral dosing is modeled as impulsive inputs to a compartmental pharmacokinetic system coupled with a nonlinear pharmacodynamic response. Rather than targeting exposure thresholds, the proposed framework directly optimizes the time-domain therapeutic effect trajectory.

In medicine, most therapeutics—drugs, hormones, and biochemical regulators—are delivered in discrete intermittent doses rather than through continuously modulated infusion. These doses are well represented mathematically as impulsive inputs. Therefore, impulsive control has been successfully used to model and optimize dosing strategies across anticancer chemotherapy [7–10], pharmacokinetic compartmental models [5,11,12], as well as in glycemia control via insulin administration in diabetic patients [13–16].

Impulsive systems form a distinct subclass of hybrid dynamical systems in which the control inputs act through instantaneous, sparsely occurring impulses rather than through continuously applied signals. In such systems, the input is nonzero only over very short time intervals and typically appears at isolated time instants [17]. A defining feature is that at least one component of the state undergoes abrupt discontinuities—state “jumps”—whenever an impulse is applied [17]. Consequently, their trajectories are piecewise continuous and exhibit first-kind discontinuities [13].

From a systems-theoretic viewpoint, impulsive dynamics impose structural constraints that differ markedly from classical continuous-time control. In particular, linear impulsive systems cannot, in general, be stabilized toward an arbitrary equilibrium point by impulsive inputs alone [18]. This leads to inherently pseudo-periodic or oscillatory behavior in closed-loop operation

Optimal drug dosing has been extensively studied using control-theoretic, pharmacokinetic–pharmacodynamic, and impulsive system frameworks. Existing approaches differ in how therapeutic objectives are defined, how dosing actions are modeled, and whether formulation-dependent pharmacokinetic properties are explicitly considered.

A large class of methods formulates dosing optimization primarily at the pharmacokinetic level, with the aim of maintaining drug concentrations within target ranges. Continuous-time optimal control of linear compartmental PK models has been studied using infusion-based formulations and quadratic concentration tracking objectives [19]. Impulsive variants extend these ideas to discrete dosing and individualized therapy [20,21], while data-driven and mixed-integer formulations address pill-size constraints and population-level safety [22]. Although effective for exposure regulation, these approaches do not explicitly optimize pharmacodynamic response and typically treat drug formulation as fixed.

Recent work [23] introduced the OptiDose framework, which determines optimal dosing schedules for general PK–PD models via classical adjoint-based optimal control. While highly general, these approaches limit analytical insight into how dosing and formulation parameters jointly influence the therapeutic trajectory. In contrast, this work focuses on repeated oral drug administration with linear compartmental pharmacokinetics and static Hill-type pharmacodynamics. This structure allows repeated doses to be represented via superposition and enables analytical derivation of state–parameter sensitivities, providing direct interpretability and the ability to jointly optimize both dose magnitude and formulation-dependent release kinetics.

By treating liberation rate as an optimization variable alongside dose magnitude, the proposed framework simultaneously optimizes dosing and effective pharmacokinetic release characteristics, allowing the emergence of clinically meaningful strategies such as fast-acting loading doses followed by slower maintenance dosing.

Several works incorporate PK–PD coupling through nonlinear pharmacodynamic models, often using Hill-type functions. Threshold-based or constraint-driven formulations have been proposed for chemotherapy and antiviral therapies under impulsive dosing [24,25]. Multi-objective approaches further explore trade-offs between efficacy and toxicity using evolutionary algorithms [26]. Although these studies highlight the benefits of non-uniform dosing, they rely on heuristic optimization, surrogate toxicity metrics, or fixed pharmacokinetic parameters and do not explicitly shape transient therapeutic effect trajectories.

Recent developments emphasize effect-oriented control, particularly in anesthesia and antiviral therapy. Nonlinear optimal control frameworks have been proposed to match desired effect-site concentrations or clinical endpoints using continuous infusion and pre-characterized interaction models [27,28]. However, these approaches assume infusion-based delivery, fixed dosing schedules, or perfect model knowledge, limiting their applicability to repeated oral dosing and formulation design.

Advances in model-informed dose optimization have emphasized statistical and pharmacometric approaches that rely on population simulations and exposure matching. In particular, [29] proposed a framework in which candidate dosing regimens are evaluated by comparing the distribution of simulated pharmacokinetic exposure metrics, such as concentration or AUC, to a reference distribution obtained from a clinically validated population. This approach provides a practical and statistically robust solution when pharmacodynamic targets are unknown or poorly characterized. However, distribution-based exposure matching primarily operates on summary pharmacokinetic metrics and does not explicitly account for the temporal dynamics of therapeutic effect. Consequently, it provides limited ability to shape transient treatment behavior, such as rapid onset of therapy, suppression of oscillations between doses, or control of pharmacodynamic overshoot. In contrast, the present work formulates dose optimization as a dynamical systems problem. Rather than matching exposure distributions, the proposed framework directly optimizes the time-domain therapeutic effect trajectory. While the proposed approach requires an explicitly specified pharmacodynamic target profile and relies on accurate PK–PD model identification, it provides a complementary perspective to exposure-matching strategies by enabling direct design of dynamic therapeutic response.

Impulsive control theory has also been applied to drug dosing, with an emphasis on stability, robustness, and feedback regulation. Threshold-based and hybrid feedback strategies provide strong analytical guarantees and closed-loop convergence to periodic dosing regimes [30,31]. However, these methods are inherently conservative and typically converge to uniform dosing patterns.

Finally, disease-driven impulsive optimization frameworks integrate dosing with tumor, viral, or immune dynamics [32–34]. While these models offer valuable insight into long-term disease control, they often abstract pharmacokinetics or treat drug exposure implicitly, limiting their ability to optimize formulation-dependent drug properties.

In contrast to the above, the present work proposes a PK–PD optimization methodology for repeated oral dosing that (i) formulates the therapeutic objective directly at the pharmacodynamic effect level, (ii) models dosing as impulsive inputs in a general linear compartmental PK system, (iii) jointly optimizes per-dose drug amount and formulation-dependent liberation rate, and (iv) exploits sensitivity-based Jacobians to efficiently solve the resulting nonlinear least-squares problem via the Gauss–Newton iterations. This enables systematic design of non-uniform dosing regimens, revealing loading maintenance strategies with fast therapeutic onset and reduced effect fluctuations as an emergent optimal solution rather than a prescribed heuristic.

Despite its generality within the class of compartmental PK–PD models, the present framework relies on several structural assumptions that define its current scope of applicability. Specifically, the methodology assumes:

• linear compartmental pharmacokinetics,
• static concentration–effect pharmacodynamics represented by a Hill-type function,
• fixed equidistant dosing intervals,
• impulsive approximation of dose administration.

These assumptions were intentionally adopted to obtain a mathematically tractable optimization framework permitting analytical sensitivity derivation, structured Jacobian computation, and efficient Gauss–Newton optimization. Consequently, the present work should be interpreted as establishing a methodological foundation for optimization of repeated oral dosing rather than as a universally general PK–PD framework applicable to all drug classes and physiological conditions.

The most significant contributions of this work can be stated as follows:

• Development of a methodology for joint optimization of dose magnitude and formulation-dependent liberation rate in repeated oral dosing.
• Variable drug size and liberation rate for the leading dose and the maintenance doses to achieve a fast therapeutic onset and a steady long-term response with small fluctuations in therapeutic effect.
• Formulation of a methodology applicable to a broad class of linear compartmental state-space pharmacokinetic models.
• Efficient numerical solution of the optimization problem in terms of nonlinear least squares and the Gauss-Newton algorithm. Utilization of the sensitivity functions with respect to the optimized parameters to find the Jacobian of the optimization problem required for the Gauss-Newton algorithm.

## Materials and methods

### Pharmacokinetic model

One of the central methodological pillars is the use of compartmental PK models, which provide a mathematically rigorous yet physiologically interpretable framework to describe the dynamics of drugs in the body [2,35]. This methodology provides a structured and systematic framework for modeling the dynamic distribution of substances within an organism, encompassing absorption, distribution, metabolism, and excretion processes (ADME) [35]. At its core, compartmental analysis conceptualizes the organism as a system of discrete compartments, each representing a distinct physiological, biochemical, or anatomical region with relatively uniform properties [36]. To quantitatively describe these processes, ordinary differential equations are used. These equations capture the rate of exchange between compartments, which is typically dictated by concentration gradients and transport kinetics [2,3,37].

For the purposes of optimal treatment design, compartmental PK modeling offers several advantages. It enables a clear distinction between pharmacokinetic parameters that can be modified through drug formulation (design)—such as the rate at which the drug is released—and parameters that reflect intrinsic biological properties of the patient, such as metabolic clearance (elimination) or absorption, which cannot be altered by pharmaceutical design. Compartmental PK models are also mathematically well-structured and interpretable through the system and control sciences.

Consider an n-compartment PK model which jointly describes the drug liberation, absorption, transport, and elimination mechanisms through the dynamics of n state variables (drug amounts) using linear differential equations and p model parameters $\theta^{i}\in R^{p}$ corresponding to the PK response to the i-th dose such that


$$\begin{array}{c} {\dot{x}}^{i}(t,\theta^{i})=A(\theta^{i})x^{i}\left(t,\theta^{i}\right)+B(\theta^{i})u^{i}(t) \end{array}$$ (1)

where $A(\theta^{i})\in R^{n\times n}$ [1/h] is a negative-definite Metzler matrix describing the causal relation between the states (compartments), $B(\theta^{i})\in R^{n}$ [mg] is the input gain vector (including the dose size), $u^{i}(t)\in R$ [1/h] is the input representing the unit rate of the drug administration via the oral route, $x^{i}\left(t,\theta^{i}\right)\in R^{n}$ [mg] is the state vector describing the drug amounts in individual compartments (gastrointestinal tract, blood circulation, peripheral blood) after the administration of the i-th dose. The parameter vector $\theta^{i}$ of p adjustable parameters to be optimized includes the dose size and chosen drug characteristics, which can be technologically influenced/modified. The parameters $\theta^{i}$ remain constant between the consecutive dosing injections (discrete events). The other PK parameters affecting $A\left(\theta^{i}\right)$ and $B(\theta^{i})$, such as the elimination rates and inter-compartmental transport remain fixed and immutable.

The dose size $D^{i}\in R$ [mg], as part of the parameter vector $\theta^{i}$, will be incorporated into the input gain vector $B(\theta^{i})$ rather than directly into the input signal $u^{i}(t)$, such that the PK response is linear with respect to $D^{i}$ via $B(\theta^{i})$. This convention allows us to jointly optimize the dose along with the other drug technological parameters (liberation rate) affecting the dynamics via $A\left(\theta^{i}\right)$.

The assumption of linear pharmacokinetics constitutes an important modeling restriction of the present framework. The methodology is therefore primarily applicable to drugs operating within concentration ranges where absorption, distribution, and elimination processes remain approximately first-order and non-saturating.

This excludes drugs exhibiting strong nonlinearities such as saturable metabolism, concentration-dependent clearance, transporter saturation, autoinduction, target-mediated drug disposition, or nonlinear protein binding. Nevertheless, linear compartmental models remain highly relevant in practical pharmacokinetics because many therapeutics operate predominantly within approximately linear exposure ranges under standard dosing conditions.

Considering the repeated dosing with the dosing period $T_{D}$ [h], we will model the administration rate (input) $u^{i}(t)$ [1/h] corresponding to the i-th dose in terms of Dirac function as


$$\begin{array}{c} u^{i}(t)=\delta \left(t-T_{D}(i-\text{1})\right) \end{array}$$ (2)

The Dirac impulse defined in Eq (2) provides a convenient abstraction for oral drug dosing, representing discrete, intermittent administration. In the present study, these doses are assumed to occur at fixed equidistant intervals. This assumption is necessary for mathematical tractability: it ensures that the response to each individual dose can be represented as a vector of known length and starting time, which allows stacking and superposition of the response vectors required for the nonlinear least squares problem. While exact adherence to dosing times may vary, small deviations from prescribed times generally have a limited effect on therapeutic outcomes for drugs with moderate half-lives and gradual pharmacodynamic [2,3]. Allowing fully variable dosing times would transform the problem into a more general hybrid optimal control problem with variable event timing, substantially increasing computational complexity and altering the structure of the sensitivity equations.

Each administration introduces the prescribed dose into the gastrointestinal compartment over a small time interval $\Delta t_{\text{dose}}$that is negligible compared to the characteristic pharmacokinetic time scales, i.e., $\Delta t_{\text{dose}}\ll {\text{min}}_{i}\text{1}/k_{i}$, where $k_{i}$ are the first-order elimination or transfer rates of the compartments in the PK model. This ensures that each dose can be accurately approximated as an instantaneous (impulsive) input for the purpose of PK–PD modeling and optimization. From a physiological standpoint, this ensures that the drug is effectively “available” in the gastrointestinal compartment before significant redistribution or elimination occurs. Consequently, the framework is primarily intended for intermittent oral or bolus-like administration and is not directly applicable to prolonged infusion therapies or continuously varying administration profiles.

All modifiable parameters of $n_{D}$ dosing events can be stacked into the joined parameter vector $\theta \in R^{pn_{D}}$ as


$$\begin{array}{c} \theta =(\begin{array}{c} \theta^{\text{1}}\\ \theta^{\text{2}}\\ \theta^{\text{3}}\\ \vdots \\ \theta^{n_{D}} \end{array}) \end{array}$$ (3)

The overall effect of multiple dose administration can be expressed in terms of the superposition principle applied to Eq (1) as


$$\begin{array}{c} \dot{x}\left(t,\theta \right)=\sum_{i=\text{1}}^{q}\left[A(\theta^{i})x^{i}\left(t,\theta^{i}\right)+B(\theta^{i})u^{i}(t)\right] \end{array}$$ (4)

where q is the number of administered doses up to time t.

The solution of the system state for no previous treatment, represented by x(0,θ)=0, then gets [38,39]


$$\begin{array}{c} x\left(t,\theta \right)=\sum_{i=\text{1}}^{q}x^{i}\left(t,\theta^{i}\right)=\sum_{i=\text{1}}^{q}e^{A(\theta^{i})\left(t-(i-\text{1})T_{D}\right)}B(\theta^{i}) \end{array}$$ (5)

The partial contribution of the i-th dose $x^{i}\left(t,\theta^{i}\right)$ gets


$$\begin{array}{c} x^{i}\left(t,\theta^{i}\right)=e^{A(\theta^{i})\left(t-(i-\text{1})T_{D}\right)}B(\theta^{i}) \end{array}$$ (6)

The drug concentration c(t,θ) [mg/ml] in the drug receptor (target) compartment is related to the drug amount x(t,θ) such as


$$\begin{array}{c} \left(t,\theta \right)=C^{\text{T}}x(t,\theta ) \end{array}$$ (7)

where the structure of vector $C\in R^{n}$ depends on which compartment (state variable) contains the drug receptors and on the corresponding distribution volume V [ml] such that


$$\begin{array}{c} C=(\begin{array}{c} \text{0}\\ \vdots \\ \frac{\text{1}}{V}\\ \vdots \\ \text{0} \end{array}) \end{array}$$ (8)

### Pharmacodynamic model and therapeutic effect profile

While pharmacokinetics describes how a drug is absorbed, distributed, metabolized, and eliminated, it does not by itself determine the therapeutic outcome. Clinical efficacy depends on how drug concentrations interact with molecular targets and translate into biological effects, as described by pharmacodynamics. As a result, PK-only models are insufficient: dosing regimens with similar concentration–time profiles can produce markedly different therapeutic responses when the concentration–effect relationship is nonlinear or saturable. Incorporating pharmacodynamics is therefore essential to link drug exposure to clinical effect and ensure that optimized dosing strategies are pharmacologically meaningful [2,3,35].

One of the most widely used mathematical representations is the Hill function, which describes the relative nonlinear relation between the drug concentration c(t,θ) and its therapeutic effect E(c(t,θ)) in the interval ⟨0,1⟩ as [2,3]:


$$\begin{array}{c} E\left(c\left(t,\theta \right)\right)=\frac{{c(t,\theta )}^{h}}{K^{h}+{c(t,\theta )}^{h}} \end{array}$$ (9)

where K [mg/ml] is the half-saturation constant (the value of c(t,θ) at which E(c(t,θ))=0.5) and h is the Hill coefficient. The parameters K, h represent intrinsic biochemical characteristics of the drug–receptor interaction and therefore cannot be modified through dose design or therapeutic scheduling. The half-saturation constant K quantifies the affinity between the drug molecule and its target receptor, reflecting the equilibrium between the binding and unbinding events at the molecular level. This affinity is determined exclusively by the drug’s chemical structure and the receptor’s binding pocket. Similarly, the Hill coefficient h captures the cooperativity of binding, which depends on structural features of the receptor complex, the conformational changes induced by ligand binding, and the molecular arrangement of binding sites. These properties are fixed for a given drug-target pair and are not affected by dosage form, administration rate, or formulation-based modifications.

In the context of repeated drug dosing, the Hill function becomes particularly powerful: it transforms the concentration–time profile into an effect trajectory that can be shaped through dose size and modifications to pharmacokinetic parameters (PK parameters).

Consequently, the framework does not presently capture dynamic pharmacodynamic phenomena such as receptor turnover, delayed signal transduction, tolerance development, hysteresis, indirect response mechanisms, or adaptive biological feedback. The static formulation was selected intentionally to isolate the influence of pharmacokinetic shaping on the therapeutic effect trajectory while preserving analytical differentiability of the optimization problem.

The derivative of the drug effect E(c(t,θ)) given by Eq (9) (Hill function) with respect to the concentration c(t,θ) gets


$$\begin{array}{c} \frac{\partial E\left(c\left(t,\theta \right)\right)}{\partial c(t,\theta )}=\frac{hK^{h}{c(t,\theta )}^{h-\text{1}}}{{\left(K^{h}+{c(t,\theta )}^{h}\right)}^{\text{2}}} \end{array}$$ (10)

According to Eq (7), the gradient of the drug effect E(c(t)) with respect to the state vector x(t,θ) (drug amounts) can be derived based on the chain rule as


$$\begin{array}{c} \frac{\partial E\left(c\left(t,\theta \right)\right)}{\partial x(t,\theta )}=\frac{\partial C^{\text{T}}x(t,\theta )}{\partial x(t,\theta )}\frac{\partial E\left(c\left(t,\theta \right)\right)}{\partial c(t,\theta )}=C\frac{\partial E\left(c\left(t,\theta \right)\right)}{\partial c(t,\theta )} \end{array}$$ (11)

Furthermore, the gradient of the drug effect E(c(t,θ)) with respect to the parameter vector θ can be derived based on the chain rule as


$$\begin{array}{c} \frac{\partial E\left(c\left(t,\theta \right)\right)}{\partial \theta }={\left(\frac{\partial x(t,\theta )}{\partial \theta }\right)}^{\text{T}}C\frac{\partial E\left(c\left(t,\theta \right)\right)}{\partial c(t,\theta )}={\left(C^{\text{T}}\left(\frac{\partial x(t,\theta )}{\partial \theta }\right)\right)}^{\text{T}}\frac{\partial E\left(c\left(t,\theta \right)\right)}{\partial c(t,\theta )} \end{array}$$ (12)

where $\frac{\partial x(t)}{\partial \theta }\in R^{n\times pn_{D}}$ is the state-parameter Jacobian matrix defining the sensitivity of the i-th state variable $x_{i}(t)$ to the variations of the j-th parameter such that


$$\begin{array}{c} \frac{\partial x(t,\theta )}{\partial \theta }=(\begin{array}{c} \frac{\partial x(t,\theta )}{\partial \theta^{\text{1}}}\\ \frac{\partial x(t,\theta )}{\partial \theta^{\text{2}}}\\ \frac{\partial x(t,\theta )}{\partial \theta^{\text{3}}}\\ \vdots \\ \frac{\partial x(t,\theta )}{\partial \theta^{n_{D}}} \end{array})=(\begin{array}{c} \frac{\partial x^{\text{1}}\left(t,\theta^{\text{1}}\right)}{\partial \theta^{\text{1}}}\\ \frac{\partial x^{\text{2}}\left(t,\theta^{\text{2}}\right)}{\partial \theta^{\text{2}}}\\ \frac{\partial x^{\text{3}}\left(t,\theta^{\text{3}}\right)}{\partial \theta^{\text{3}}}\\ \vdots \\ \frac{\partial x^{n_{D}}\left(t,\theta^{n_{D}}\right)}{\partial \theta^{n_{D}}} \end{array}) \end{array}$$ (13)

### Solution of the sensitivity model

Consider the vector (sensitivity function) $S_{j}^{i}\left(t,\theta^{i}\right)\in R^{n}$, which quantifies the sensitivity of the state response x(t,θ) with respect to the changes of the j-th parameter in the parameter subvector $\theta^{i}$ corresponding to the i-th dose in terms of Eq (3) such as [40].


$$\begin{array}{c} S_{j}^{i}\left(t,\theta^{i}\right)=\frac{\partial x(t,\theta )}{\partial \theta_{j}^{i}}=\frac{\partial x^{i}\left(t,\theta^{i}\right)}{\partial \theta_{j}^{i}} \end{array}$$ (14)

The sensitivity function can be understood as a quantitative measure of how strongly a change in a particular PK parameter influences the resulting drug amounts in each compartment over time. In practical terms, it answers a simple but clinically meaningful question: “If we slightly modify this parameter how much will the concentration–time profile change?”

The full sensitivity matrix $\frac{\partial x(t,\theta )}{\partial \theta }\in R^{n\times pn_{D}}$ defined by Eq (13) is then formed as


$$\begin{array}{c} \frac{\partial x(t,\theta )}{\partial \theta }=(\begin{array}{ccccccccc} S_{\text{1}}^{\text{1}}\left(t,\theta^{\text{1}}\right) & \cdots & S_{p}^{\text{1}}\left(t,\theta^{\text{1}}\right)S_{\text{1}}^{\text{2}}\left(t,\theta^{\text{2}}\right) & \cdots & S_{p}^{\text{2}}\left(t,\theta^{\text{2}}\right) & \cdots & S_{\text{1}}^{n_{D}}\left(t,\theta^{n_{D}}\right) & \cdots & S_{p}^{n_{D}}\left(t,\theta^{n_{D}}\right) \end{array}) \end{array}$$ (15)

The solution of the sensitivity function $S_{j}^{i}\left(t,\theta^{i}\right)$ can be obtained by utilizing its time derivative ${\dot{S}}_{j}^{i}\left(t,\theta^{i}\right)$ which can be derived as


$$\begin{array}{c} {\dot{S}}_{j}^{i}\left(t,\theta^{i}\right)=\frac{\partial \left(\partial x^{i}\left(t,\theta^{i}\right)\right)}{\partial t\partial \theta_{j}^{i}}+\frac{\partial \left(\partial x^{i}\left(t,\theta^{i}\right)\partial x^{i}\left(t,\theta^{i}\right)\right)}{\partial t\partial \theta_{j}^{i}\partial x^{i}\left(t,\theta^{i}\right)} \end{array}$$

$$\begin{array}{c} {\dot{S}}_{j}^{i}\left(t,\theta^{i}\right)=\frac{\partial {\dot{x}}^{i}\left(t,\theta^{i}\right)}{\partial \theta_{j}}+\frac{\partial \partial x^{i}\left(t,\theta^{i}\right)}{\partial t\partial x^{i}\left(t,\theta^{i}\right)}S_{j}^{i}\left(t,\theta^{i}\right) \end{array}$$ (16)

$$\begin{array}{c} {\dot{S}}_{j}^{i}\left(t,\theta^{i}\right)=\frac{\partial {\dot{x}}^{i}\left(t,\theta^{i}\right)}{\partial \theta_{j}^{i}}+\frac{\partial {\dot{x}}^{i}\left(t,\theta^{i}\right)}{\partial x^{i}\left(t,\theta^{i}\right)}S_{j}^{i}\left(t,\theta^{i}\right) \end{array}$$

where $\frac{\partial {\dot{x}}^{i}\left(t,\theta^{i}\right)}{\partial \theta_{j}^{i}}\in R^{n}$ is the gradient of the differential equation of the state response to the i-th dose with respect to $\theta_{j}^{i}$ and $\frac{\partial {\dot{x}}^{i}\left(t,\theta^{i}\right)}{\partial x^{i}\left(t,\theta^{i}\right)}\in R^{n\times n}$ is the Jacobian matrix of the state dynamics.

In the case of the linear state-space model given by Eq (1), we can write $\frac{\partial {\dot{x}}^{i}\left(t,\theta^{i}\right)}{\partial \theta_{j}^{i}}$ as


$$\begin{array}{c} \frac{\partial {\dot{x}}^{i}\left(t,\theta^{i}\right)}{\partial \theta_{j}^{i}}=\frac{\partial \left(A(\theta^{i})x^{i}\left(t,\theta^{i}\right)+B(\theta^{i})u^{i}(t)\right)}{\partial \theta_{j}^{i}}=\frac{\partial A\left(\theta^{i}\right)}{\partial \theta_{j}^{i}}x^{i}\left(t,\theta^{i}\right)+\frac{\partial B\left(\theta^{i}\right)}{\partial \theta_{j}^{i}}u^{i}(t) \end{array}$$ (17)

while the Jacobian $\frac{\partial {\dot{x}}^{i}\left(t,\theta^{i}\right)}{\partial x^{i}\left(t,\theta^{i}\right)}$ is


$$\begin{array}{c} \frac{\partial {\dot{x}}^{i}\left(t,\theta^{i}\right)}{\partial x^{i}\left(t,\theta^{i}\right)}=\frac{\partial \left(A(\theta^{i})x^{i}(t)+B(\theta^{i})u^{i}(t)\right)}{\partial x^{i}\left(t,\theta^{i}\right)}=A\left(\theta^{i}\right) \end{array}$$ (18)

Therefore, the differential equation for the sensitivity function of the linear state space model given by Eq (1) gets


$$\begin{array}{c} {\dot{S}}_{j}^{i}\left(t,\theta \right)=\frac{\partial A\left(\theta^{i}\right)}{\partial \theta_{j}^{i}}x^{i}\left(t,\theta^{i}\right)+\frac{\partial B\left(\theta^{i}\right)}{\partial \theta_{j}^{i}}u^{i}(t)+A\left(\theta^{i}\right)\;S_{j}^{i}\left(t,\theta \right) \end{array}$$ (19)

Finally, to obtain the sensitivities $\;S_{j}^{i}\left(t,\theta \right)$ for all p parameters across $n_{D}$ doses, we will solve the augmented linear models such that


$$\begin{array}{c} \left(\begin{array}{c} {\dot{x}}^{i}(t,\theta )\\ {\dot{S}}_{j}^{i}\left(t,\theta \right) \end{array})=(\begin{array}{cc} A(\theta^{i}) & 0\\ \frac{\partial A\left(\theta^{i}\right)}{\partial \theta_{j}^{i}} & A(\theta^{i}) \end{array})(\begin{array}{c} x^{i}(t,\theta )\\ S_{j}^{i}\left(t,\theta \right) \end{array})+(\begin{array}{c} B(\theta^{i})\\ \frac{\partial B\left(\theta^{i}\right)}{\partial \theta_{j}^{i}} \end{array})u^{i}(t\right) \end{array}$$ (20)

for $i=\text{1},\text{2},\text{3}\ldots n_{D}\;$, j=1,2,3…p subject to initial conditions $\left(\begin{array}{c} x^{i}(\text{0},\theta )\\ 0 \end{array}\right)$.

According to Eq (12), the derivative of E(c(t,θ)) with respect to $\theta_{j}^{i}$ then gets


$$\begin{array}{c} \frac{\partial E\left(c\left(t,\theta \right)\right)}{\partial \theta_{j}^{i}}=C^{\text{T}}S_{j}^{i}\left(t,\theta \right)\frac{\partial E\left(c\left(t,\theta \right)\right)}{\partial c(t,\theta )} \end{array}$$ (21)

### Optimization of the therapeutic response

In clinical practice, every therapeutic intervention implicitly targets a desired profile of the drug effect—a trajectory that reflects how strong the therapeutic action should be. For antibiotics, physicians often aim to maintain the drug effect above the minimum inhibitory concentration for a sufficient duration to suppress bacterial growth without promoting resistance [41,42]. Similarly, analgesic regimens are designed to maintain pain relief above a minimum acceptable threshold while preventing high peaks that could induce sedation or respiratory depression [43,44].

The central aim of this work is to systematically formalize and optimize drug treatments so that patients receive exactly the therapeutic effect they need. Achieving such precision requires finding not only an appropriate sequence of doses but also choosing the pharmacokinetic properties of the drug formulation for each dose—primarily the liberation rate—so that the drug’s concentration–effect dynamics produces the desired response.

Consider N given time points of interest such that $t_{\text{1}}<t_{\text{2}}<t_{\text{3}}\ldots \;t_{N}$, and the corresponding desired therapeutic effect profile $E_{r}(t)$ arranged in a vector ${𝐄}_{r}\in R^{N}$ as


$$\begin{array}{c} {𝐄}_{r}=(\begin{array}{c} E_{r}\left(t_{\text{1}}\right)\\ E_{r}\left(t_{\text{2}}\right)\\ E_{r}\left(t_{\text{3}}\right)\\ \vdots \\ E_{r}\left(t_{N}\right) \end{array}) \end{array}$$ (22)

Vector ${𝐄}_{r}\in R^{N}$ encapsulates the therapeutic goals that clinicians strive to achieve.

The therapeutic (optimization horizon) $t_{N}$ is chosen to cover the patient response to $n_{D}$ dosing events (dosing periods), for example 48 hours.

The model-based prediction of the actual therapeutic profile can be grouped into a vector $𝐄\left(\theta \right)\in R^{N}$ as


$$\begin{array}{c} 𝐄\left(\theta \right)=(\begin{array}{c} {𝐄}_{\text{1}}\left(\theta^{\text{1}}\right)\\ {𝐄}_{\text{2}}\left(\theta^{\text{1}},\theta^{\text{2}}\right)\\ {𝐄}_{\text{3}}\left(\theta^{\text{1}},\theta^{\text{2}},\theta^{\text{3}}\right)\\ \vdots \\ {𝐄}_{n_{D}}\left(\theta^{\text{1}},\theta^{\text{2}},\theta^{\text{3}},\ldots \;\theta^{n_{D}}\right) \end{array})=(\begin{array}{c} E\left(t_{\text{1}},\theta \right)\\ E\left(t_{\text{2}},\theta \right)\\ E\left(t_{\text{3}},\theta \right)\\ \vdots \\ E\left(t_{N},\theta \right) \end{array}) \end{array}$$ (23)

where each subvector ${𝐄}_{i}\left(\theta^{\text{1}},\theta^{\text{2}},\ldots ,\;\theta^{i}\right)\in R^{N/n_{D}}$ corresponds to the therapeutic profile during the i-th dosing period.

To find the optimal parameters of all doses θ, we want to fit the resulting pharmacodynamic effect (therapeutic profile) 𝐄(θ) to the desired profile ${𝐄}_{r}$ in terms of minimizing the sum of squared deviations $𝐄\left(\theta \right)-{𝐄}_{r}$. Additionally, to ensure minimal dose size and minimal technological modifications of the drug, the Tikhonov regularization term will be included.

To this end, consider the cost function $J\left(\theta \right):\;R^{n_{D}p}\to R$ such that


$$\begin{array}{c} J\left(\theta \right)=\frac{\text{1}}{\text{2}}\left({\left(𝐄\left(\theta \right)-{𝐄}_{r}\right)}^{\text{T}}\left(𝐄\left(\theta \right)-{𝐄}_{r}\right)+{\left(\theta -\overset{―}{\theta }\right)}^{\text{T}}\Lambda \left(\theta -\overset{―}{\theta }\right)\right) \end{array}$$ (24)

where $\Lambda \in R^{n_{D}p\times n_{D}p}$, $\Lambda =\Lambda^{\text{T}}$, Λ≻0 is the regularization matrix and $\overset{―}{\theta }\in R^{n_{D}p}$ are the nominal parameters of treatment for the given drug.

In simple terms, the goal of optimization is to choose the drug dose and formulation parameters so that the actual therapeutic effect over time follows as closely as possible the effect profile we want. By the cost function J(θ), we measure how far the achieved effect is from the desired one. At the same time, we also include the regularization term ${\left(\theta -\overset{―}{\theta }\right)}^{\text{T}}\Lambda \left(\theta -\overset{―}{\theta }\right)$ – a safeguard that discourages unnecessarily large doses or excessive deviations from the drug’s standard formulation.

From an optimization standpoint, it is important to emphasize that the drug effect profile 𝐄(θ) follows the nonlinear Hill function E(c(t)) given by Eq (9), which is a compound function of the drug concentration c(t) and the state x(t) as a nonlinear function of the parameters θ as given by Eq (5). Therefore, it can be inferred that 𝐄(θ) is nonlinear with respect to θ, hence the optimization problem (24) must be treated as the nonlinear least squares problem [45].

The gradient $\nabla J\left(\theta \right):\;R^{n_{D}p}\to R^{n_{D}p}$ of the cost function given by Eq (24) then gets


$$\begin{array}{c} \nabla J\left(\theta \right)={\left(\nabla 𝐄\left(\theta \right)\right)}^{\text{T}}\left(𝐄\left(\theta \right)-{𝐄}_{r}\right)+\Lambda \left(\theta -\overset{―}{\theta }\right) \end{array}$$ (25)

where $\nabla 𝐄\left(\theta \right):\;R^{n_{D}p}\to R^{N\times n_{D}p}$ is the Jacobian matrix of therapeutic profile 𝐄(θ).

The condition for unconstrained optimality to be satisfied is


$$\begin{array}{c} \nabla J\left(\theta^{*}\right)={\left(\nabla 𝐄\left(\theta^{*}\right)\right)}^{\text{T}}\left(𝐄\left(\theta^{*}\right)-{𝐄}_{r}\right)+\Lambda \left(\theta^{*}-\overset{―}{\theta }\right)=0 \end{array}$$ (26)

It is obvious that the solution to the optimality condition given by Eq (26) cannot be found analytically. Instead, we opt for iterative numeric optimization. For the approximate calculation of Hessian matrix $\nabla^{\text{2}}J\left(\theta \right):\;R^{n_{D}p}\to R^{n_{D}p\times n_{D}p}$, we will locally linearize 𝐄(θ) around the current guess $\theta_{k}$ in terms of Taylor series such that


$$\begin{array}{c} 𝐄\left(\theta \right)\approx 𝐄\left(\theta_{k}\right)+\nabla 𝐄\left(\theta_{k}\right)\left(\theta -\theta_{k}\right) \end{array}$$ (27)

The approximate Hessian matrix $\nabla^{\text{2}}J\left(\theta \right):\;R^{n_{D}p}\to R^{n_{D}p\times n_{D}p}$ of the cost function given by Eq (24) therefore gets


$$\begin{array}{c} \nabla^{\text{2}}J\left(\theta_{k}\right)\approx {\left(\nabla 𝐄\left(\theta_{k}\right)\right)}^{\text{T}}\nabla 𝐄\left(\theta_{k}\right)+\Lambda \end{array}$$ (28)

The optimization problem can now be solved in terms of the Gauss-Newton iterative algorithm. The update of the optimized variables $\theta_{k+\text{1}}$ can be obtained as the solution of the linear equation system [46]:


$$\begin{array}{c} \left({\left(\nabla 𝐄\left(\theta_{k}\right)\right)}^{\text{T}}\nabla 𝐄\left(\theta_{k}\right)+\Lambda \right)\left(\theta_{k+\text{1}}-\theta_{k}\right)=-{\left(\nabla 𝐄\left(\theta_{k}\right)\right)}^{\text{T}}\left(𝐄\left(\theta_{k}\right)-{𝐄}_{r}\right)-\Lambda \left(\theta_{k}-\overset{―}{\theta }\right) \end{array}$$ (29)

The Gauss–Newton method is particularly suitable in this setting because it exploits the structure of least-squares problems by approximating the Hessian using only first-order derivative information (Jacobian).

To evaluate the Gauss-Newton iteration given by Eq (29), the Jacobian matrix ∇𝐄(θ)of 𝐄(θ) is required, which has the following block matrix structure


$$\begin{array}{c} \nabla 𝐄\left(\theta \right)=(\begin{array}{ccccc} \nabla_{\theta^{\text{1}}}𝐄\left(\theta \right) & \nabla_{\theta^{\text{2}}}𝐄\left(\theta \right) & \nabla_{\theta^{\text{3}}}𝐄\left(\theta \right) & \cdots & \nabla_{\theta^{n_{D}}}𝐄\left(\theta \right) \end{array}) \end{array}$$ (30)

According to Eq (12) and Eq (14), the submatrices $\nabla_{\theta^{i}}𝐄\left(\theta \right)\in R^{N\times p}$ of Jacobian $\nabla 𝐄(\theta )\in R^{N\times n_{D}p}$ corresponding to the individual doses can be conveniently written using the derivatives of the Hill function $\frac{\partial E\left(c(t)\right)}{\partial c(t)}$ given by Eq (10) and the sensitivity functions $S_{j}^{i}\left(t,\theta^{i}\right)$ introduced earlier such that:


$$\begin{array}{c} \nabla_{\theta^{i}}𝐄\left(\theta \right)=(\begin{array}{ccccc} C^{\text{T}}S_{\text{1}}^{i}\left(t_{\text{1}},\theta^{i}\right)\frac{\partial E\left(c\left(t_{\text{1}}\right)\right)}{\partial c(t_{\text{1}})} & C^{\text{T}}S_{\text{2}}^{i}\left(t_{\text{1}},\theta^{i}\right)\frac{\partial E\left(c\left(t_{\text{1}}\right)\right)}{\partial c(t_{\text{1}})} & C^{\text{T}}S_{\text{3}}^{i}\left(t_{\text{1}},\theta^{i}\right)\frac{\partial E\left(c\left(t_{\text{1}}\right)\right)}{\partial c(t_{\text{1}})} & \cdots & C^{\text{T}}S_{p}^{i}\left(t_{\text{1}},\theta^{i}\right)\frac{\partial E\left(c\left(t_{\text{1}}\right)\right)}{\partial c(t_{\text{1}})}\\ C^{\text{T}}S_{\text{1}}^{i}\left(t_{\text{2}},\theta^{i}\right)\frac{\partial E\left(c\left(t_{\text{2}}\right)\right)}{\partial c(t)} & C^{\text{T}}S_{\text{2}}^{i}\left(t_{\text{2}},\theta^{i}\right)\frac{\partial E\left(c\left(t_{\text{2}}\right)\right)}{\partial c(t_{\text{2}})} & C^{\text{T}}S_{\text{3}}^{i}\left(t_{\text{2}},\theta^{i}\right)\frac{\partial E\left(c\left(t_{\text{2}}\right)\right)}{\partial c(t_{\text{2}})} & \cdots & C^{\text{T}}S_{p}^{i}\left(t_{\text{2}},\theta^{i}\right)\frac{\partial E\left(c\left(t_{\text{2}}\right)\right)}{\partial c(t_{\text{2}})}\\ C^{\text{T}}S_{\text{1}}^{i}\left(t_{\text{3}},\theta^{i}\right)\frac{\partial E\left(c\left(t_{\text{3}}\right)\right)}{\partial c(t_{\text{3}})} & C^{\text{T}}S_{\text{2}}^{i}\left(t_{\text{3}},\theta^{i}\right)\frac{\partial E\left(c\left(t_{\text{3}}\right)\right)}{\partial c(t_{\text{3}})} & C^{\text{T}}S_{\text{3}}^{i}\left(t_{\text{3}},\theta^{i}\right)\frac{\partial E\left(c\left(t_{\text{3}}\right)\right)}{\partial c(t_{\text{3}})} & \cdots & C^{\text{T}}S_{p}^{i}\left(t_{\text{3}},\theta^{i}\right)\frac{\partial E\left(c\left(t_{\text{3}}\right)\right)}{\partial c(t_{\text{3}})}\\ \vdots & \vdots & \vdots & \ddots & \vdots \\ C^{\text{T}}S_{\text{1}}^{i}\left(t_{N},\theta^{i}\right)\frac{\partial E\left(c\left(t_{N}\right)\right)}{\partial c(t_{N})} & C^{\text{T}}S_{\text{2}}^{i}\left(t_{N},\theta^{i}\right)\frac{\partial E\left(c\left(t_{N}\right)\right)}{\partial c(t_{N})} & C^{\text{T}}S_{\text{3}}^{i}\left(t_{N},\theta^{i}\right)\frac{\partial E\left(c\left(t_{N}\right)\right)}{\partial c(t_{N})} & \cdots & C^{\text{T}}S_{p}^{i}\left(t_{N},\theta^{i}\right)\frac{\partial E\left(c\left(t_{N}\right)\right)}{\partial c(t_{N})} \end{array}) \end{array}$$ (31)

It is important to emphasize that the proposed sensitivity analysis is local in nature. Consequently, the Jacobian matrices characterize only local parameter influence around the current operating point and parameter estimate.

For strongly nonlinear PK–PD systems or large parameter deviations, local sensitivities may not fully capture global parameter interactions or multimodal optimization structure. Nevertheless, for the considered class of compartmental PK–PD models operating within clinically relevant concentration ranges, local sensitivity information provides an efficient and computationally tractable approximation enabling structured Gauss–Newton optimization.

The inclusion of the Tikhonov regularization term in Eq (24) is important not only from a therapeutic standpoint, but also from a numerical inverse-problem perspective. Besides penalizing excessively aggressive dosing strategies, the regularization improves conditioning of the approximate Hessian matrix$\;{\left(\nabla 𝐄\left(\theta_{k}\right)\right)}^{\text{T}}\nabla 𝐄\left(\theta_{k}\right)+\Lambda$ and reduces sensitivity of the optimization to parameter correlations and local Jacobian degeneracies.

## Results and discussion

### Compartmental model

For validation of the proposed methodology, we will consider a particular four-compartment pharmacokinetic model describing the drug liberation, absorption, elimination, and the transport mechanisms via four state variables in terms of general linear state-space model given by Eq (1) such that


$$\begin{array}{c} \left(\begin{array}{c} {\dot{x}}_{\text{1}}(t)\\ {\dot{x}}_{\text{2}}(t)\\ {\dot{x}}_{\text{3}}(t)\\ {\dot{x}}_{\text{4}}(t) \end{array})=(\begin{array}{cccc} {-k}_{l} & \text{0} & \text{0} & \text{0}\\ k_{l} & {-k}_{e\text{2}}-k_{a} & \text{0} & \text{0}\\ \text{0} & k_{a} & -k_{\text{3}\text{4}} & \text{0}\\ \text{0} & \text{0} & k_{\text{3}\text{4}} & -k_{e\text{4}} \end{array})(\begin{array}{c} x_{\text{1}}(t)\\ x_{\text{2}}(t)\\ x_{\text{3}}(t)\\ x_{\text{4}}(t) \end{array})+(\begin{array}{c} D\\ \text{0}\\ \text{0}\\ \text{0} \end{array})u(t\right) \end{array}$$ (32)

where $A\in R^{\text{4}\times \text{4}}$, $B\in R^{\text{4}}$, u∈R, $x\in R^{\text{4}}$, and the state variable $x_{\text{1}}(t)$ [mg] represents the amount of the unliberated drug in the stomach, $x_{\text{2}}(t)$ [mg] is the liberated drug amount in the gastrointestinal tract, $x_{\text{3}}(t)$ [mg] represents the amount of absorbed drug in the central compartment (blood circulation), and $x_{\text{4}}(t)$ [mg] is the drug amount in the peripheral compartment (site of drug action where receptors are present), D [mg] is the dose size, while u(t) [1/h] is given by Eq (2). This model has been inspired by our previous works [4,5,47], and modified by adding the drug liberation dynamics.

The parameters $k_{l}$, $k_{a}$, $k_{e\text{2}}$, $k_{\text{3}\text{4}}$, and $k_{e\text{4}}$ [1/h] are real positive constants. The parameter $k_{l}$ represents the liberation rate, and the parameter $k_{a}$ quantifies the rate of drug absorption from the gastrointestinal tract. The parameters $k_{e\text{2}}$, $k_{e\text{4}}$ affect the rates of the drug elimination from the compartments $x_{\text{2}}(t)$ and $x_{\text{4}}(t)$, and $k_{\text{3}\text{4}}$ determines the rate of the inter-compartment drug transport (flow) from $x_{\text{3}}(t)$ to $x_{\text{4}}(t)$.

The first two compartments describe the liberation of the drug from its formulation and its presence in the gastrointestinal tract, where absorption occurs. The liberation process is strictly unidirectional: once liberated, the drug cannot return to its initial bound state, reflecting a physically irreversible transition.

The liberated drug enters the second compartment, which represents the accessible drug mass in the gastrointestinal tract. From this compartment, two distinct physiological pathways emerge. First, the absorption flow governed by $k_{a}$ transports drug molecules across the intestinal membrane into the systemic circulation. The second term captures small but physiologically plausible losses (e.g., degradation or insufficient absorption).

The third compartment represents the central systemic circulation, and it plays a pivotal causal role in determining therapeutic exposure. All drug that reaches this compartment is considered bioavailable, and its magnitude directly influences both downstream receptor engagement and systemic elimination. The inflow into the third compartment is entirely determined by the absorption rate, creating a clear and physiologically grounded causal dependency: the faster the absorption from the gastrointestinal tract, the more rapid the rise in systemic concentration. The drug molecules in the central compartment then undergo two competing processes. The first is inter-compartmental transport toward the receptor compartment. This flow reflects the distribution into peripheral tissues or specific receptor sites, depending on the drug’s mechanism of action. The second competing process is systemic elimination. Although not explicitly included in the transport term, central elimination is effectively modeled within the flow structure to reflect physiological clearance mechanisms such as hepatic metabolism or renal excretion.

The fourth compartment corresponds to the receptor-site compartment. It receives drug exclusively through the inter-compartmental flow. This strict causal direction embodies the pharmacological principle that receptor-site exposure depends entirely on systemic availability. Once in the receptor compartment, the drug can either bind to receptors or undergo elimination, with the latter modeled as a first-order process.

The optimized PK parameters include the dose size D and its liberation rate $k_{l}$, hence p=2 and the parameter vector θ gets


$$\begin{array}{c} \theta =(\begin{array}{c} D\\ k_{l} \end{array}) \end{array}$$ (33)

This choice is strongly motivated by established clinical practice and contemporary pharmaceutical technology. In real-world oral drug administration, each dose can be individually tailored with respect to both amount and formulation. Dose magnitude is the principal control variable clinicians use to regulate systemic drug exposure, and individualized dose titration is a well-established cornerstone of clinical pharmacotherapy [2,3].

Equally important, the liberation rate is increasingly recognized as a tunable parameter due to advancements in formulation science. Modern drug delivery technologies—including controlled-release matrices, enteric coatings, multilayer tablets, microencapsulation, and polymer-based delivery systems [48,49] —enable precise manipulation of dissolution and release profiles for each administered dose [50]. As a result, distinct doses of the same drug can be engineered to exhibit specific release kinetics tailored to therapeutic goals such as delay of onset, extension of exposure, or reduction of peak-related adverse effects.

From a modeling standpoint, optimizing these parameters directly influences the entire causal flow of the PK chain: the dose size scales the amplitude of the system response, whereas the liberation rate governs the temporal profile of drug availability for absorption, effectively acting as the inverse of a time constant.

In contrast to the modifiable parameters, the remaining pharmacokinetic parameters—namely the absorption rate, the elimination rates, and the inter-compartment transfer rate, cannot be considered optimization variables within a therapeutic design framework. These parameters represent intrinsic physiological or biochemical processes that are fundamentally determined by the patient’s biology and the molecular properties of the drug. For example, the absorption rate depends on factors such as intestinal membrane permeability, gastrointestinal motility, transporter expression, and local blood flow—all of which are governed by the patient’s physiology and cannot be manipulated on a per-dose basis. Similarly, elimination constants reflect hepatic metabolism, renal clearance, and systemic enzymatic degradation. The inter-compartmental transfer rate is likewise determined by the physicochemical properties of the molecule—lipophilicity, binding affinity, tissue permeability, and receptor-site kinetics.

### Experiment setup

The values of the fixed PK parameters are adopted from [4,37,47] as $k_{a}\;=\;\text{0}.\text{0}\text{3}\text{7}\text{0}$ 1/h, $k_{e\text{2}}\;=\;\text{0}.\text{1}\text{2}\text{1}\text{4}$ 1/h, $k_{\text{3}\text{4}}\;=\;\text{1}.\text{2}\text{7}\text{2}\text{5}$ 1/h, $k_{e\text{4}}\;=\;\text{0}.\text{2}\text{1}\text{7}\text{1}$ 1/h.

The distribution volume V of the peripheral compartment in Eq (8) was assumed as V=5000 ml, hence vector C gets


$$\begin{array}{c} C=(\begin{array}{c} \text{0}\\ \text{0}\\ \text{0}\\ \frac{\text{1}}{\text{5}\text{0}\text{0}\text{0}} \end{array}) \end{array}$$ (34)

Considering the model given by Eq (32) and the parameter vector given by Eq (33), derivatives $\frac{\partial A\left(\theta^{i}\right)}{\partial \theta_{j}^{i}}$, $\frac{\partial B\left(\theta^{i}\right)}{\partial \theta_{j}^{i}}$ in Eq (17) are as follows


$$\begin{array}{c} \frac{\partial A\left(\theta^{i}\right)}{\partial \theta_{\text{1}}^{i}}=(\begin{array}{cccc} \text{0} & \text{0} & \text{0} & \text{0}\\ \text{0} & \text{0} & \text{0} & \text{0}\\ \text{0} & \text{0} & \text{0} & \text{0}\\ \text{0} & \text{0} & \text{0} & \text{0} \end{array}),\frac{\partial B\left(\theta^{i}\right)}{\partial \theta_{\text{1}}^{i}}=(\begin{array}{c} \text{1}\\ \text{0}\\ \text{0}\\ \text{0} \end{array})\\ \frac{\partial A(\theta^{t})}{\partial \theta_{2}^{t}}=(\begin{array}{cccc} -1 & 0 & 0 & 0\\ 1 & 0 & 0 & 0\\ 0 & 0 & 0 & 0\\ 0 & 0 & 0 & 0 \end{array}),\;\frac{\partial B(\theta^{t})}{\partial \theta_{2}^{t}}=(\begin{array}{c} 0\\ 0\\ 0\\ 0 \end{array}). \end{array}$$ (35)

The half-saturation constant K and the Hill coefficient h in Eq (9) were set to $K={\text{1}\text{0}}^{-\text{4}}$ mg/ml, h=2.

**Fig 1:**
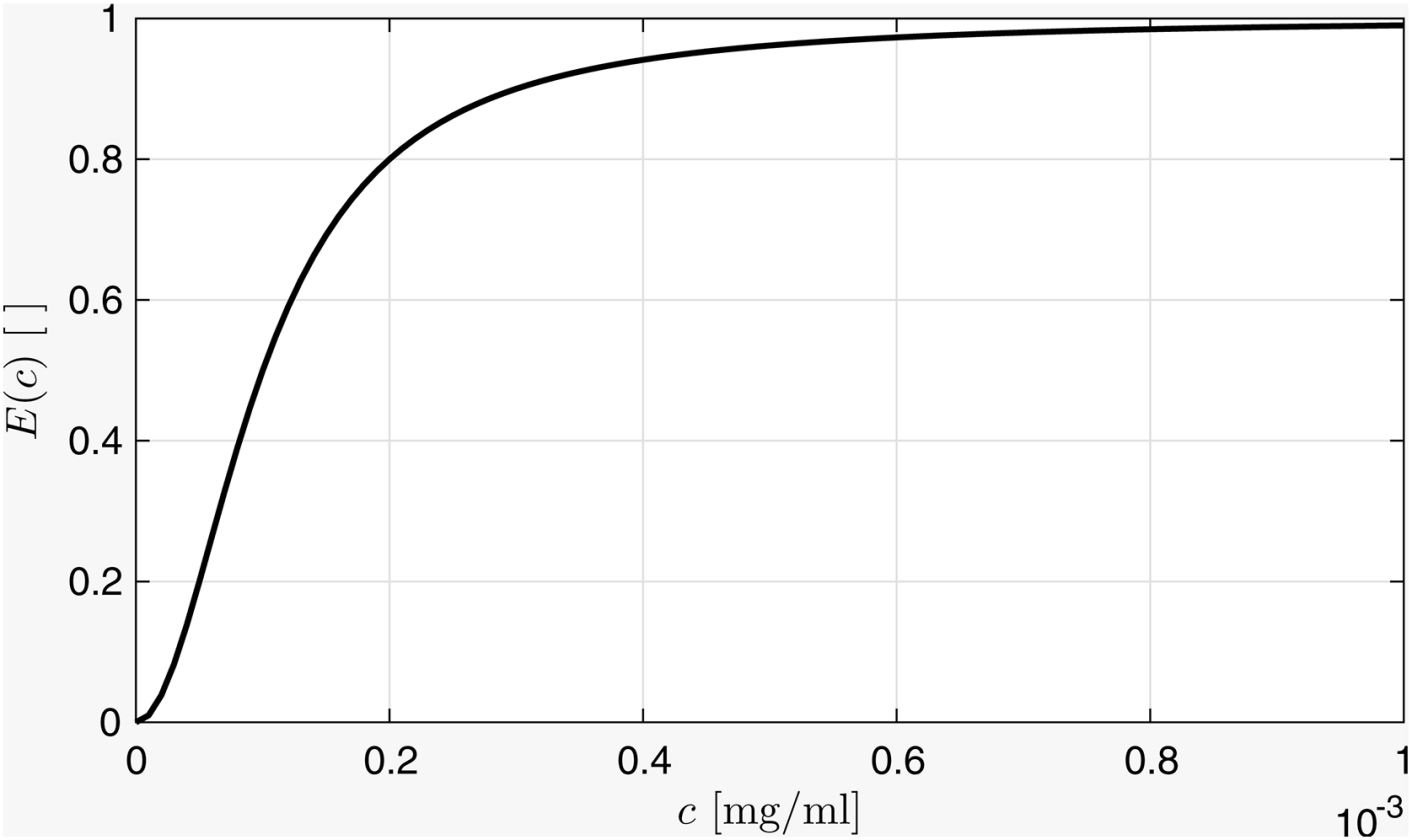
Hill function *E* (*c*).

The half-saturation constant $K={\text{1}\text{0}}^{-\text{4}}\;\text{mg}/\text{ml}$ is a value consistent with mid-micromolar potencies commonly observed for small-molecule agents. The Hill coefficient was set to h=2, representing a moderately steep and cooperative concentration–effect relationship that is consistent with many therapeutic agents exhibiting partial receptor cooperativity or nonlinear amplification in their downstream pharmacodynamic signaling. The corresponding Hill function E(c) is then visualized in Fig 1.

**Fig 2:**
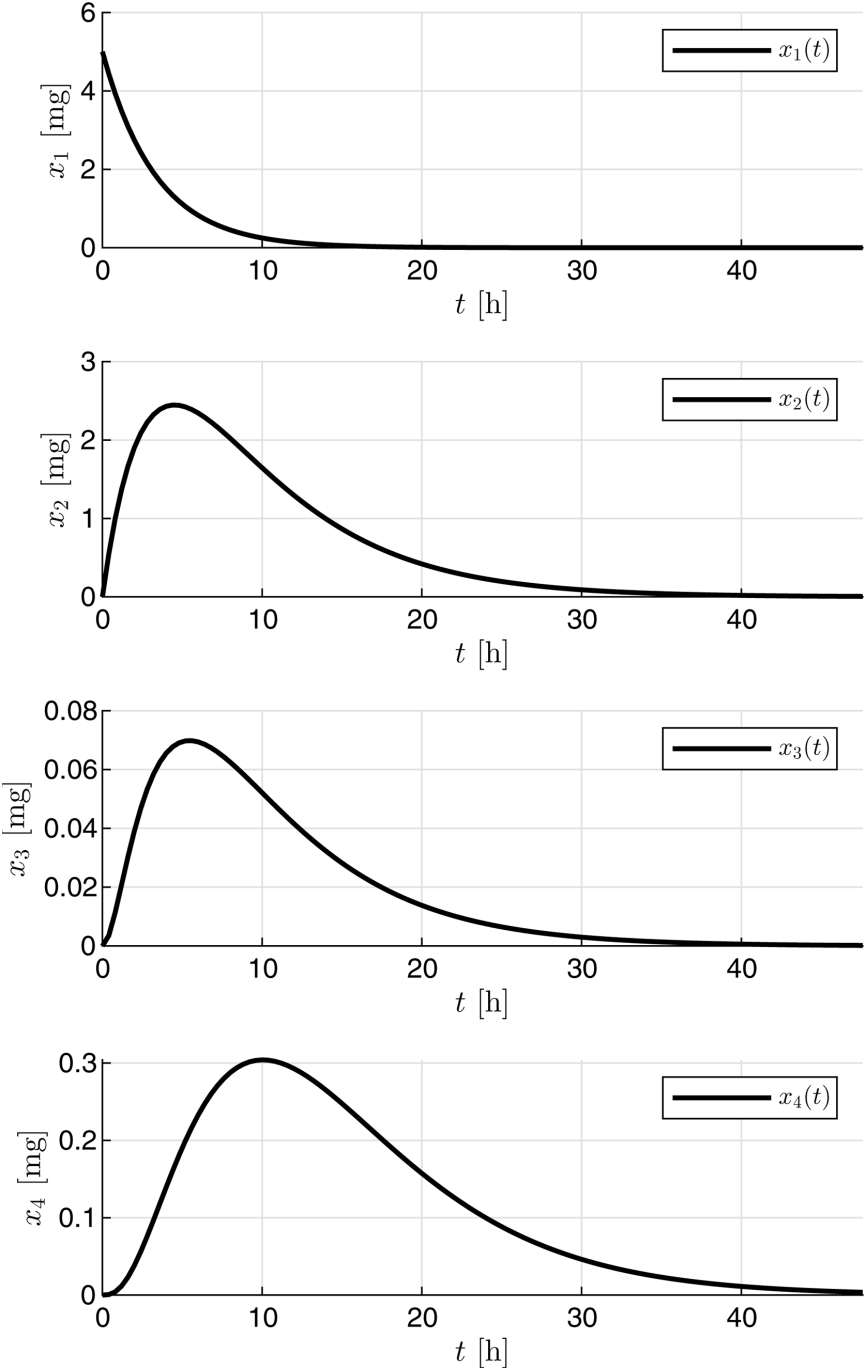
State response to a single suboptimal dose.

For a better conceptualization of the PK characteristics of the considered model, the state response to a single dose D=5.00 mg and liberation rate $k_{l}=\text{0}.\text{3}$ 1/h, is plotted in Fig 2. This state response documents that, after the dose administration, the amount of unliberated drug $x_{\text{1}}(t)$ instantly jumps to 5.00 mg, while the amount of liberated drug $x_{\text{2}}(t)$ starts to rise continuously. The amount of absorbed drug in the bloodstream $x_{\text{3}}(t)$ slightly lags behind $x_{\text{2}}(t)$, while the drug amount in the peripheral compartment $x_{\text{4}}(t)$ lags about 5 hours behind the central compartment and peaks at 10 hours following the dose administration.

**Fig 3:**
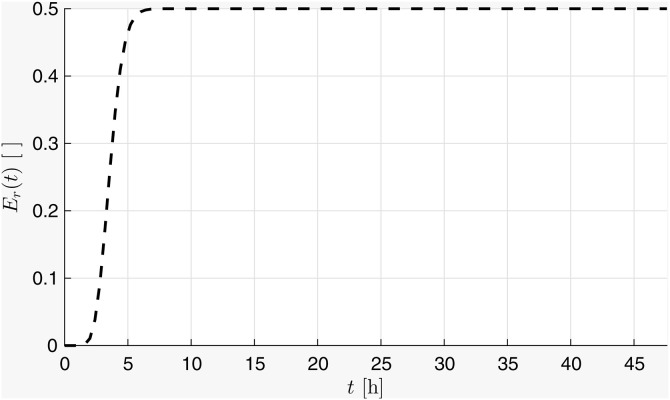
Target therapeutic profile *E_r_(t)*.

For the optimal dosing design, the desired therapeutic profile $E_{r}(t)$ defined in Eq (22) was specified using a smooth, logistic-like function visualized in Fig 3, yielding a therapeutic effect that rises gradually to a clinically meaningful relative steady level E=0.5 (the mid-therapeutic range) with the onset time approximately 6 hours after the leading dose.

The logistic reference trajectory reflects qualitative features commonly desired in pharmacotherapy, namely a limited initial response during drug accumulation, a phase of increasing therapeutic effect as drug exposure approaches effective levels, and a subsequent stabilization of the effect within a desired therapeutic range. Such behavior corresponds to the general objective of dosing regimen design, which aims to achieve therapeutic effect within a reasonable time and maintain it at a stable level while avoiding excessive fluctuations [2,3].

Although such perfect behavior is idealized and cannot be achieved with real-world impulsive dosing, the logistic curve provides a physiologically plausible and clinically interpretable target profile against which optimal dosing and formulation parameters can be designed.

The dosing period is chosen as $T_{D}=\text{8}$ hours, the number of time points equals N=120, and the number of doses is $n_{D}=\text{6}$, which corresponds to the total optimization horizon $t_{N}=\text{4}\text{8}$ hours.

The regularization matrix Λ in Eq (24) was chosen as the block diagonal across all doses


$$\begin{array}{c} \Lambda =\text{blockdiag}(\begin{array}{cc} \text{0}.\text{5}\times {\text{1}\text{0}}^{-\text{3}} & \text{0}\\ \text{0} & \text{0}.\text{5}\times {\text{1}\text{0}}^{-\text{2}} \end{array}) \end{array}$$ (36)

This choice reflects the typical magnitudes of the optimized variables, i.e., the dose size D and the liberation rate $k_{l}$ respectively, such that the regularization of both parameters is balanced.

The nominal parameters $\overset{―}{\theta }$ for the regularization were chosen as


$$\begin{array}{c} \overset{―}{\theta }=(\begin{array}{c} \text{0}\\ \text{0}.\text{3}\\ \text{0}\\ \text{0}.\text{3}\\ \vdots \\ \text{0}\\ \text{0}.\text{3} \end{array}) \end{array}$$ (37)

The choice of the nominal dose size as zero is motivated by the need for minimizing the dose size (regularization toward the zero dose), whereas $k_{l}=\text{0}.\text{3}$ 1/h represents the liberation rate of the standard drug form.

### Experiment results

**Fig 4:**
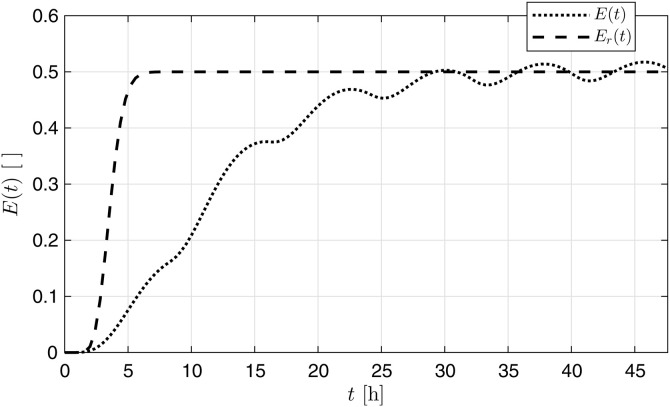
Suboptimal therapeutic profile with fixed dose size *D* = 3.75 mg and liberation rate *k_l_* = 0.3.

**Fig 5:**
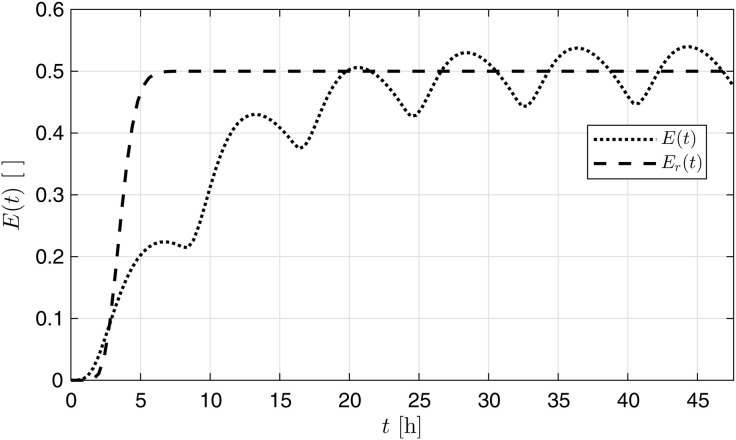
Suboptimal therapeutic profile with fixed dose size *D* = 3.75 mg and fixed liberation rate *k_l_* = 2.5.

For better conceptualization, the drug therapy will be evaluated first considering a standard fixed dose size D=3.75 mg, and the nominal liberation rate $k_{l}=\text{0}.\text{3}$ 1/h to demonstrate a suboptimal therapeutic response. The corresponding Fig 4 then shows a slow onset of the therapeutic effect, taking approximately 30 hours to achieve the desired value, and the presence of mild fluctuations between individual doses (± 0.025). For comparison, by increasing the liberation rate to $k_{l}=\text{2}.\text{5}$ 1/h, the therapeutic response was faster, onset of the therapeutic effect to the desired level took approximately 20 hours, but it was achieved at the expense of more pronounced fluctuations (± 0.05) during the repeated dosing as documented in Fig 5. Both Figs demonstrate the obvious disadvantages of drug dosing with constant dose size and drug form, which constitute the inability to achieve fast onset of the therapy and to simultaneously suppress fluctuations of the therapeutic effect between the doses.

The next scenario considers optimizing the dose sizes by the means of the proposed methodology, but with the liberation rate $k_{l}$ fixed as $k_{l}=\text{0}.\text{3}$, hence p=1 and the parameter vector θ gets θ=D. The purpose of this scenario is to demonstrate the advantages of variable-size optimal dosing over the conventional fixed-size dosing without the need to modify the drug form. The sequence of optimized doses was obtained as


$$\begin{array}{c} \theta =(\begin{array}{c} \begin{array}{cccccc} \text{9}.\text{1}\text{5} & \text{1}.\text{2}\text{8} & \text{4}.\text{4}\text{1} & \text{3}.\text{5}\text{4} & \text{3}.\text{8}\text{0} & \text{3}.\text{4}\text{4} \end{array} \end{array})\text{T} \end{array}$$ (38)

**Fig 6:**
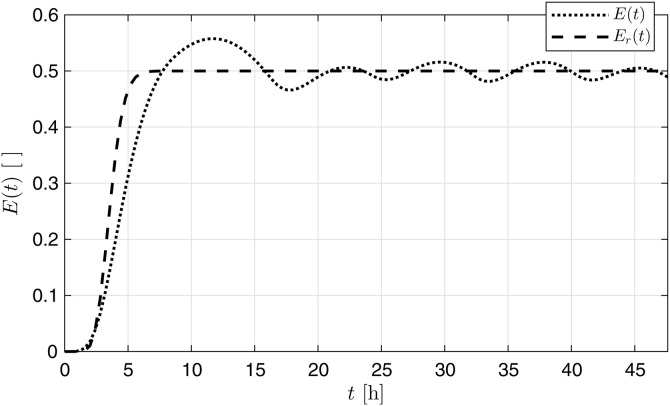
Suboptimal therapeutic profile with optimal variable dose size D mg and fixed liberation rate $k_{l}=0.3$.

It can be inferred that the first (leading) dose is significantly larger than the maintenance doses to ensure fast onset of the therapy, while the second dose turned out to be significantly smaller to prevent the subsequent overshoot and related toxicity. The corresponding therapeutic response can be seen in Fig 6, which demonstrates that optimal therapy with variable dose sizes achieves a fast therapeutic onset (within 7 hours) and provides satisfactory tracking of the desired effect level in the pseudo-steady state. However, the overshoot following the leading dose is too pronounced, which indicates a significant risk of toxicity in the blood circulation and is therefore considered undesired. Moreover, the magnitudes of the fluctuations during the remaining therapeutic windows are still too large, requiring a smoother response to achieve more consistent therapy.

Unlike conventional dosing optimization approaches that treat formulation characteristics as fixed, the proposed framework explicitly includes liberation rate among the optimization variables. Consequently, the optimization simultaneously determines both how much drug should be administered and how rapidly it should become available for absorption.

Finally, by optimizing the full dosing regime, which includes the dose size *D* and the liberation rate *k_l_* for each dosing event in terms of Eq (33), the following parameters were determined:


$$\begin{array}{c} \theta^{\text{1}}=(\begin{array}{c} \text{7}.\text{3}\text{1}\\ \text{0}.\text{8}\text{1} \end{array}),\;\theta^{\text{2}}=(\begin{array}{c} \text{4}.\text{4}\text{4}\\ \text{0}.\text{1}\text{9} \end{array}),\;\theta^{\text{3}}=(\begin{array}{c} \text{3}.\text{8}\text{4}\\ \text{0}.\text{2}\text{0} \end{array}),\;\theta^{\text{4}}=(\begin{array}{c} \text{3}.\text{8}\text{6}\\ \text{0}.\text{1}\text{8} \end{array}),\;\theta^{\text{5}}=(\begin{array}{c} \text{3}.\text{7}\text{9}\\ \text{0}.\text{1}\text{8} \end{array}),\;\theta^{\text{6}}=(\begin{array}{c} \text{3}.\text{7}\text{3}\\ \text{0}.\text{1}\text{6} \end{array}) \end{array}$$ (39)

**Fig 7:**
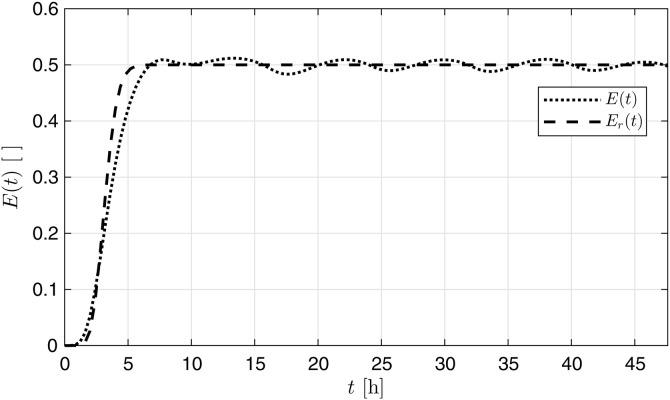
Optimal therapeutic profile with variable dose size *D* mg and dose-dependent liberation rate *k_l_*.

**Fig 8:**
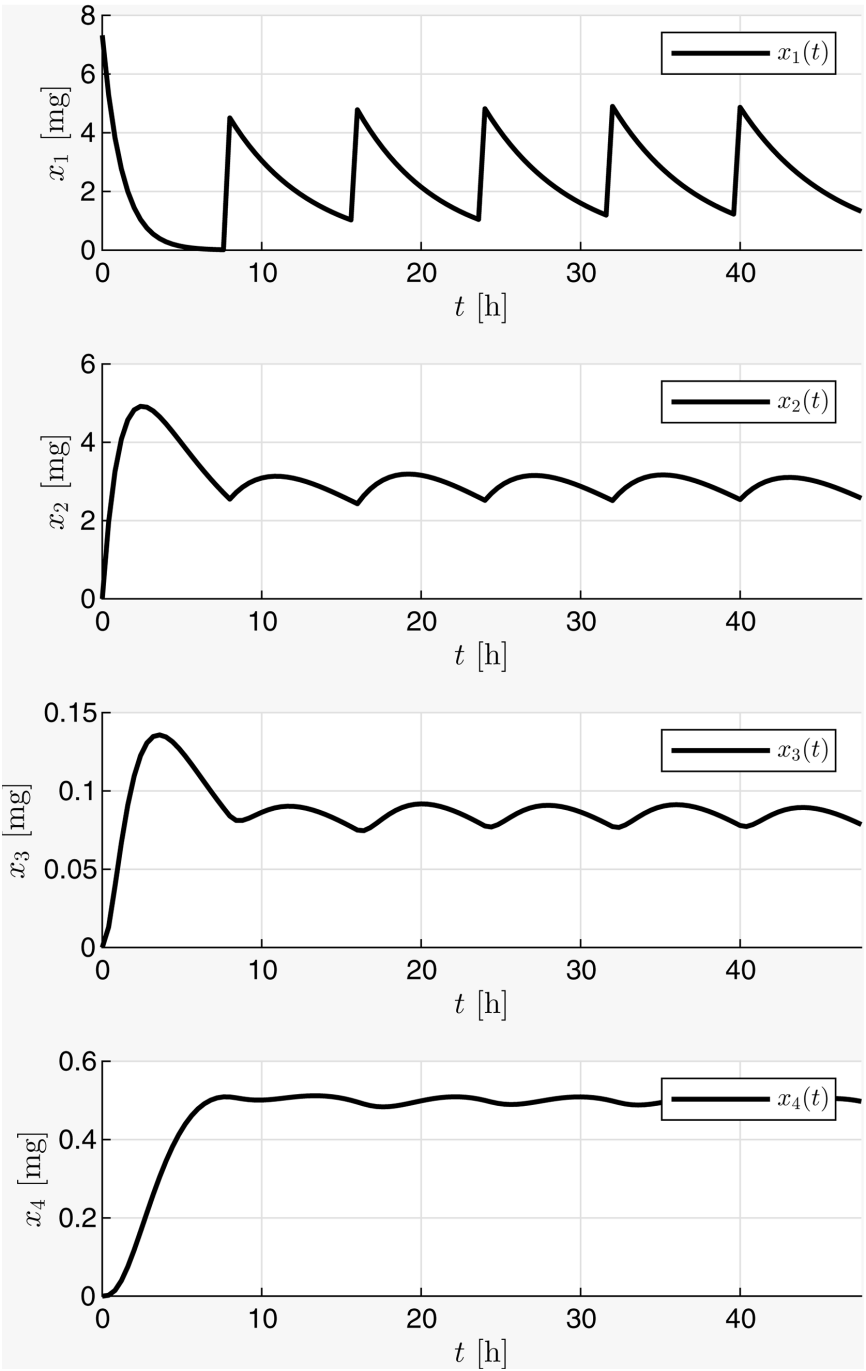
Optimal state response with variable dose size *D* mg and dose-dependent liberation rate *k_l_*.

The corresponding optimal therapeutic profile is then plotted in Fig 7, while the full state response is plotted in Fig 8. The therapeutic profile shows a rapid onset of the effect towards the desired level within 6 hours, practically without an overshoot and the associated risk of toxicity, while the fluctuations during the subsequent therapeutic windows are more suppressed compared to Fig 6. This significant overall improvement is caused by the combined optimization of the dose size and the liberation rate for each dosing event, in such a way that, according to Eq (39), the leading (first) dose is much larger and has a faster liberation than the remaining doses. On the other hand, the maintenance doses are smaller and have slower liberation from the drug form. This dosing strategy leads to a steadier therapeutic response with minimal fluctuation closely resembling the pinnacle performance of continuously adjustable dosing or advanced controlled release drug forms. The state response in Fig 8 then confirms that the liberation following the first dose is faster than the liberation after the remaining doses (faster exponential decay of $x_{\text{1}}(t)$), the state response $x_{\text{3}}(t)$ shows that the drug amount in blood circulation still has an overshoot after the first dose, yet this temporal burst can be considered within safe (non-toxic) interval and is part of the fast-acting therapy. It is also important to emphasize that there is no accumulation of the unliberated drug in the stomach, which might cause digestive issues if the liberation rates are too low.

The comparison between Figs 4–7 highlights the impact of progressively relaxing constraints on the dosing strategy. In the fixed-dose case (Figs 4–5), where identical doses are administered at each dosing event, the system exhibits a clear limitation: achieving faster therapeutic onset requires increasing the effective input intensity, which leads to larger fluctuations and overshoot in the pharmacodynamic response. As a result, the time required to reach the desired therapeutic effect remains on the order of 20–30 hours, depending on the chosen liberation rate, and is accompanied by significant steady-state oscillations.

Allowing variability in dose magnitudes (Fig 6) substantially improves transient performance. In this case, the desired therapeutic effect is reached within approximately 7 hours, representing a reduction in onset time by more than a factor of three compared to fixed-dose regimens. This improvement arises because non-uniform dosing enables an initial loading phase followed by smaller maintenance doses, thereby partially decoupling transient and steady-state behavior. However, fluctuations and overshoot remain relatively pronounced, indicating that dose optimization alone does not fully resolve the underlying dynamic trade-offs.

The fully optimized strategy (Fig 7), in which both dose magnitudes and liberation rates are optimized, provides the best overall performance. The target therapeutic effect is reached within approximately 6 hours, while fluctuations are reduced from approximately ±0.05 in the fixed-dose case to approximately ±0.012. This corresponds to both faster convergence and significantly improved tracking of the desired pharmacodynamic profile.

These results demonstrate that fixed-dose regimens represent a strongly constrained subset of admissible dosing strategies, which inherently limits achievable performance. Relaxing this constraint by allowing variable dose magnitudes introduces additional degrees of freedom that can be exploited to improve transient response, while further optimization of liberation rate enables simultaneous shaping of both transient and steady-state dynamics. Consequently, the proposed framework provides a systematic means of quantifying and exploiting these additional degrees of freedom, revealing performance improvements that are not attainable under conventional fixed-dose assumptions.

Because the resulting optimization problem is generally nonconvex, the Gauss–Newton algorithm provides only local convergence guarantees. To investigate the influence of initialization, the optimization was repeated from 100 randomly perturbed initial parameter vectors. Each initialization was generated by independently perturbing the nominal initial guess for every optimized dose magnitude and liberation rate by a uniformly distributed random factor within ±50% of its nominal value, while keeping the fixed pharmacokinetic parameters unchanged.

**Fig 9:**
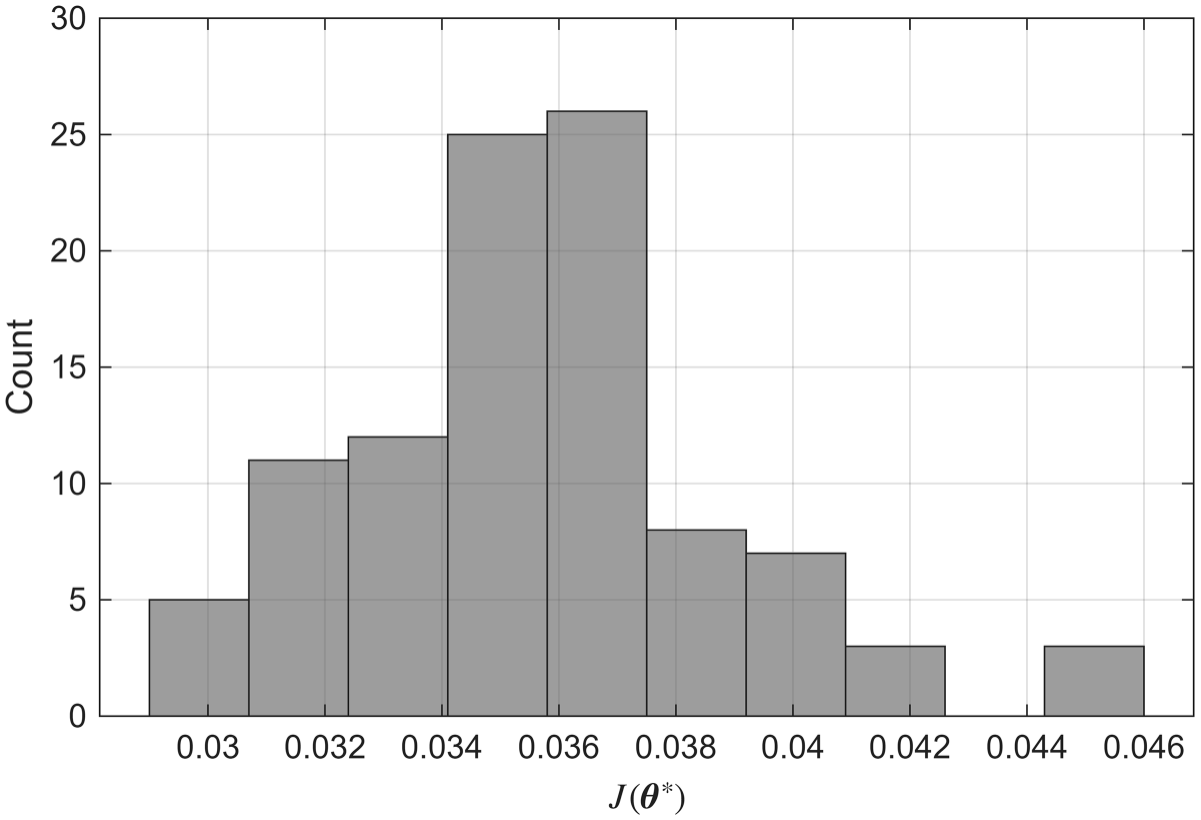
Histogram of the final objective function values obtained from multiple optimization runs initialized from randomly generated parameter vectors.

Fig 9 presents a histogram of the final objective function values obtained from 100 optimization runs initialized from randomly generated parameter vectors. The distribution indicates that despite the substantial variability of the starting points, all optimization runs converged to very similar objective function values and yielded highly similar optimal dosing regimens. This behavior indicates that, within the tested initialization region, the optimization landscape does not exhibit practically significant competing local minima and that the obtained solution is only weakly dependent on initialization. The small differences observed among the final objective function values are likely attributable to the stopping criterion of the Gauss–Newton algorithm, which is based on the norm of the gradient rather than on a prescribed residual tolerance. Consequently, individual runs may terminate at slightly different points within the same local neighborhood of the optimum, leading to minor variations in the final objective values. Although a formal guarantee of global optimality cannot be provided, these results suggest that the proposed sensitivity-based Gauss–Newton procedure exhibits robust convergence toward the same locally optimal solution for clinically relevant initial parameter guess.

To assess the robustness of the proposed optimal dosing framework beyond a single nominal parameter configuration, additional simulations were conducted under a set of different pharmacokinetic settings in terms of Monte Carlo analysis. Specifically, the absorption and elimination rate constants, as well as inter-compartmental transfer rates, were systematically varied to simulate a patient population with varying PK characteristics. The key non-optimizable patient-specific PK parameters, the rate constants $k_{a}$, $k_{e\text{1}}$, $k_{\text{2}\text{3}}$, and $k_{e\text{3}}$, were independently perturbed using a uniform random distribution within ±30 of their nominal values.

In the first scenario, for each of $n_{MC}=\text{5}\text{0}$ realizations, the nonlinear least squares problem of the therapy design was re-solved using the Gauss–Newton optimization framework, resulting in a corresponding personalized optimal dosing policy $\theta^{*}$.

**Fig 10:**
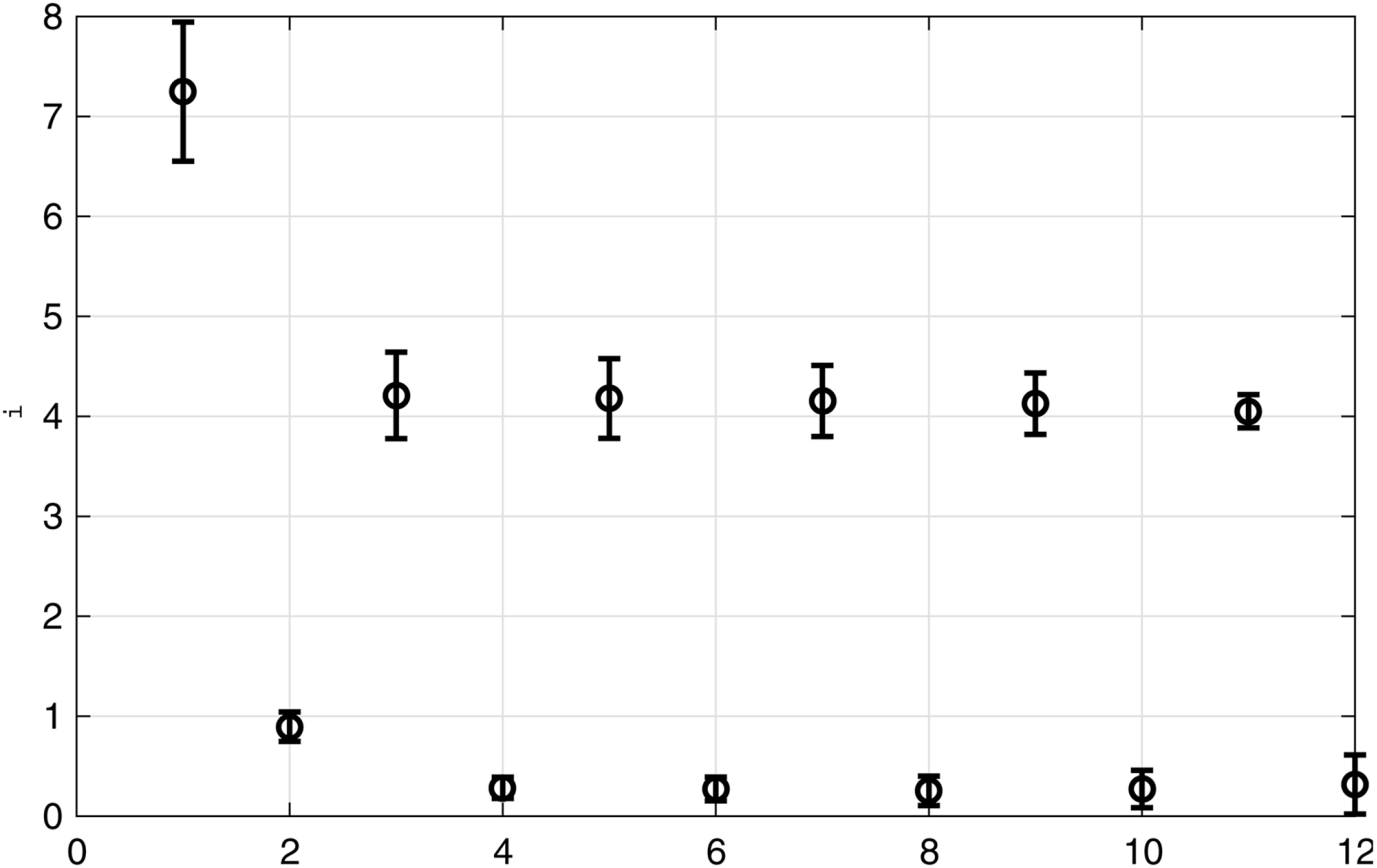
Distribution of optimized treatment parameters $\theta^{*}$ obtained under Monte Carlo analysis of patient population with varying PK characteristics.

**Fig 11:**
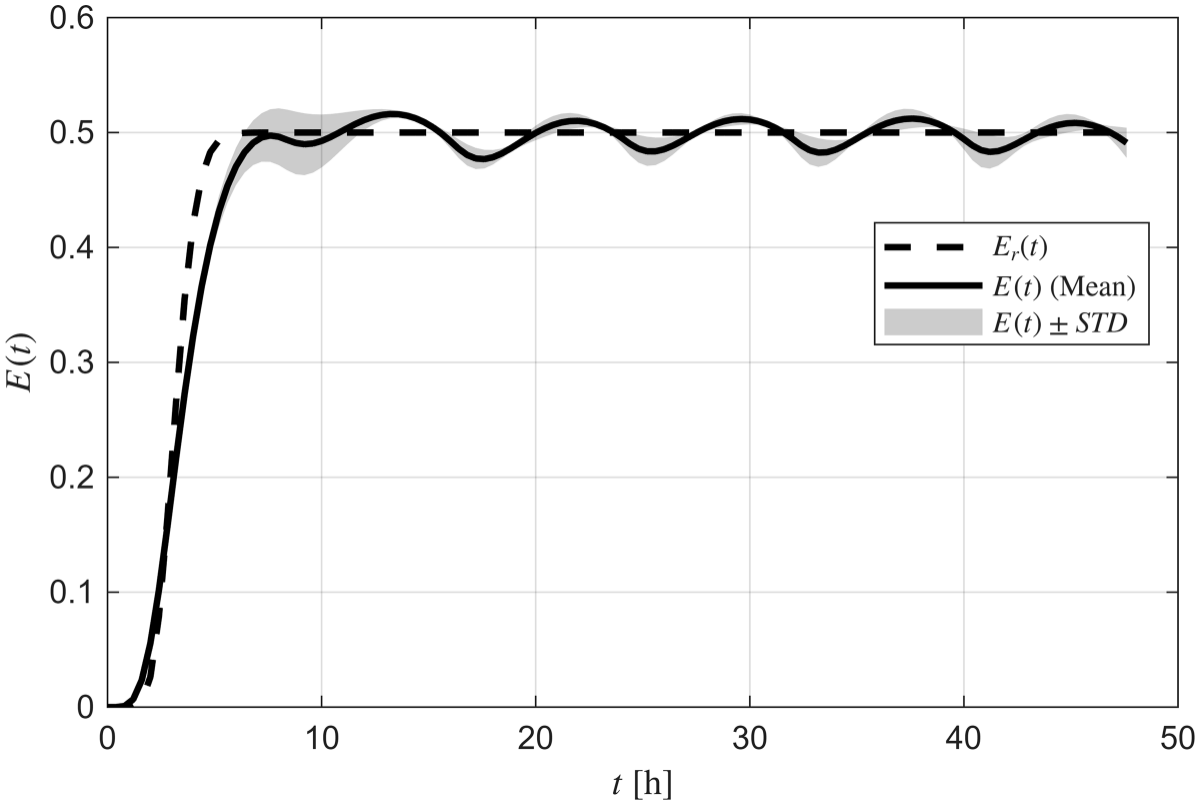
Ensemble mean pharmacodynamic response and its standard deviation obtained under Monte Carlo analysis of patient population with varying PK characteristics and reoptimization.

The resulting distribution of optimized treatment parameters $\theta^{*}$ depicted in Fig 10. indicates that the proposed optimization algorithm remains stable under parameter perturbations, with bounded variability of parameters across all dosing intervals. The most significant variability was observed in the magnitude of the leading dose. As demonstrated in Fig 11., despite variability in the PK dynamics, the resulting pharmacodynamic response E(t) remains closely aligned with the desired reference trajectory $E_{r}(t)$. In particular, the ensemble mean response E(t) (Mean) closely follows the nominal solution from Fig 7., while the standard deviation E(t)± STD remains relatively small throughout the dosing horizon, indicating moderate sensitivity to parameter variations. These results demonstrate that the proposed impulsive PK–PD optimization framework provides consistent therapeutic performance despite variations in pharmacokinetic parameters.

In the second scenario, the nominal-patient optimal therapy defined by Eq. (39) was applied to each of the $n_{MC}=\text{5}\text{0}$ Monte Carlo realizations in order to evaluate the robustness of the dosing recommendation under pharmacokinetic parameter uncertainty.

**Fig 12:**
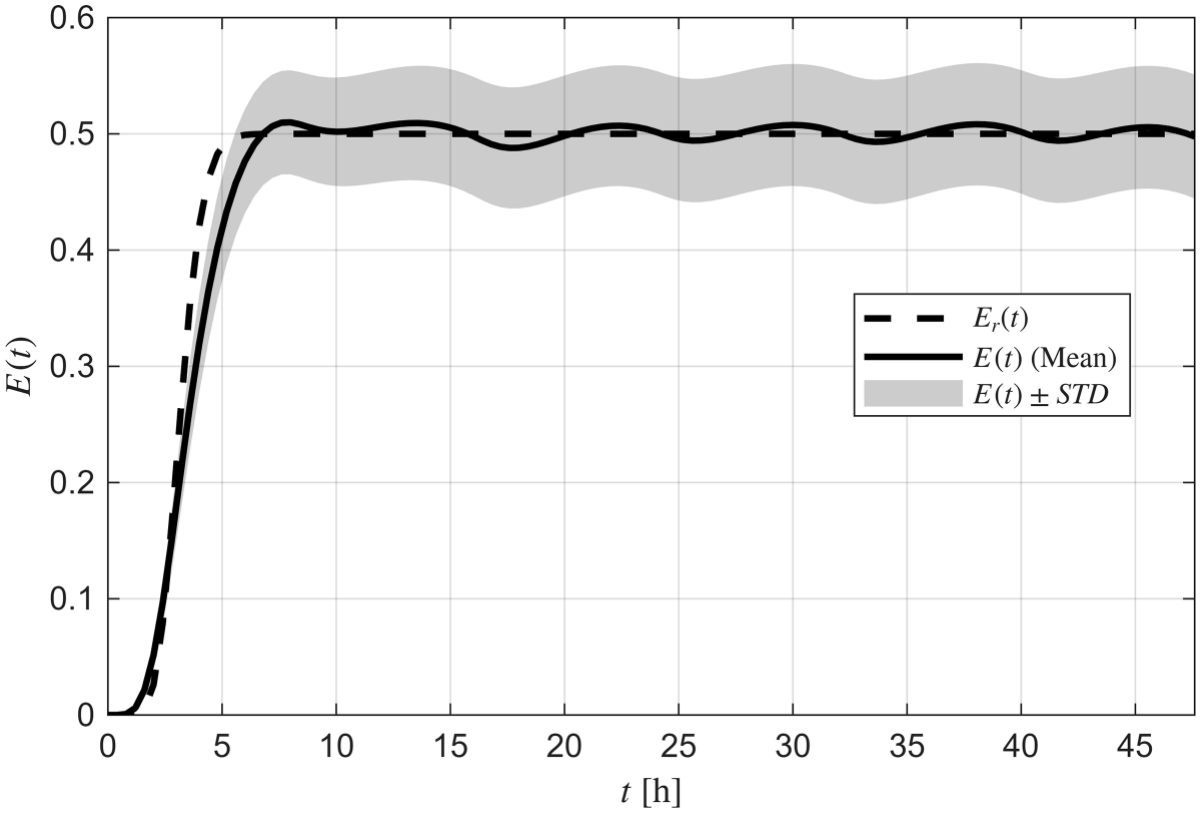
Ensemble mean pharmacodynamic response and its standard deviation obtained under Monte Carlo analysis of patient population with varying PK characteristics and nominal therapy.

As shown in Fig 12, the resulting pharmacodynamic response E(t) remains satisfactory despite the uncertainty in the PK dynamics. However, compared with the personalized optimization results presented in Fig 11, the variability of the response, quantified by the standard deviation E(t)± STD, is increased. The standard deviation reaches approximately ±0.05 in terms of the relative therapeutic effect E(t), corresponding to approximately ±10% variability with respect to the target therapeutic effect 0.5. Despite this increased variability, the pharmacodynamic response remains within an acceptable range, indicating a reasonable degree of robustness of the optimal dosing regimen with respect to pharmacokinetic parameter uncertainty. In particular, the observed deviations do not appear to induce clinically relevant underdosing or toxicity within the considered simulation setting.

As an additional illustrative example, a scenario with impaired drug absorption was considered. This condition was modeled by reducing the absorption rate constant to ($k_{a}=\text{0}.\text{0}\text{1}$ 1/h), representing substantially slower transfer of the drug into the systemic circulation. From a pharmacological perspective, impaired absorption is expected to require larger administered doses to achieve the same therapeutic effect. Furthermore, faster drug liberation may be advantageous to compensate, at least partially, for the delayed absorption and to maintain the onset of action prescribed by the reference pharmacodynamic trajectory ($E_{r}(t)$).

By optimizing the full dosing regime, which includes the dose size D and the liberation rate $k_{l}$ for each dosing event in terms of Eq (33), the following parameters were determined.


$$\begin{array}{c} \theta^{\text{1}}=(\begin{array}{c} \text{24}.\text{65}\\ \text{0}.\text{94} \end{array}),\;\theta^{\text{2}}=(\begin{array}{c} \text{12}.\text{3892}\\ \text{0}.\text{21} \end{array}),\;\theta^{\text{3}}=(\begin{array}{c} \text{11}.\text{57}\\ \text{0}.\text{25} \end{array}),\;\theta^{\text{4}}=(\begin{array}{c} \text{11}.\text{00}\\ \text{0}.\text{24} \end{array}),\;\theta^{\text{5}}=(\begin{array}{c} \text{10}.\text{26}\\ \text{0}.\text{22} \end{array}),\;\theta^{\text{6}}=(\begin{array}{c} \text{8}.\text{99}\\ \text{0}.\text{20} \end{array}), \end{array}$$ (40)

Comparison with the optimal solution presented in Eq (39) shows that the optimized doses are approximately three times larger, while the corresponding liberation rates are approximately 20% higher. These changes are consistent with the reduced absorption capacity of the patient and illustrate how the proposed optimization framework automatically adapts both the administered dose and the formulation-dependent liberation kinetics to maintain the desired therapeutic response.

**Fig 13:**
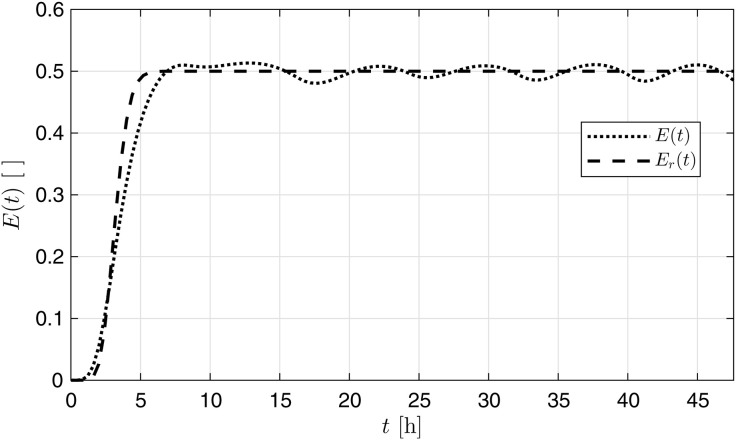
Optimal therapeutic profile with variable dose size *D* mg and dose-dependent liberation rate $k_{l}$ under impaired absorption ($k_{a}=0.01$ 1/h) Model dependence, identifiability, and robustness considerations.

The resulting optimal pharmacodynamic profile is shown in Fig 13. Despite the substantially impaired absorption, the optimized treatment strategy achieves a therapeutic response that remains closely aligned with the reference trajectory ($E_{r}(t)$), exhibiting behavior qualitatively similar to that observed in Fig 7.

The proposed framework is formulated as an open-loop nominal optimization problem and therefore its performance depends on the predictive accuracy of the underlying PK–PD model. Consequently, uncertainties in pharmacokinetic or pharmacodynamic parameters, structural model mismatch, inter-patient variability, adherence deviations, or unmodeled physiological disturbances may affect the resulting therapeutic response.

In particular, inaccuracies in elimination rates, absorption dynamics, receptor sensitivity, or concentration–effect relationships may alter both the transient onset and steady-state behavior of the optimized therapy. Since the present methodology performs optimization offline without feedback correction, such discrepancies may lead to deviations between the predicted and actual pharmacodynamic trajectories.

These limitations are intrinsic to deterministic open-loop optimization approaches relying on predicted system dynamics. Therefore, the present framework should primarily be interpreted as a nominal model-informed optimization methodology rather than a guaranteed robust control strategy.

Nevertheless, the present deterministic formulation constitutes an essential prerequisite for future robustness-oriented extensions. In control-theoretic and model-informed precision dosing frameworks, robust or adaptive optimization fundamentally relies on the existence of an underlying nominal model and corresponding parameter sensitivities.

Importantly, the analytical sensitivity equations derived in this work provide explicit quantitative information regarding how perturbations in PK–PD parameters influence the therapeutic trajectory. This creates a natural foundation for future incorporation of uncertainty-aware methodologies

In particular, repeated therapeutic measurements could be used to periodically update patient-specific PK–PD parameters and re-optimize the remaining dosing strategy online, effectively transforming the present open-loop formulation into an adaptive feedback optimization framework.

From a mathematical standpoint, the present methodology constitutes a structured nonlinear inverse problem. As with all inverse problems, practical limitations may arise due to parameter non-identifiability, parameter correlation, insufficient excitation of the system dynamics, measurement noise, or structural model mismatch.

In particular, if multiple parameter combinations produce similar therapeutic effect trajectories, the Jacobian matrix of the optimization problem may become ill-conditioned or locally rank-deficient. Such situations reduce numerical robustness of the Gauss–Newton iterations and increase sensitivity to perturbations in model parameters or measured data.

Several structural aspects of the proposed framework partially mitigate these difficulties. First, the optimization variables were intentionally restricted to clinically and technologically modifiable parameters, namely dose magnitude and liberation rate. This substantially reduces the dimensionality of the optimization problem and limits parameter correlation. Second, the repeated impulsive excitation generated by multiple dosing events improves distinguishability of transient and steady-state pharmacodynamic behavior. In particular, the liberation rate primarily influences the temporal shape and phase characteristics of the concentration trajectory, whereas the dose magnitude predominantly scales the amplitude of the response.

### Practical implementation and constrained pharmaceutical optimization

In practical pharmaceutical applications, several additional constraints must typically be considered. These include:

• discrete tablet or capsule strengths,
• limited sets of available formulations,
• manufacturable release kinetics,
• excipient and technological limitations,
• and clinically acceptable administration schedules.

Consequently, the present methodology should be interpreted as a continuous relaxation of a more general constrained pharmaceutical co-design problem. Importantly, the proposed framework naturally enables systematic incorporation of such practical constraints.

Discrete dose strengths may be introduced through mixed-integer optimization formulations in which admissible dose values are selected from predefined therapeutic sets. Similarly, finite formulation choices may be represented through discrete candidate release profiles corresponding to available pharmaceutical technologies.

Manufacturability limitations may additionally be incorporated as inequality constraints imposed on liberation dynamics, release-rate bounds, smoothness of dissolution profiles, or achievable kinetic parameters associated with specific drug delivery technologies.

Under such conditions, the resulting optimization problem becomes a hybrid continuous-discrete optimization problem, potentially requiring mixed-integer nonlinear programming (MINLP), branch-and-bound techniques, evolutionary optimization, or hierarchical optimization methodologies.

Nevertheless, the analytical sensitivity framework derived in the present work remains highly valuable even in such constrained settings because it provides local gradient information for the continuous components of the optimization problem and substantially reduces the dimensionality of the clinically relevant decision space.

## Conclusion

This paper presented a methodology for joint optimization of dosing and formulation-dependent release characteristics to achieve a desired therapeutic response in a combined PK–PD framework. The methodology integrated cybernetic principles, a linear state-space compartmental PK model, sensitivity theory, and nonlinear least-squares optimization, with the PD effect modeled by the Hill function. Repeated drug dosing was represented by a sequence of Dirac impulses; hence the problem was addressed within the impulsive systems framework. Sensitivity functions of the state vector with respect to the decision variables were derived, and their time derivatives were used to construct an augmented linear dynamic model necessary for computing the Jacobian matrix of the nonlinear least squares problem. The resulting nonlinear least-squares problem was solved numerically using the Gauss-Newton algorithm. In essence, we formulated the problem of optimal drug dosing within an impulsive system-theoretic framework and combined it with PK–PD modeling to compute the dose size and liberation rate that achieve an optimal clinical effect. These two decision variables were chosen because they can be altered straightforwardly without significantly affecting patient metabolism, homeostasis, or organ function. Importantly, the liberation rate can be manipulated through pharmaceutical technology and drug formulation design [48,49,51].

Unlike classical impulsive control approaches that typically assume fixed doses or uniform release profiles, our method simultaneously optimizes both the per-dose amount and the release kinetics.

The primary methodological contribution is not the introduction of a new PK model, PD model, or optimization algorithm. Rather, it is the integration of these elements into a structured framework that enables simultaneous optimization of dose magnitude and formulation-dependent liberation kinetics through analytically derived sensitivities.

Compared to general-purpose optimal control approaches such as OptiDose, the present framework leverages structural properties of impulsive PK–PD systems. This allows reformulation of the dosing optimization problem as a structured nonlinear least-squares problem with analytically available Jacobians, which in turn facilitates a sensitivity-informed Gauss–Newton optimization. By including formulation-dependent parameters as decision variables, the framework identifies clinically meaningful dosing strategies that emerge naturally from the optimization process, rather than requiring heuristic specification.

Although the optimization problem can be solved offline using standard gradient-free solvers, the present work derives explicit state–parameter sensitivity equations to exploit structural properties of repeated impulsive dosing. Finite-difference approaches do not leverage the linearity of the PK subsystem and may suffer from numerical inaccuracies. The analytical sensitivities provide the exact Jacobian of the pharmacodynamic response with respect to both dose magnitudes and formulation-dependent liberation rates, enabling a structured Gauss–Newton optimization scheme and direct insight into the impact of dosing and release kinetics. Importantly, the approach scales naturally to higher-dimensional compartmental PK models while preserving structured Jacobian computation, making it a foundational tool for future extensions to more complex or disease-integrated models.

The results demonstrated that by systematically optimizing the repeated dosing schedule in terms of dose size and liberation rate, it is possible to achieve the desired onset (speed) of the pharmacodynamic effect while suppressing the fluctuations between the doses typical for repeated drug dosing and oral therapies. Compared with the suboptimal fixed-size dosing, we achieved the desired therapeutic effect much faster, cutting the onset time from 20 to 7 hours, while fluctuations of the relative therapeutic effect ⟨0,1⟩ were reduced from ±0.05 to ±0.015 around the desired value. The results also revealed that the dose-variable liberation rate allows achieving exceptionally fast onset of therapeutic effect (6 hours) with practically no overshoot and fluctuations of the relative therapeutic effect reduced to ±0.012.

An important conclusion is that the optimal therapy consists of a larger leading dose with fast liberation followed by smaller maintenance doses with slower liberation. Crucially, this pattern emerges naturally from the joint optimization of both dose magnitude and formulation-dependent release kinetics under the nonlinear PK–PD model, rather than being imposed a priori or heuristically.

Although the resulting dosing pattern may superficially resemble standard loading-dose or uniform regimens, the similarity is quantitative rather than assumed. Each dose and release profile is the outcome of a structured optimization procedure that explicitly accounts for nonlinear pharmacodynamics and compartmental pharmacokinetics. This approach provides mechanistically justified, flexible, and patient-specific dosing schedules, going beyond the conservative uniform solutions typically produced by classical impulsive control methods.

It is important to differentiate that the proposed method considers completely offline optimization of the drug dosing without accounting for the actual real-time continuous drug monitoring. This contrasts with the classical feedback closed loop control schemes necessitating advanced sensors (continuous drug monitoring) and utilization of the state estimators [47] such as the Kalman filter [52]. However, the nature of the problem eventually allows the estimated state to be seamlessly incorporated, transforming the offline optimization into online optimization where the remaining doses are periodically re-optimized based on the new information obtained from the patient via continuous drug monitoring. This modification would effectively mean augmenting the proposed method towards the closed-loop control

The results suggest that clinicians may benefit from structured optimization of loading and maintenance doses instead of relying solely on empirically derived fixed dosing schedules. Faster therapeutic onset with reduced fluctuations could be especially valuable in conditions requiring rapid and stable drug action, such as pain management [53], immunosuppression [54], or cardiovascular therapies [55]. The approach also highlights the clinical value of controlling the drug liberation rate, which is typically underestimated in standard clinical practice.

For pharmaceutical technologists, the methodology underscores the importance of formulation design as an active control input. Since the liberation rate strongly influences therapeutic performance, pharmaceutical formulations can be engineered not merely for patient convenience but as an integral part of an optimized dosing strategy.

Despite the obvious advantages documented throughout the paper, it is important to acknowledge the identified drawbacks of the proposed methodology.

Therefore, the most significant drawback is clearly the need to accurately determine the parameters of the PK and PD models through in-vivo experiments and system identification methods [56] prior to dosing optimization. Another related issue is that the considered model structure might be inappropriate or insufficient for some drugs which may exhibit different metabolic pathways or receptor mechanisms, rendering the PK or PD part of the model inaccurate and necessitating structural modifications. Fortunately, since the methodology is formulated in sufficient generality (linear state-space model and the concentration–effect function), the optimization algorithm can be easily adapted to any PK compartmental model and any PD concentration–effect static function. Clearly, due to the nature of large clinical trials involved in the process of drug development and due to the use of statistical methods over a large group of patients, the PK–PD models usually have mean-population character, hence neglecting important inter-patient variabilities. Therefore, to achieve patient-specific optimization, prior personalization of the PK–PD model is necessary [57–59].

One of the key challenges in optimal drug therapy is inter-patient variability in pharmacokinetic and pharmacodynamic responses. In the present work, the PK–PD parameters were treated as fixed and representative of a nominal patient in order to focus on the methodological development of the optimization framework. In practical applications, these parameters can be individualized using established approaches such as therapeutic drug monitoring, population PK–PD modeling, Bayesian forecasting, and system identification techniques. Because the proposed optimization relies only on the PK–PD model structure and its sensitivities, the methodology can be directly applied to patient-specific models without modification. Furthermore, repeated measurements of drug concentration or clinical response can be incorporated to periodically update model parameters and re-optimize the remaining dosing strategy, thereby enabling adaptive, closed-loop, and model-informed precision dosing. This highlights that the proposed framework naturally extends from nominal open-loop design to fully individualized therapy.

Patient adherence represents another important consideration in treatment design. Highly complex dosing schedules may reduce compliance and therefore compromise therapeutic effectiveness. The results presented in this work should therefore be interpreted primarily as revealing the intrinsic structure of pharmacologically optimal dosing strategies in the absence of external constraints. In practical settings, the proposed framework allows direct incorporation of clinically motivated constraints, such as limiting the number of distinct dose magnitudes, enforcing constant maintenance dosing, or penalizing variability between consecutive doses. Importantly, this enables explicit characterization of the trade-off between pharmacological optimality and regimen simplicity, rather than addressing adherence considerations heuristically. Such trade-offs are typically difficult to quantify using standard dosing approaches, highlighting a key advantage of the proposed optimization-based formulation.

Pharmaceutical manufacturing imposes inherent constraints on the number of available dose strengths. The optimized dose values obtained in this study should therefore be interpreted as ideal target doses, rather than requiring each dose to correspond to a uniquely manufactured product. In practice, these targets can be implemented by rounding to the nearest commercially available strength, combining tablets of different strengths, or employing tablet splitting where clinically appropriate.

From a methodological perspective, these practical constraints correspond to discrete or mixed-integer restrictions on the optimization variables, limiting admissible doses to a predefined set determined by available pharmaceutical products. Importantly, the underlying PK–PD optimization framework remains unchanged; only the feasible set of decision variables is modified. In this context, continuous optimization serves as an initial design step, revealing the intrinsic structure of optimal therapy, which can subsequently be mapped to implementable dosing regimens without altering the core methodology.

Optimized dosing sequences may involve variable doses across multiple administrations. Such regimens can be implemented safely and efficiently using pre-packaged treatment kits or sequential blister packs, where tablets are arranged and numbered according to the prescribed schedule. Large-scale manufacturing is feasible because the regimen can be realized using a small set of base tablet strengths, combined appropriately to achieve the desired dosing sequence, or through minor controlled variations in tablet mass.

A similar rationale applies to the optimization of drug liberation rates. In the present framework, the liberation rate functions as a design target for formulation development, rather than implying that every individual tablet must possess a uniquely engineered release profile. Modern drug delivery technologies—including controlled-release matrices, polymer-coated pellets, multilayer tablets, osmotic delivery systems, and microencapsulation—allow precise manipulation of drug dissolution and release kinetics. Consequently, treatment regimens can incorporate a small number of formulations with distinct release characteristics, such as an initial fast-release tablet followed by sustained-release maintenance doses.

Taken together, the proposed methodology provides a systematic, quantitative framework for guiding both clinical dose design and pharmaceutical formulation development, while remaining fully compatible with large-scale manufacturing and real-world therapeutic practice.

Future work will focus on optimization of individual dosing times, abandoning the assumption of fixed intervals, to achieve further improvements in therapeutic performance while maintaining analytical tractability. Extending the framework to non-equidistant or adherence-informed dosing schedules is feasible and represents an important direction for clinical translation and adaptive dosing strategies.

Additionally, we want to include the state variables (drug amounts in the individual compartments) into the optimization problem via weighted quadratic penalties. This will enable to tweak and tune all characteristics of the therapy (not only the PD response), including the unliberated and liberated drug amount in the gastrointestinal tract and peaks in the blood circulation.

## References

[1] Yeo K, Berglund E, Chen Y. Dose optimization informed by PBPK modeling: state-of-the art and future. Clin Pharmacol Ther. 2024;116:563–76. doi: 10.1002/cpt.3289 38686708

[2] Rowland M, Tozer TN. Clinical pharmacokinetics and pharmacodynamics: concepts and applications. 4th ed. Wolters Kluwer Health/Lippincott William & Wilkins. 2011.

[3] Bauer LA. Clinical pharmacokinetics and pharmacodynamics. In: DiPiro JT, Yee GC, Posey LM, Haines ST, Nolin TD, Ellingrod V, editors. Pharmacotherapy: A Pathophysiologic Approach. 11e ed. New York, NY: McGraw-Hill Education. 2020.

[4] Vitková Z, Dodek M, Miklovičová E, Pavlovičová J, Babinec A, Vitko A. Robust Control of Repeated Drug Administration with Variable Doses Based on Uncertain Mathematical Model. Bioengineering. 2023;10. doi: 10.3390/bioengineering10080921 PMC10451231 37627806

[5] Dodek M, Vitková Z, Vitko A, Mikloviĉová E. Optimal impulsive control of repeated drug administration. In: 2025. 1–6. 10.1109/PC65047.2025.11047435

[6] Chua HC, Tam VH. Optimizing Clinical Outcomes Through Rational Dosing Strategies: Roles of Pharmacokinetic/Pharmacodynamic Modeling Tools. Open Forum Infect Dis. 2022;9(12):ofac626. doi: 10.1093/ofid/ofac626 36540388 PMC9757694

[7] Chen T, Kirkby NF, Jena R. Optimal dosing of cancer chemotherapy using model predictive control and moving horizon state/parameter estimation. Comput Methods Programs Biomed. 2012;108(3):973–83. doi: 10.1016/j.cmpb.2012.05.011 22739208

[8] Cacace F, Cusimano V, Palumbo P. Optimal impulsive control with application to antiangiogenic tumor therapy. IEEE Transactions on Control Systems Technology. 2020;28:106–17. doi: 10.1109/TCST.2018.2861410

[9] Dodek M, Vitková Z, Vitko A, Pavlovičová J, Miklovičová E. Personalization of optimal chemotherapy dosing based on estimation of uncertain model parameters using artificial neural network. Applied Sciences. 2025;15. doi: 10.3390/app15063145

[10] Dodek M, Vitková Z, Vitko A, Pavlovičová J, Miklovičová E. Optimal Treatment of Tumor Growth, using Individual Chemotherapy Doses. Acta Polytech Hung. 2025;22(7):117–41. doi: 10.12700/aph.22.7.2025.7.7

[11] Pierce JG, Schumitzky A. Optimal impulsive control of compartment models, II: Algorithm. J Optim Theory Appl. 1978;26:581–99. doi: 10.1007/BF00933153

[12] Pierce JG, Schumitzky A. Optimal impulsive control of compartment models, I: Qualitative aspects. J Optim Theory Appl. 1976;18:537–54. doi: 10.1007/BF00932661

[13] Rivadeneira PS, Godoy JL, Sereno JE, Abuin P, Ferramosca A, González AH. Impulsive MPC schemes for biomedical processes: Application to type 1 diabetes. Control Applications for Biomedical Eng Systems. Elsevier. 2020. p. 55–87. doi: 10.1016/b978-0-12-817461-6.00003-2

[14] Villa-Tamayo MF, León-Vargas F, García-Jaramillo M, Rivadeneira PS. Glycemic Control Strategy Based on an Impulsive MPC with Safety Layer Coupling for IOB Limitation. In: 2021 American Control Conference (ACC), 2021. 3372–7. 10.23919/ACC50511.2021.9483402

[15] Dodek M, Miklovičová E. Optimal impulsive disturbance rejection in linear systems with application to diabetes treatment. Comput Methods Programs Biomed. 2025;271:108969. doi: 10.1016/j.cmpb.2025.108969 40773935

[16] Dodek M, Miklovičová E. Optimal model-based insulin bolus advisor for subjects with type 1 diabetes using continuous glucose monitoring. Comput Biol Med. 2025;196(Pt A):110508. doi: 10.1016/j.compbiomed.2025.110508 40639012

[17] Abuin P, Ferramosca A, Rivadeneira PS, Godoy JL, Gonzalez A. Control by pulses under MPC schemes, with applications to artificial pancreas. In: 2019 XVIII Workshop on Information Processing and Control (RPIC). 2019;265–70. doi: 10.1109/rpic.2019.8882137

[18] Sopasakis P, Patrinos P, Sarimveis H, Bemporad A. Model Predictive Control for Linear Impulsive Systems. In: 2012 IEEE 51st IEEE Conference on Decision and Control (CDC), 2012. 5164–9. 10.1109/CDC.2012.6426243

[19] Hungerbühler N. Optimal control in pharmacokinetic drug administration. Math Biosci Eng. 2022;19(5):5312–28. doi: 10.3934/mbe.2022249 35430866

[20] Prodanova K. Optimizing Multiple Drug Administration from Depot by Applying Pharmacokinetic Concepts. Int J Bioautomation. 2020;24(4):337–48. doi: 10.7546/ijba.2020.24.4.000593

[21] Thémans P, Musuamba FT, Winkin JJ. Model-based strategies of drug dosing for pharmacokinetic systems. IFAC-PapersOnLine. 2020;53:16061–8. doi: 10.1016/j.ifacol.2020.12.421

[22] Sopasakis P, Sarimveis H. An integer programming approach for optimal drug dose computation. Comput Methods Programs Biomed. 2012;108(3):1022–35. doi: 10.1016/j.cmpb.2012.06.008 22867981

[23] Bachmann F, Koch G, Pfister M, Szinnai G, Schropp J. OptiDose: computing the individualized optimal drug dosing regimen using optimal control. J Optim Theory Appl. 2021;189(1):46–65. doi: 10.1007/s10957-021-01819-w 34720180 PMC8550736

[24] Drexler DA, Kovács L. Optimization of low dose metronomic therapy based on pharmacological parameters. IFAC-PapersOnLine. 2021;54(15):221–6. doi: 10.1016/j.ifacol.2021.10.259

[25] Hernandez-Mejia G, Hernandez-Vargas EA. PK/PD-based impulsive control to tailor therapies in infectious diseases. IFAC-PapersOnLine. 2020;53:16055–60. doi: 10.1016/j.ifacol.2020.12.418

[26] Lyons MA. Computational pharmacology of rifampin in mice: an application to dose optimization with conflicting objectives in tuberculosis treatment. J Pharmacokinet Pharmacodyn. 2014;41(6):613–23. doi: 10.1007/s10928-014-9380-2 25173151 PMC4578725

[27] Martinez-Vazquez P, Abad-Torrent A. Simultaneous pharmacometric control optimization for advanced integrated multi-drug target infusions. Biomed Signal Process Control. 2025;110:108271. doi: 10.1016/j.bspc.2025.108271

[28] Perez M, Actis M, Sanchez I, Hernandez-Vargas EA, González AH. Multi-objective control to schedule therapies for acute viral infections. J Math Biol. 2025;90(2):25. doi: 10.1007/s00285-025-02188-y 39904788 PMC12119099

[29] Chotsiri P, Yodsawat P, Hoglund RM, Simpson JA, Tarning J. Pharmacometric and statistical considerations for dose optimization. CPT Pharmacometrics Syst Pharmacol. 2025;14(2):279–91. doi: 10.1002/psp4.13271 39501786 PMC11812929

[30] Kovács L, Ferenci T, Gombos B, Füredi A, Rudas I, Szakács G, et al. Positive Impulsive Control of Tumor Therapy—A Cyber-Medical Approach. IEEE Trans Syst Man Cybern, Syst. 2024;54(1):597–608. doi: 10.1109/tsmc.2023.3315637

[31] Medvedev A, Proskurnikov AV, Zhusubaliyev ZT. Design of impulsive feedback controller for dosing. In: 2024. 227–32. 10.1109/MED61351.2024.10566219

[32] Yang Y, Xiao Y, Wang N, Wu J. Optimal control of drug therapy: melding pharmacokinetics with viral dynamics. Biosystems. 2012;107(3):174–85. doi: 10.1016/j.biosystems.2011.11.011 22172775

[33] Luo W, Tan X, Shen J. Optimal treatment strategy for cancer based on mathematical modeling and impulse control theory. Axioms. 2023;12. doi: 10.3390/axioms12100916

[34] Xiong Z, Xia Y, Xue L, Lei J. Mathematical Modelling and Optimization of Medication Regimens for Combination Immunotherapy of Breast Cancer. Bull Math Biol. 2025;87(7):88. doi: 10.1007/s11538-025-01459-5 40455115

[35] Romanick M, Holt A. An ABC of PK/PD. University of Alberta Library. 2023. doi: 10.29173/oer41

[36] Bassingthwaighte JB, Butterworth E, Jardine B, Raymond GM. Compartmental modeling in the analysis of biological systems. Methods Mol Biol. 2012;929:391–438. doi: 10.1007/978-1-62703-050-2_17 23007439

[37] Vitková Z, Tárník M, Pavlovičová J, Murgaš J, Babinec A, Vitko A. In-vivo analysis and model-based prediction of tensides’ influence on drug absorption. Molecules. 2021;26. doi: 10.3390/molecules26185602 PMC8470045 34577073

[38] Ogata K. Modern Control Engineering. 5th ed. Prentice Hall. 2010.

[39] Chen CT. Linear System Theory and Design. 3rd ed. USA: Oxford University Press, Inc. 1998.

[40] Eslami M. The Principal Aspects of Sensitivity Theory. Theory of Sensitivity in Dynamic Systems. Springer Berlin Heidelberg. 1994. p. 17–73. doi: 10.1007/978-3-662-01632-9_2

[41] Roberts JA, De Waele JJ, Dimopoulos G, Koulenti D, Martin C, Montravers P, et al. DALI: Defining Antibiotic Levels in Intensive care unit patients: a multi-centre point of prevalence study to determine whether contemporary antibiotic dosing for critically ill patients is therapeutic. BMC Infect Dis. 2012;12:152. doi: 10.1186/1471-2334-12-152 22768873 PMC3506523

[42] Craig WA. Pharmacokinetic/Pharmacodynamic Parameters: Rationale for Antibacterial Dosing of Mice and Men. Clinical Infectious Diseases. 1998;26:1–12. doi: 10.1086/516284 9455502

[43] Dowell D, Haegerich TM, Chou R. CDC Guideline for Prescribing Opioids for Chronic Pain--United States, 2016. JAMA. 2016;315(15):1624–45. doi: 10.1001/jama.2016.1464 26977696 PMC6390846

[44] Pattinson KTS. Opioids and the control of respiration. Br J Anaesth. 2008;100(6):747–58. doi: 10.1093/bja/aen094 18456641

[45] Chavent G. Nonlinear least squares for inverse problems: theoretical foundations and step-by-step guide for applications. 1st ed. Springer Dordrecht. 2010. doi: 10.1007/978-90-481-2785-6

[46] Computational Methods for Nonlinear Least Squares. Wiley Series in Probability and Statistics. Wiley. 1989. 619–60. doi: 10.1002/0471725315.ch14

[47] Vitková Z, Dodek M, Pavlovičová J, Vitko A. Using a state-bounding observer to predict the guaranteed limits of drug amounts in rats after oral administration based on an uncertain pharmacokinetic model. Pharmaceutics. 2022;14. doi: 10.3390/pharmaceutics14040861 PMC9032709 35456695

[48] Park K. Controlled drug delivery systems: past forward and future back. J Control Release. 2014;190:3–8. doi: 10.1016/j.jconrel.2014.03.054 24794901 PMC4142099

[49] Siepmann J, Peppas NA. Modeling of drug release from delivery systems based on hydroxypropyl methylcellulose (HPMC). Adv Drug Deliv Rev. 2001;48(2–3):139–57. doi: 10.1016/s0169-409x(01)00112-0 11369079

[50] Robinson J, Lee VHL. Controlled drug delivery: fundamentals and applications. 2 ed. Taylor & Francis. 1987.

[51] Aulton ME, Taylor K. Aulton’s pharmaceutics: The design and manufacture of medicines. Churchill Livingstone/Elsevier. 2013.

[52] Anderson BDO, Moore JB. Optimal Filtering. Dover Publications. 2012.

[53] Trescot A, Datta S, Lee M, Hansen H. Opioid Pharmacology. Pain Physician. 2008;11:S133-53. doi: 10.36076/ppj.2008/11/S133 18443637

[54] Staatz CE, Tett SE. Clinical pharmacokinetics and pharmacodynamics of mycophenolate in solid organ transplant recipients. Clin Pharmacokinet. 2007;46(1):13–58. doi: 10.2165/00003088-200746010-00002 17201457

[55] Peeters LEJ, Kester MP, Feyz L, Van Den Bemt PMLA, Koch BCP, Van Gelder T, et al. Pharmacokinetic and pharmacodynamic considerations in the treatment of the elderly patient with hypertension. Expert Opin Drug Metab Toxicol. 2019;15(4):287–97. doi: 10.1080/17425255.2019.1588249 30880496

[56] Ljung L. System identification: theory for the user. 2nd ed. Upper Saddle River, NJ: Prentice Hall. 1999.

[57] Jameson JL, Longo DL. Precision medicine — personalized, problematic, and promising. New England Journal of Medicine. 2015;372:2229–34. doi: 10.1056/NEJMsb1503104 26014593

[58] Hamburg MA, Collins FS. The path to personalized medicine. New England J Med. 2010;363:301–4. doi: 10.1056/NEJMp1006304 20551152

[59] Schork NJ. Personalized medicine: Time for one-person trials. Nature. 2015;520(7549):609–11. doi: 10.1038/520609a 25925459

