# Supplementary material for: Optimal design of dose and drug pharmacokinetic characteristics to achieve the desired pharmacodynamic profile in repeated drug dosing
Source: PLoS One. 2026 Jul 22;21(7):e0354029. doi: 10.1371/journal.pone.0354029 (PMC13390843; doi:10.1371/journal.pone.0354029)
Supplement: Attachment — Submitted filename: Review response.pdf [file pone.0354029.s002.pdf]

Revision notes on the manuscript PONE-D-26-06346 entitled Optimal design of dose and drug pharmacokinetic characteristics to achieve the desired pharmacodynamic profile in repeated drug dosing, submitted to PLOS One

Reviewer 1:

Concern:

The manuscript illustrates the application of control theory to identifying optimal doses, based on pharmacodynamic criteria. However, major challenges for optimising dosing in patients are a) Inter-patient variability in pharmacokinetics and pharmacodynamics, b) Patient compliance (i.e. make the regimen as simple as possible) and c) commercial and logistic feasibility (minimise the number of dose strengths that have to manufactured, shipped and kept in stock by pharmacists). None of these aspects have been considered by this publication.

Response:

We thank the reviewer for raising these important and highly relevant aspects of real-world dose optimization. We fully agree that inter-patient variability, patient adherence, and manufacturing/logistic constraints are central challenges in clinical pharmacotherapy. Rather than being overlooked, these aspects are deliberately decoupled from the core methodological contribution in order to establish a general and extensible optimization framework at the mechanistic PK–PD level.

The primary objective of this work is to formulate dosing design as a well-posed optimization problem over a dynamical PK–PD system. This abstraction is essential: it allows all clinically relevant constraints—whether biological, behavioral, or industrial—to be incorporated in a systematic and modular manner once the underlying optimization structure is established. In this sense, the proposed framework should be understood as a foundational layer upon which increasingly realistic constraints can be progressively imposed without altering the core methodology.

<u>Inter-patient variability.</u>

We agree that variability in PK–PD parameters is a major determinant of clinical dosing strategies. In the present study, parameters are intentionally treated as fixed to isolate and rigorously analyze the structure of the optimal control problem. Importantly, the proposed framework is inherently compatible with all modern approaches to variability handling, including population modeling, Bayesian estimation, and data-driven system identification. Because the optimization relies only on the model structure and its sensitivities, it naturally extends to individualized models obtained from patient-specific data. Moreover, the formulation is directly amenable to adaptive or feedback-based implementations, where

parameter estimates and dosing strategies are updated sequentially as new measurements become available. This positions the framework as a natural building block for closed-loop and personalized therapy design.

Patient adherence and regimen simplicity.

We fully agree that overly complex dosing regimens may reduce adherence and compromise clinical outcomes. It is important to emphasize that the dosing sequences obtained in this work should not be interpreted as prescriptive clinical protocols, but rather as revealing the intrinsic structure of pharmacologically optimal strategies in the absence of external constraints. This distinction is crucial: once the unconstrained structure is understood, practical constraints can be introduced in a controlled and transparent way. In particular, the framework allows direct incorporation of constraints that enforce clinically acceptable regimens, such as limiting the number of distinct dose levels, enforcing constant maintenance dosing, or penalizing variability across dosing events. In this sense, the method provides a principled way to quantify and navigate the trade-off between pharmacological optimality and adherence.

Manufacturing and logistic feasibility.

We agree that real-world implementation is constrained by a limited set of available dose strengths and formulation types. The optimized dose values obtained in this study should therefore be interpreted as continuous design targets, not as requirements for uniquely manufactured products. In practice, these targets can be approximated using standard pharmaceutical strategies, including combinations of available tablet strengths, rounding to discrete dose levels, or tablet splitting where appropriate. From a methodological perspective, such constraints simply restrict the admissible set of dosing decisions, without requiring any modification of the underlying optimization framework. This highlights an important advantage of the proposed approach: it separates the identification of optimal therapeutic structure from the practical constraints of implementation, enabling both to be addressed in a coherent and systematic manner.

A similar interpretation applies to the optimization of drug liberation rate. In this work, the liberation rate serves as a formulation-level design variable that captures the effect of drug delivery kinetics on therapeutic outcome. Modern pharmaceutical technologies provide substantial flexibility in shaping release profiles using a limited number of formulation types. Consequently, the results should be interpreted as guiding formulation design at a conceptual level, rather than implying the need for a distinct formulation for each dosing event.

In summary, the aspects raised by the reviewer are fully compatible with the proposed framework and can be incorporated in a structured and principled manner. The deliberate focus of this work on the mechanistic optimization problem is intended to establish a general methodology that remains valid as additional layers of realism are introduced. We have revised the manuscript to clarify this positioning and to explicitly discuss how variability, adherence, and manufacturing constraints can be integrated within the framework.

We added the following paragraphs into the Conclusion:

One of the key challenges in optimal drug therapy is inter-patient variability in pharmacokinetic and pharmacodynamic responses. In the present work, the PK–PD parameters were treated as fixed and representative of a nominal patient in order to focus on the methodological development of the optimization framework. In practical applications, these parameters can be individualized using established approaches such as therapeutic drug monitoring, population PK–PD modeling, Bayesian forecasting, and system identification techniques. Because the proposed optimization relies only on the PK–PD model structure and its sensitivities, the methodology can be directly applied to patient-specific models without modification. Furthermore, repeated measurements of drug concentration or clinical response can be incorporated to periodically update model parameters and re-optimize the remaining dosing strategy, thereby enabling adaptive, closed-loop, and model-informed precision dosing. This highlights that the proposed framework naturally extends from nominal open-loop design to fully individualized therapy.

Patient adherence represents another important consideration in treatment design. Highly complex dosing schedules may reduce compliance and therefore compromise therapeutic effectiveness. The results presented in this work should therefore be interpreted primarily as revealing the intrinsic structure of pharmacologically optimal dosing strategies in the absence of external constraints. In practical settings, the proposed framework allows direct incorporation of clinically motivated constraints, such as limiting the number of distinct dose magnitudes, enforcing constant maintenance dosing, or penalizing variability between consecutive doses. Importantly, this enables explicit characterization of the trade-off between pharmacological optimality and regimen simplicity, rather than addressing adherence considerations heuristically. Such trade-offs are typically difficult to quantify using standard dosing approaches, highlighting a key advantage of the proposed optimization-based formulation.

Pharmaceutical manufacturing also imposes constraints on the number of available dose strengths. The optimized dose values obtained in the present study should therefore be interpreted as continuous target doses, rather than implying that each dose must correspond to a uniquely manufactured pharmaceutical product. In practice, these targets can be approximated using standard pharmaceutical strategies, including rounding to the nearest available strength, combining multiple tablets, or allowing tablet splitting where clinically appropriate.

From a methodological standpoint, these practical constraints correspond to restricting the admissible set of dosing decisions to a predefined set determined by available pharmaceutical products. This type of constraint is routinely encountered in operations research and pharmaceutical design and can be incorporated within the same optimization framework without altering its fundamental structure. The proposed PK–PD optimization methodology therefore remains unchanged; only the feasible set of decision variables is modified. Continuous optimization can thus be interpreted as an initial design step that reveals the intrinsic structure of the optimal therapy, after which practical implementation constraints can be incorporated in a systematic and transparent manner. This separation between structural optimality and implementation constraints represents an important conceptual advantage of the approach.

In addition to discrete dose strengths, the optimized dosing sequences produced by the framework may involve variation in dose magnitudes across dosing events. Such regimens can nevertheless be implemented in a clinically simple and practical manner using pre-packaged treatment kits or sequential blister packs, where tablets are arranged and numbered according to the prescribed treatment schedule. This allows patients to follow structured but variable regimens with minimal risk

of dosing errors. From a manufacturing perspective, such solutions remain feasible at scale, as tablets can be produced using a limited set of base strengths while the desired dosing sequence is achieved through combinations of these units.

A similar consideration applies to the optimization of the drug liberation rate. In the present framework, the liberation rate should be interpreted primarily as a formulation-level design parameter that captures the influence of drug release kinetics on therapeutic outcome, rather than implying that each individual tablet must possess a uniquely engineered release profile. Modern drug delivery technologies—including controlled-release matrices, polymer-coated pellets, multilayer tablets, osmotic delivery systems, and microencapsulation techniques—provide substantial flexibility in shaping release characteristics using a limited number of formulation types. Consequently, treatment regimens may combine a small set of formulations with distinct release profiles, for example an initial fast-release dose followed by sustained-release maintenance therapy.

Overall, the proposed framework should be interpreted as a foundational optimization layer that separates the intrinsic structure of optimal therapy from application-specific constraints. By decoupling mechanistic modeling, optimization, and implementation considerations, the methodology enables systematic integration of inter-patient variability, adherence requirements, and manufacturing constraints without reformulating the underlying problem. As a result, it provides a unified and extensible quantitative tool for guiding both clinical dose design and pharmaceutical formulation development, while remaining fully compatible with real-world therapeutic practice and large-scale industrial implementation.

Concern:

There are also some useful recent publications that are omitted from the references that would provide a broader view of the research landscape:

1. Chua HC, Tam VH. Optimizing Clinical Outcomes Through Rational Dosing Strategies: Roles of Pharmacokinetic/Pharmacodynamic Modeling Tools. Open Forum Infect Dis. 2022 Dec 16;9(12):ofac626. doi: 10.1093/ofid/ofac626.

2. Chotsiri P, Yodsawat P, Hoglund RM, Simpson JA, Tarning J. Pharmacometric and statistical considerations for dose optimization. CPT Pharmacometrics Syst Pharmacol. 2025; 14: 279-291. doi:10.1002/psp4.13271

Response:

We thank the reviewer for pointing out these relevant recent publications. We agree that including them helps provide a broader perspective on the current research landscape in PK-PD based dose optimization. Both suggested references have now been incorporated into the revised manuscript and discussed in the Introduction to better position our work relative to existing methodologies.

Here are the new paragraphs and the corresponding references:

Classical PK/PD methodologies for dose optimization have been widely applied in antimicrobial therapy and drug development. A large body of work, summarized in [7], emphasizes the

identification of key PK/PD indices such as the ratio of area under the concentration curve to minimum inhibitory concentration (AUC/MIC), peak concentration to MIC (Cmax/MIC), or the duration of time that drug concentration remains above the MIC (T > MIC). These indices are typically determined through dose-fractionation studies and subsequently used as targets for regimen design, with dosing strategies selected to maximize the probability of target attainment in simulated patient populations. This framework has proven highly valuable in clinical pharmacology because it provides a simple and robust way to link drug exposure with antimicrobial efficacy while accounting for interpatient variability through Monte Carlo simulations. However, the PK/PD index paradigm inherently reduces complex pharmacokinetic and pharmacodynamic dynamics to a small number of scalar exposure metrics and therefore provides limited capability for shaping the therapeutic effect. In particular, such approaches typically assume fixed dosing structures and fixed formulation characteristics, and they do not explicitly address transient treatment behavior such as the speed of therapeutic onset, suppression of oscillations between repeated doses, or avoidance of pharmacodynamic overshoot. In contrast, the present work formulates the dosing problem as a dynamical systems optimization problem in which repeated oral dosing is modeled as impulsive inputs to a compartmental pharmacokinetic system coupled with a nonlinear pharmacodynamic response. Rather than targeting exposure thresholds, the proposed framework directly optimizes the time-domain therapeutic effect trajectory.

……….

Advances in model-informed dose optimization have emphasized statistical and pharmacometric approaches that rely on population simulations and exposure matching. In particular, [31] proposed a framework in which candidate dosing regimens are evaluated by comparing the distribution of simulated pharmacokinetic exposure metrics, such as concentration or AUC, to a reference distribution obtained from a clinically validated population. This approach provides a practical and statistically robust solution when pharmacodynamic targets are unknown or poorly characterized. However, distribution-based exposure matching primarily operates on summary pharmacokinetic metrics and does not explicitly account for the temporal dynamics of therapeutic effect. Consequently, it provides limited capability for shaping transient treatment behavior, such as rapid onset of therapy, suppression of oscillations between doses, or control of pharmacodynamic overshoot. In contrast, the present work formulates dose optimization as a dynamical systems problem. Rather than matching exposure distributions, the proposed framework directly optimizes the time-domain therapeutic effect. While the proposed approach requires an explicitly specified pharmacodynamic target profile and relies on accurate PK-PD model identification, it provides a complementary perspective to exposure-matching strategies by enabling direct design of dynamic therapeutic response.

7. Chua HC, Tam VH. Optimizing Clinical Outcomes Through Rational Dosing Strategies: Roles of Pharmacokinetic/Pharmacodynamic Modeling Tools. Open Forum Infect Dis. 2022;9: ofac626. doi:10.1093/ofid/ofac626

31. Chotsiri P, Yodsawat P, Hoglund RM, Simpson JA, Tarning J. Pharmacometric and statistical considerations for dose optimization. CPT Pharmacometrics Syst Pharmacol. 2025;14: 279–291. doi:https://doi.org/10.1002/psp4.13271

Concern:

The proposed optimal solution for the case study proposes administering a different dose level at each administration, which is not particularly feasible. The authors should demonstrate the increase of optimality of this approach vs the same dose level at each administration.

Response:

We thank the reviewer for this important comment. We fully agree that a quantitative comparison between fixed-dose and variable-dose regimens is essential for assessing the practical benefit of the proposed optimization framework.

Such a comparison is explicitly included in the manuscript through the analysis of three progressively less constrained dosing strategies presented in the Results section (Figs. 4–7): (i) fixed-dose regimens, (ii) variable-dose regimens with fixed liberation rate, and (iii) fully optimized regimens with both dose magnitudes and liberation rates as decision variables.

First, conventional fixed-dose therapy, in which identical doses are administered at each dosing event, is evaluated. The results demonstrate an inherent limitation of such regimens: achieving a faster therapeutic onset requires increasing the effective input intensity (e.g., via higher liberation rate), which in turn leads to increased fluctuations and overshoot in the pharmacodynamic response. This illustrates a fundamental trade-off between transient performance (speed of onset) and steady-state behavior that cannot be resolved within the fixed-dose constraint.

The manuscript then considers a variable-dose regimen with a fixed liberation rate. In this case, allowing non-uniform dose magnitudes significantly improves transient performance, reducing the time required to reach the desired therapeutic effect from approximately 20–30 hours to about 7 hours. However, residual oscillations and overshoot remain, indicating that dose optimization alone is insufficient to fully shape the system dynamics.

Finally, the fully optimized strategy, in which both dose magnitudes and liberation rates are optimized, achieves further improvement. The desired therapeutic effect is reached within approximately 6 hours, while fluctuations are reduced by approximately a factor of four compared to the fixed-dose case. This demonstrates that simultaneous optimization of input magnitude and delivery kinetics provides additional degrees of freedom for shaping both transient and steady-state response.

Importantly, these results should be interpreted in a structural sense. Fixed-dose regimens correspond to a highly constrained subset of the admissible dosing strategies, whereas variable-dose regimens relax this restriction and allow the optimization to exploit the full dynamic response of the PK–PD system. The observed performance improvement is therefore not incidental, but reflects the fundamental limitation imposed by the fixed-dose constraint.

To improve clarity, we have revised the manuscript to explicitly summarize these quantitative differences and to highlight the underlying trade-offs between dosing constraints and achievable therapeutic performance.

The comparison between Figs. 4–7 highlights the impact of progressively relaxing constraints on the dosing strategy. In the fixed-dose case (Figs. 4–5), where identical doses are administered at each dosing event, the system exhibits a clear limitation: achieving faster therapeutic onset requires increasing the effective input intensity, which leads to larger fluctuations and overshoot in the pharmacodynamic response. As a result, the time required to reach the desired therapeutic effect remains on the order of 20–30 hours, depending on the chosen liberation rate, and is accompanied by significant steady-state oscillations.

Allowing variability in dose magnitudes (Fig. 6) substantially improves transient performance. In this case, the desired therapeutic effect is reached within approximately 7 hours, representing a reduction in onset time by more than a factor of three compared to fixed-dose regimens. This improvement arises because non-uniform dosing enables an initial loading phase followed by smaller maintenance doses, thereby partially decoupling transient and steady-state behavior. However, fluctuations and overshoot remain relatively pronounced, indicating that dose optimization alone does not fully resolve the underlying dynamic trade-offs.

The fully optimized strategy (Fig. 7), in which both dose magnitudes and liberation rates are optimized, provides the best overall performance. The target therapeutic effect is reached within approximately 6 hours, while fluctuations are reduced from approximately ±0.05 in the fixed-dose case to approximately ±0.012. This corresponds to both faster convergence and significantly improved tracking of the desired pharmacodynamic profile.

These results demonstrate that fixed-dose regimens represent a strongly constrained subset of admissible dosing strategies, which inherently limits achievable performance. Relaxing this constraint by allowing variable dose magnitudes introduces additional degrees of freedom that can be exploited to improve transient response, while further optimization of liberation rate enables simultaneous shaping of both transient and steady-state dynamics. Consequently, the proposed framework provides a systematic means of quantifying and exploiting these additional degrees of freedom, revealing performance improvements that are not attainable under conventional fixed-dose assumptions.

Reviewer 2:

Concern:

While the mathematical derivation is clear, the core problem of optimizing dosing regimens to achieve a desired therapeutic profile is already well-established and broadly solved in the existing literature. For example, the study by Bachmann et al. (OptiDose: Computing the Individualized Optimal Drug Dosing Regimen Using Optimal Control. J Optim Theory Appl 189, 46–65, 2021; https://doi.org/10.1007/s10957-021-01819-w) already addresses this optimal control challenge in a much more general and comprehensive manner. In contrast, this manuscript focuses on a very specific and simplified scenario restricted to linear pharmacokinetics and static Hill-function pharmacodynamics, completely neglecting the underlying disease dynamics. Furthermore, since the proposed optimization task is performed offline rather than in real-time, the computational burden is minimal, meaning the problem can be easily handled by any standard off-the-shelf numerical optimizer. Consequently, because the problem is not computationally difficult and the approach lacks broader applicability, the explicitly derived sensitivity formulas and Jacobians offer limited new scientific value or methodological advancement to the field.

Response:

We thank the reviewer for the insightful and highly relevant comment, and for highlighting the work by Bachmann et al. (OptiDose). We fully acknowledge that optimal control approaches to dosing regimen design are well-established, and that OptiDose represents a significant, general-purpose contribution in this area. We have revised the manuscript to explicitly acknowledge this work and clarify the distinction between the two approaches.

While both OptiDose and the present work formulate dosing as an optimization problem over dynamical PK–PD systems, there are key differences in problem formulation, decision variables, and exploitation of system structure:

1. Problem Scope and Model Structure
   OptiDose addresses a broad class of nonlinear PK–PD and disease models using a adjoint-based optimal control framework. In contrast, our work focuses on a structurally specialized but clinically relevant scenario: repeated drug administration where the pharmacokinetics can be represented by linear compartmental systems driven by impulsive inputs, coupled with nonlinear static Hill-type pharmacodynamics. This structural specialization is deliberate and enables a fundamentally different methodological approach.

2. Exploitation of Linearity and Superposition
   The linearity of the pharmacokinetic subsystem allows repeated dosing to be represented via superposition, which we explicitly exploit to reformulate the optimization problem as a structured nonlinear least-squares problem with analytically derived sensitivities. Unlike black-box or adjoint-based approaches, this yields exact, interpretable derivatives of the pharmacodynamic response with respect to both dose magnitudes and formulation-dependent parameters.

3. Joint Optimization of Dose and Formulation
   A central and novel aspect of our framework is the simultaneous optimization of both dose magnitudes and formulation-dependent pharmacokinetic parameters (liberation rates). To our knowledge, such joint optimization—allowing the drug release profile to be a design variable—has not been addressed in existing optimal control frameworks, including OptiDose. This enables emergent, clinically meaningful strategies, such as fast-loading doses followed by slower maintenance dosing, derived naturally from the optimization rather than imposed heuristically.

4. Analytical Sensitivities and Structured Optimization
   While finite-difference or adjoint-based methods could provide gradients in a black-box setting, they do not provide it analytically. Our analytical sensitivities:

   - Provide the exact Jacobian of the pharmacodynamic response with respect to both dose and formulation parameters.
   - Enable structured Gauss–Newton optimization, tailored to the least-squares formulation.
   - Offer direct interpretability of how dosing and release kinetics influence therapeutic response.
   - Scale naturally to higher-dimensional compartmental PK models without altering computational structure.

5. Scope and Extensibility
   Although our case studies use simplified PK–PD models and do not incorporate full nonlinear disease dynamics, the methodological contribution is independent of model complexity. The approach establishes a foundational framework for impulsive PK–PD optimization, which can be extended to higher-dimensional, nonlinear, or disease-integrated models.

In summary, the contribution of the present work lies not in introducing a new numerical solver for a known problem, but in reformulating dosing optimization in impulsive PK–PD systems with structured, analytically tractable sensitivities and extended decision variables, enabling insights and strategies that cannot be captured by general-purpose optimal control frameworks.

To better position the work within the existing literature, including the OptiDose framework, we have revised the Introduction and Conclusion sections accordingly and clarified the scope, novelty, and intended role of the proposed methodology.

Recent work [25] introduced the OptiDose framework, which determines optimal dosing schedules for general PK–PD models via classical adjoint-based optimal control. While highly general, these approaches limit analytical insight into how dosing and formulation parameters jointly influence the therapeutic trajectory. In contrast, this work focuses on repeated oral drug administration with linear compartmental pharmacokinetics and static Hill-type pharmacodynamics. This structure allows repeated doses to be represented via

superposition and enables analytical derivation of state–parameter sensitivities, providing direct interpretability and the ability to jointly optimize both dose magnitude and formulation-dependent release kinetics.

By treating liberation rate as an optimization variable alongside dose magnitude, the proposed framework simultaneously optimizes dosing and effective pharmacokinetic release characteristics, allowing the emergence of clinically meaningful strategies such as fast-acting loading doses followed by slower maintenance dosing.

25. *Bachmann F, Koch G, Pfister M, Szinnai G, Schropp J. OptiDose: Computing the Individualized Optimal Drug Dosing Regimen Using Optimal Control. J Optim Theory Appl. 2021;189: 46–65. doi:10.1007/s10957-021-01819-w*

We added the following to the Conclusion:

Compared to general-purpose optimal control approaches such as OptiDose, the present framework leverages structural properties of impulsive PK–PD systems. This allows reformulation of the dosing optimization problem as a structured nonlinear least-squares problem with analytically available Jacobians, which in turn facilitates a sensitivity-informed Gauss–Newton optimization. By including formulation-dependent parameters as decision variables, the framework identifies clinically meaningful dosing strategies that emerge naturally from the optimization process, rather than requiring heuristic specification.

Although the optimization problem can be solved offline using standard gradient-free solvers, the present work derives explicit state–parameter sensitivity equations to exploit structural properties of repeated impulsive dosing. Finite-difference approaches do not leverage the linearity of the PK subsystem and may suffer from numerical inaccuracies. The analytical sensitivities provide the exact Jacobian of the pharmacodynamic response with respect to both dose magnitudes and formulation-dependent liberation rates, enabling a structured Gauss–Newton optimization scheme and direct insight into the impact of dosing and release kinetics. Importantly, the approach scales naturally to higher-dimensional compartmental PK models while preserving structured Jacobian computation, making it a foundational tool for future extensions to more complex or disease-integrated models.

Concern:

The practical applicability of the proposed method is unclear. While the authors emphasize oral dosing in the introduction, prescribing an exact optimal dose is strictly limited by commercially available pill sizes (i.e., patients can only take predefined doses or perhaps halve the pills, restricting the input to discrete values). Furthermore, adjusting the liberation rate per dosing event is even more unrealistic due to manufacturing constraints, as pills are produced with fixed release profiles. The authors should clarify how this continuous optimization approach can be translated into real-world clinical practice.

Response:

We thank the reviewer for raising the important issue of practical applicability. We fully agree that translating continuous, model-based dosing strategies into clinical practice requires careful consideration of real-world constraints such as discrete pill sizes and fixed release profiles.

1. Continuous Optimization as a Methodological Step
   The continuous formulation used in this study, where decision variables are real-valued, represents a standard first step in optimal design problems. It allows the intrinsic structure of the optimal therapy—e.g., timing, relative magnitudes, and release kinetics—to be identified without introducing discrete constraints that could obscure the fundamental patterns. From a methodological standpoint, discrete or mixed-integer constraints (corresponding to fixed pill strengths or allowable release profiles) can be introduced as a subsequent refinement. Importantly, the PK–PD model, sensitivity analysis, and structured optimization framework remain fully applicable; only the feasible set of decision variables is restricted.

2. Practical Implementation of Continuous Dose Targets
   Optimized dose values should therefore be interpreted as ideal target doses, which can be mapped onto realizable values using standard pharmaceutical strategies:

   - Rounding or combination of available tablet strengths to approximate the target dose.
   - Tablet splitting, where clinically appropriate, for finer granularity.
   - Pre-packaged treatment kits or sequential blister packs, which allow patients to follow variable dosing regimens safely, using a small set of base tablet strengths.

This approach preserves the clinical intent of the optimization while remaining compatible with commercially available formulations.

3. Interpretation and Realization of Liberation Rate Optimization
   While conventional tablets have fixed release kinetics, modern pharmaceutical technologies enable precise control of drug dissolution rates. Optimized liberation rates in our framework should be viewed as design targets for formulation, guiding the selection or engineering of appropriate release technologies rather than implying that every individual tablet must be uniquely manufactured. Examples include:

   - Controlled-release matrix tablets or multilayer tablets.
   - Coated pellets and osmotic delivery systems.
   - Microencapsulation or polymer-based formulations.

Treatment regimens can therefore combine a small number of formulations with distinct release profiles, such as an initial fast-release loading dose followed by slower sustained-release maintenance doses, to closely approximate the optimized profile.

4. Clinical and Manufacturing Feasibility
   Importantly, variable dose magnitudes across dosing events, as produced by the framework, can be implemented safely and practically via sequential blister packs, pre-packaged treatment kits, or modular combination of tablets. This strategy:
   - Reduces risk of dosing errors.
   - Is scalable to large patient populations.
   - Leverages a limited set of base tablet strengths or formulations, minimizing manufacturing complexity.

We have revised the manuscript to clarify how the proposed optimization framework can be implemented under realistic pharmaceutical and clinical constraints.

Pharmaceutical manufacturing imposes inherent constraints on the number of available dose strengths. The optimized dose values obtained in this study should therefore be interpreted as ideal target doses, rather than requiring each dose to correspond to a uniquely manufactured product. In practice, these targets can be implemented by rounding to the nearest commercially available strength, combining tablets of different strengths, or employing tablet splitting where clinically appropriate.

From a methodological perspective, these practical constraints correspond to discrete or mixed-integer restrictions on the optimization variables, limiting admissible doses to a predefined set determined by available pharmaceutical products. Importantly, the underlying PK–PD optimization framework remains unchanged; only the feasible set of decision variables is modified. In this context, continuous optimization serves as an initial design step, revealing the intrinsic structure of optimal therapy, which can subsequently be mapped to implementable dosing regimens without altering the core methodology.

Optimized dosing sequences may involve variable doses across multiple administrations. Such regimens can be implemented safely and efficiently using pre-packaged treatment kits or sequential blister packs, where tablets are arranged and numbered according to the prescribed schedule. Large-scale manufacturing is feasible because the regimen can be realized using a small set of base tablet strengths, combined appropriately to achieve the desired dosing sequence, or through minor controlled variations in tablet mass.

A similar rationale applies to the optimization of drug liberation rates. In the present framework, the liberation rate functions as a design target for formulation development, rather than implying that every individual tablet must possess a uniquely engineered release profile. Modern drug delivery technologies—including controlled-release matrices, polymer-coated pellets, multilayer tablets, osmotic delivery systems, and microencapsulation—allow precise manipulation of drug dissolution and release kinetics. Consequently, treatment regimens can incorporate a small number of formulations with distinct release characteristics, such as an initial fast-release tablet followed by sustained-release maintenance doses.

Taken together, the proposed methodology provides a systematic, quantitative framework for guiding both clinical dose design and pharmaceutical formulation development, while remaining fully compatible with large-scale manufacturing and real-world therapeutic practice.

Concern:

In lines 104-108, the authors state: "Impulsive control theory has also been applied... These methods, however, are inherently conservative and typically converge to uniform dosing patterns." Interestingly, the results of this manuscript ultimately converge to a very similar uniform pattern.

In lines 581-583, the authors conclude: "The most important conclusion is that the optimal therapy constitutes of a larger leading dose... followed by smaller maintenance doses with much slower liberation, which is actually in compliance with the basic intuition."

Thus, the final outcome of the proposed method does not significantly differ from the standard approaches the authors criticize.

Response:

We thank the reviewer for this insightful observation. While the optimized dosing pattern—comprising a larger initial dose followed by smaller maintenance doses—may superficially resemble classical uniform or leading-dose strategies, it is important to emphasize that this pattern is an emergent property of the optimization procedure, rather than being assumed a priori or heuristically imposed.

The proposed method explicitly and rigorously optimizes both dose magnitudes and formulation-dependent release kinetics within a nonlinear PK–PD model.

Although the resulting dosing sequence may converge toward a pseudo-steady-state uniform pattern, the exact per-dose amounts and release rates are quantitatively derived from the structured optimization framework, rather than chosen intuitively. This ensures that each dose and release profile is mechanistically justified, patient-specific, and optimally tuned to achieve the desired therapeutic trajectory.

The emergent similarity to intuitive strategies (e.g., loading dose followed by maintenance dosing, fast liberation rate at the beginning, and slower liberation rate during the maintenance) demonstrates the validity of clinical intuition, but the key distinction is that our method provides a rigorous, predictive, and flexible framework capable of:

- Exploiting nonlinear PD effects.
- Accounting for compartmental PK dynamics.
- Jointly optimizing multiple design variables, including release kinetics.

Therefore, our approach goes beyond the conservative uniform solutions of classical impulsive control, providing quantitative, patient-specific guidance for dosing schedules.

We modified the manuscript as follows:

An important conclusion is that the optimal therapy consists of a larger leading dose with fast liberation followed by smaller maintenance doses with slower liberation. Crucially, this pattern emerges naturally from the joint optimization of both dose magnitude and formulation-dependent release kinetics under the nonlinear PK–PD model, rather than being imposed a priori or heuristically.

……….

Unlike classical impulsive control approaches that typically assume fixed doses or uniform release profiles, our method simultaneously optimizes both the per-dose amount and the release kinetics.

……….

Although the resulting dosing pattern may superficially resemble standard loading-dose or uniform regimens, the similarity is quantitative rather than assumed. Each dose and release profile is the outcome of a structured optimization procedure that explicitly accounts for nonlinear pharmacodynamics and compartmental pharmacokinetics. This approach provides mechanistically justified, flexible, and patient-specific dosing schedules, going beyond the conservative uniform solutions typically produced by classical impulsive control methods.

Concern:

Lines 181-182: "The Dirac impulse defined in Eq (2) provides an appropriate abstraction for oral drug dosing, as such therapies consist of intermittent, event-driven interventions." The use of the term "event-driven" is misleading here. According to Eq (2), drug administration occurs at strictly fixed, equidistant time intervals (T_D), making it time-triggered rather than event-driven. Moreover, assuming perfectly equidistant dosing intervals is clinically unrealistic, as patient compliance rarely allows for taking pills at the exact prescribed minute. The authors should justify why fixing the dosing intervals is necessary for their algorithm.

Response:

We thank the reviewer for raising this important point. We agree that the original term “event-driven” could be misleading, and we have revised the manuscript to consistently use “discrete, intermittent dosing” to describe the Dirac impulse abstraction.

In our framework, the Dirac impulses represent discrete, intermittent oral dosing events. Assuming equidistant dosing intervals is a deliberate methodological choice that provides mathematical tractability. Specifically:

- It allows each individual dose response to be represented as a vector of fixed length and known start time.
- This enables superposition and vector stacking, which are essential for efficiently constructing the full system response and computing the analytical sensitivities required for structured nonlinear least-squares optimization.

- Without this assumption, the optimization would require non-equidistant convolutions or complex time-shifting operators, significantly complicating the derivation of exact Jacobians and the structured Gauss–Newton solver.

While perfect adherence to fixed dosing times is not realistic, minor timing deviations are typically clinically tolerable and insignificant, especially for drugs with moderate to long half-lives or gradual pharmacodynamic responses. However, the framework is compatible with sensitivity analysis, which can quantify the impact of small timing errors and ensure that optimal therapeutic outcomes are maintained. In this sense, the fixed interval assumption is a practical modeling abstraction that preserves both analytical clarity and clinical relevance.

The use of fixed, equidistant dosing intervals is a **deliberate trade-off**: it enables rigorous derivation of structured sensitivities, efficient optimization, and analytical clarity, while preserving clinical relevance for the majority of oral therapies.

The current formulation serves as a foundational building block. Extensions to flexible dosing times are a natural and important next step. Extending the framework to optimize non-equidistant or adherence-informed dosing schedules is fully feasible and represents a promising avenue for future work.

Accordingly, we have replaced the term “event-driven” with “discrete, intermittent dosing” throughout the manuscript and explicitly clarified the rationale for fixed dosing intervals in the Methods section.

The Dirac impulse defined in Eq (2) provides a convenient abstraction for oral drug dosing, representing discrete, intermittent administration. In the present study, these doses are assumed to occur at fixed, equidistant intervals. This assumption is necessary for mathematical tractability: it ensures that the response to each individual dose can be represented as a vector of known length and starting time, which allows stacking and superposition of the response vectors required for the nonlinear least squares problem. While clinically, exact adherence to dosing times may vary, small deviations from prescribed times generally have limited effect on therapeutic outcomes for drugs with moderate half-lives and gradual pharmacodynamic [3,4].

We added the following paragraph to the conclusion:

Future work will focus on optimization of individual dosing times, abandoning the assumption of fixed intervals, to achieve further improvements in therapeutic performance while maintaining analytical tractability. Extending the framework to non-equidistant or adherence-informed dosing schedules is feasible and represents an important direction for clinical translation and adaptive dosing strategies.

Concern:

Lines 182-185: "Each administration introduces the prescribed dose into the gastrointestinal compartment over a time interval that is negligible compared to the characteristic time scales of pharmacokinetic processes." The authors should be more precise mathematically. Do they mean that the duration of drug intake is negligible compared to the smallest time constant (or half-life) of the PK model? A clearer mathematical or physiological definition of these "characteristic time scales" would be helpful.

Response:

We thank the reviewer for pointing out the need for a more precise definition of "characteristic time scales" in the context of our impulsive dosing assumption. In our manuscript, the statement refers to the classical approximation used in pharmacokinetics where the drug input is considered instantaneous relative to the dynamics of drug distribution and elimination. Mathematically, this means that the duration of drug administration, denoted Δt_{dose}, is much smaller than the smallest time constant of the PK system. Equivalently, for a standard one- or multi-compartment PK model with first-order elimination rates k_i, the characteristic time scales are simply $1/k_i$. The impulsive approximation is valid when:

$$\Delta t_{\text{dose}} \ll \min_i \frac{1}{k_i}.$$

From a physiological standpoint, this ensures that the drug is effectively "available" in the gastrointestinal compartment before significant redistribution or elimination occurs, which justifies treating each administration as an instantaneous impulse for the purpose of the PK–PD simulation and optimization.

We revised the manuscript to include this explicit definition, replacing "characteristic time scales:

Each administration introduces the prescribed dose into the gastrointestinal compartment over a small time interval Δt_{dose}that is negligible compared to the characteristic pharmacokinetic time scales, i.e., $\Delta t_{dose} \ll min_i\ 1/k_i$, where $k_i$are the first-order elimination or transfer rates of the compartments in the PK model. This ensures that each dose can be accurately approximated as an instantaneous (impulsive) input for the purpose of PK–PD modeling and optimization. From a physiological standpoint, this ensures that the drug is effectively "available" in the gastrointestinal compartment before significant redistribution or elimination occurs.

Concern:

The authors should explicitly state that the parameters θ^i remain constant between the consecutive dosing injections (discrete events).

Response:

We understand and accept the concern. We added this explicit statement in the manuscript:

The parameter vector θ^i of p adjustable parameters to be optimized includes the dose size and chosen drug characteristics, which can be technologically influenced/modified. The parameters θ^i remain constant between the consecutive dosing injections (discrete events).

Concern:

The variable j in Eq (4) is undefined. Does it represent the number of administered injections up to time t? This needs to be explicitly defined. Furthermore, j is reused as a parameter index in Eq (14), which causes mathematical ambiguity and should be corrected.

Response:

We understand and accept the concern.

The meaning of j is exactly as you stated. Furthermore, we disambiguated the meaning of j reused in Eq (14) as:

The overall effect of multiple dose administration can be expressed in terms of the superposition principle applied to Eq (1) as

$$\dot{x}(t,\boldsymbol{\theta}) = \sum_{i=1}^{q}\left[A(\theta^i)x^i\left(t,\theta^i\right) + B(\theta^i)u^i(t)\right] \quad (4) .$$

where q is the number of administered doses up to time t.

The solution of the system state for no previous treatment, represented by $x(0,\boldsymbol{\theta}) = \mathbf{0}$, then gets [40,41]

$$x(t,\boldsymbol{\theta}) = \sum_{i=1}^{q} x^i\left(t,\theta^i\right) = \sum_{i=1}^{q} e^{A(\theta^i)(t-(i-1)T_D)}B(\theta^i) \text{ (5),}$$

Concern:

Hyphens and dashes are used incorrectly or interchangeably in several places, for example in lines 283 and 427. Please review the punctuation for standard academic formatting.

Response:

The punctuation has been carefully reviewed and corrected throughout the manuscript. In particular, the usage of hyphens (-), en dashes (–), and em dashes (—) has been standardized according to conventional academic formatting.

Concern:

Definite articles are missing in several places throughout the manuscript. For instance, in line 139: "One of central" should be "One of the central"; and in line 249: "Consider vector" should be "Consider the vector". A thorough proofreading is recommended.

Response:

Thank you for the careful observation. The manuscript has been thoroughly proofread with particular attention to the use of definite articles. The entire text has been carefully reviewed to ensure proper and consistent use of articles throughout the manuscript.

Concern:

Lines 466-469: "The logistic form was selected because it naturally reflects how clinicians conceptualize an ideal therapeutic course..." This is a strong clinical claim. Please provide appropriate references from the medical or pharmacological literature to support the statement that clinicians conceptualize the ideal therapeutic course in this specific mathematical form.

Response:

We thank the reviewer for pointing out that the original wording may overstate the clinical interpretation of the logistic function. We agree that clinicians do not explicitly conceptualize treatment response in terms of a specific mathematical function. The intention of the statement was rather to highlight that the logistic profile reflects several qualitative properties commonly observed in pharmacological response dynamics.

To avoid overstating the clinical interpretation, the manuscript has been revised to clarify that the logistic reference trajectory was chosen because it reflects general pharmacodynamic response characteristics, rather than representing a literal clinical prescription model. Appropriate references to the pharmacological literature describing sigmoidal dose–response relationships have been added.

We modified the manuscript as follows:

The logistic reference trajectory reflects qualitative features commonly desired in pharmacotherapy, namely a limited initial response during drug accumulation, a phase of increasing therapeutic effect as drug exposure approaches effective levels, and a subsequent stabilization of the effect within a desired therapeutic range. Such behavior corresponds to the general objective of dosing regimen design, which aims to achieve therapeutic effect within a reasonable time and maintain it at a stable level while avoiding excessive fluctuations [3,4].

3. *Rowland M, Tozer TN. Clinical Pharmacokinetics and Pharmacodynamics: Concepts and Applications. 4th ed. Wolters Kluwer Health/Lippincott William & Wilkins; 2011.*

4. *Bauer LA. Clinical Pharmacokinetics and Pharmacodynamics. In: DiPiro JT, Yee GC, Posey LM, Haines ST, Nolin TD, Ellingrod V, editors. Pharmacotherapy: A Pathophysiologic Approach, 11e. New York, NY: McGraw-Hill Education; 2020. Available: accesspharmacy.mhmedical.com/content.aspx?aid=1182422557*

Editor:

Concern:

Positioning within the literature:

Both reviewers suggest that the manuscript would benefit from a clearer discussion of how your framework compares with existing PK/PD dose optimization methods. In particular, please consider including recent references suggested by Reviewer 1 (e.g., Chua & Tam, 2022; Chotsiri et al., 2025) and any other relevant studies that situate your approach within the broader PK/PD and optimal control literature.

Response:

We thank the editor for this helpful suggestion. We agree that the manuscript benefits from a clearer positioning within the existing literature on PK–PD–based dose optimization.

To address this comment, we revised the Introduction and Discussion sections to better situate our work relative to established approaches. In particular, we incorporated the recent references suggested by Reviewer 1 and expanded the discussion of commonly used PK–PD dose optimization strategies.

The revised manuscript now discusses classical PK–PD index–based approaches, as well as modern model-informed dosing frameworks based on population simulations and exposure matching. We also expanded the discussion of optimal-control-based dosing frameworks, including the OptiDose approach, which formulates dose optimization as a dynamical control problem for general PK–PD models.

The manuscript now clarifies that the present work provides a complementary formulation in which repeated oral dosing is modeled as impulsive inputs to a linear compartmental pharmacokinetic system coupled with a nonlinear pharmacodynamic model. Within this structure, the proposed framework simultaneously optimizes dose magnitude and formulation-dependent liberation rate, and exploits analytical state–parameter sensitivity equations to construct a structured Gauss–Newton optimization problem.

These revisions more clearly position the proposed approach within the broader PK–PD and optimal-control literature and highlight its methodological contribution.

Concern:

Practical applicability

Reviewer 1 notes that your proposed optimized dosing strategy involves variable doses at each administration, which may be difficult to implement in real-world clinical practice. Both reviewers highlight that inter-patient variability, patient compliance, and manufacturing constraints (fixed dose strengths and release profiles) are important factors. Please clarify the scope of your approach and discuss how these practical constraints may influence the application or interpretation of your results.

Response:

We agree that practical considerations such as inter-patient variability, patient adherence, and pharmaceutical manufacturing constraints are essential when translating model-based dosing strategies into clinical practice. In the revised manuscript, we have clarified the scope of the proposed framework and expanded the discussion of these aspects in the Conclusion.

First, we emphasize that the primary objective of the present work is to develop a methodological framework for PK–PD–based optimization of repeated drug dosing, rather than to propose a ready-to-implement clinical dosing protocol. The optimization is therefore formulated in a continuous setting to reveal the intrinsic structure of the optimal therapy. The resulting dose values should be interpreted as optimal target doses, which can subsequently be mapped to clinically realizable values.

Second, we clarified how manufacturing constraints can be incorporated. In practice, tablets are available only in a limited set of strengths and release profiles. From an optimization perspective, this corresponds to restricting the decision variables to a discrete set of admissible values, leading to a mixed-integer or discrete optimization formulation. Importantly, the PK–PD model and the sensitivity-based optimization framework remain unchanged; only the feasible set of dose values is modified. In clinical practice, optimized dose sequences can be approximated using combinations of available tablet strengths, tablet splitting where appropriate, or pre-packaged sequential blister packs that guide patients through variable regimens.

Third, we clarified the interpretation of the liberation rate as a formulation design parameter rather than implying that every individual dose must have a uniquely manufactured release profile. Modern drug delivery technologies (e.g., controlled-release matrices, coated pellets, multilayer tablets, or osmotic systems) allow the creation of formulations with different release characteristics. In practice, treatment regimens may combine a small number of formulations, for example a fast-release loading dose followed by sustained-release maintenance doses.

Finally, we expanded the discussion of inter-patient variability and adherence. In the present study, PK–PD parameters were treated as fixed in order to focus on the methodological development. However, the proposed framework can be applied to individualized PK–PD models obtained through therapeutic drug monitoring or population PK–PD modeling.

These clarifications have been added to the manuscript to better explain how the proposed optimization framework can guide both dose design and formulation development, while remaining compatible with real-world therapeutic and pharmaceutical constraints.

Concern:

Demonstration of advantage

Reviewer 1 requests a comparison between the proposed optimized dosing regimen and simpler, more standard strategies (e.g., constant-dose regimens) to illustrate the potential practical advantage of your approach.

Response:

We agree that demonstrating the advantage of the proposed optimization approach relative to simpler dosing strategies is important for illustrating its practical relevance.

This comparison is included in the manuscript through the analysis of fixed-dose regimens and optimized variable-dose regimens, presented in the Results section (Figs. 4–7). In particular, the manuscript first evaluates a conventional fixed-dose therapy in which the same dose is administered at every dosing event. The resulting therapeutic profiles show a relatively slow onset of therapy (approximately 20–30 hours to reach the desired effect) together with noticeable oscillations around the steady-state response.

The manuscript then considers a scenario where only the dose magnitudes are optimized while the liberation rate remains fixed. This variable-dose regimen substantially accelerates the therapeutic onset, reaching the desired effect within approximately 7 hours, although fluctuations remain relatively pronounced.

Finally, the fully optimized therapy, in which both dose magnitude and liberation rate are optimized, further improves performance by achieving the desired therapeutic response within approximately 6 hours while significantly reducing oscillations around the target effect.

To make this comparison clearer, we revised the Results and Discussion sections to explicitly summarize the quantitative differences between fixed-dose and optimized regimens. These results demonstrate that relaxing the fixed-dose constraint allows significantly faster therapeutic onset and improved tracking of the desired pharmacodynamic response trajectory.

Concern:

Mathematical and technical clarifications

Reviewer 2 raised a number of technical points regarding notation, definitions, and the presentation of certain assumptions (e.g., the definition of variables, the constancy of parameters between doses, and characteristic time scales). Please carefully review the manuscript and clarify these aspects. Address the specific points noted in Reviewer 2's comments to ensure mathematical rigor and clarity.

Response:

In response to Reviewer 2's comments, the manuscript has been carefully reviewed and revised to address the noted issues related to notation, definitions, and the presentation of modeling assumptions.

Several specific clarifications have been introduced in the revised manuscript. First, ambiguous or undefined variables have been explicitly defined. In particular, the variable $q$is now introduced as the number of administered doses up to time $t$in the formulation of the superposition principle for repeated dosing. In addition, notation that was previously reused in different contexts has been disambiguated to avoid potential confusion.

Second, we explicitly clarified the assumption that the parameter vector θ^i, which contains the adjustable parameters associated with the i-th dose (including dose magnitude and formulation-dependent characteristics), remains constant between consecutive dosing events.

Third, the manuscript now provides a more precise definition of the pharmacokinetic time scales used to justify the impulsive dosing approximation. Specifically, the duration of drug administration $\Delta t_{\text{dose}}$is assumed to be much smaller than the smallest characteristic time constant of the pharmacokinetic system, i.e.,
$\Delta t_{\text{dose}} \ll \min_i (1/k_i)$, where $k_i$are the first-order transfer or elimination rates of the compartments.

In addition, the terminology describing dosing events has been revised to improve accuracy. The phrase “event-driven dosing” has been replaced with “discrete, intermittent dosing,” and the assumption of equidistant dosing intervals has been explicitly explained as a modeling simplification that facilitates the derivation of the superposition-based formulation.

Concern:

Editorial improvements

A thorough proofreading of the manuscript is recommended. Additionally, Reviewer 2 suggested providing references to support clinical claims, such as statements about the logistic form reflecting clinicians’ conceptualization of therapeutic courses.

Response:

In response, the manuscript has undergone a thorough proofreading to improve clarity, correct grammar, and ensure consistent use of punctuation, including the standardized use of hyphens, en dashes, and em dashes throughout the text. Definite articles and other minor language issues were carefully reviewed and corrected.

In addition, clinical claims have been clarified and supported with appropriate references. For example, the statement regarding the logistic reference trajectory has been revised to indicate that it was chosen to reflect general pharmacodynamic response characteristics—such as gradual onset, progressive effect accumulation, and stabilization within a desired therapeutic range—rather than implying that clinicians explicitly conceptualize treatment response in this form. Relevant pharmacological references have been added to support this clarification:

- Rowland M, Tozer TN. *Clinical Pharmacokinetics and Pharmacodynamics: Concepts and Applications*, 4th ed. Wolters Kluwer Health; 2011.
- Bauer LA. *Clinical Pharmacokinetics and Pharmacodynamics*. In: DiPiro JT, Yee GC, Posey LM, Haines ST, Nolin TD, Ellingrod V, editors. *Pharmacotherapy: A Pathophysiologic Approach*, 11e. New York, NY: McGraw-Hill Education; 2020.
