# Supplementary material for: Optimal design of dose and drug pharmacokinetic characteristics to achieve the desired pharmacodynamic profile in repeated drug dosing
Source: PLoS One. 2026 Jul 22;21(7):e0354029. doi: 10.1371/journal.pone.0354029 (PMC13390843; doi:10.1371/journal.pone.0354029)
Supplement: Attachment — Submitted filename: Review response 2.pdf [file pone.0354029.s003.pdf]

Revision notes on the manuscript PONE-D-26-06346R1 entitled Optimal design of dose and drug pharmacokinetic characteristics to achieve the desired pharmacodynamic profile in repeated drug dosing, submitted to PLOS One

Reviewer 3:

Concern:

Model dependence and identifiability: the method assumes an accurately identified PK–PD model, but the optimization relies on local sensitivities. There is no discussion of parameter identifiability, conditioning of the Jacobian, or robustness to model mismatch—critical issues in such inverse problems.
(Editor: Expand the discussion regarding the dependence of the proposed optimization framework on the accuracy of the underlying PK/PD model. In particular, discuss issues related to parameter identifiability, conditioning of the Jacobian, and sensitivity to model mismatch.)

Response:

We thank the reviewer and the editor for raising this important point. We fully agree that the proposed optimization framework is inherently model-dependent and that the quality of the resulting dosing strategy depends on the accuracy and identifiability of the underlying PK–PD model.

The present methodology formulates dose optimization as a structured nonlinear inverse problem in which the therapeutic effect trajectory is shaped through model-based optimization using sensitivity-derived Jacobians. Consequently, issues such as parameter identifiability, Jacobian conditioning, parameter correlation, and robustness to model mismatch are indeed fundamentally important.

To address this concern, the revised manuscript has been expanded substantially in several places.

First, we now explicitly clarify that the framework assumes the availability of a sufficiently informative and identifiable PK–PD model obtained through prior pharmacometric identification, therapeutic drug monitoring, or system identification procedures. The optimization methodology itself does not eliminate identifiability limitations intrinsic to the underlying PK–PD structure.

Second, the revised manuscript now discusses the conditioning of the Jacobian matrix used in the Gauss–Newton iterations. In particular, we emphasize that:

- the optimization variables were intentionally restricted to clinically modifiable parameters (dose magnitude and liberation rate),

- the number of optimized variables remains relatively low,
- and Tikhonov regularization was incorporated specifically to mitigate ill-conditioning and parameter correlation effects.

The regularization term stabilizes the inverse problem by penalizing large deviations from nominal treatment parameters and improving numerical conditioning of the approximate Hessian matrix.

Third, we now explicitly discuss robustness limitations arising from model mismatch and inter-patient variability. Since the optimization is performed offline using nominal PK–PD parameters, discrepancies between predicted and actual patient dynamics may degrade therapeutic performance.

We added the following section:

Model dependence, identifiability, and robustness considerations

The proposed framework is formulated as an open-loop nominal optimization problem and therefore its performance depends on the predictive accuracy of the underlying PK–PD model. Consequently, uncertainties in pharmacokinetic or pharmacodynamic parameters, structural model mismatch, inter-patient variability, adherence deviations, or unmodeled physiological disturbances may affect the resulting therapeutic response.

In particular, inaccuracies in elimination rates, absorption dynamics, receptor sensitivity, or concentration–effect relationships may alter both the transient onset and steady-state behavior of the optimized therapy. Since the present methodology performs optimization offline without feedback correction, such discrepancies may lead to deviations between the predicted and actual pharmacodynamic trajectories.

These limitations are intrinsic to deterministic open-loop optimization approaches relying on predicted system dynamics. Therefore, the present framework should primarily be interpreted as a nominal model-informed optimization methodology rather than a guaranteed robust control strategy.

From a mathematical standpoint, the present methodology constitutes a structured nonlinear inverse problem. As with all inverse problems, practical limitations may arise due to parameter non-identifiability, parameter correlation, insufficient excitation of the system dynamics, measurement noise, or structural model mismatch.

In particular, if multiple parameter combinations produce similar therapeutic effect trajectories, the Jacobian matrix of the optimization problem may become ill-conditioned or locally rank-deficient. Such situations reduce numerical robustness of the Gauss–Newton iterations and increase sensitivity to perturbations in model parameters or measured data.

Several structural aspects of the proposed framework partially mitigate these difficulties. First, the optimization variables were intentionally restricted to clinically and technologically modifiable parameters, namely dose magnitude and liberation rate. This substantially reduces the dimensionality of the optimization problem and limits parameter correlation. Second, the repeated impulsive excitation generated by multiple dosing events improves distinguishability of transient and steady-

state pharmacodynamic behavior. In particular, the liberation rate primarily influences the temporal shape and phase characteristics of the concentration trajectory, whereas the dose magnitude predominantly scales the amplitude of the response.

We expanded the description of regularization in the optimization problem:

The inclusion of the Tikhonov regularization term in Eq (24) is important not only from a therapeutic standpoint, but also from a numerical inverse-problem perspective. Besides penalizing excessively aggressive dosing strategies, the regularization improves conditioning of the approximate Hessian matrix $\left(\nabla \boldsymbol{E}(\boldsymbol{\theta}_k)\right)^T \nabla \boldsymbol{E}(\boldsymbol{\theta}_k) + \Lambda$ *and reduces sensitivity of the optimization to parameter correlations and local Jacobian degeneracies.*

We added the following acknowledgement:

It is important to emphasize that the proposed sensitivity analysis is local in nature. Consequently, the Jacobian matrices characterize only local parameter influence around the current operating point and parameter estimate.

For strongly nonlinear PK–PD systems or large parameter deviations, local sensitivities may not fully capture global parameter interactions or multimodal optimization structure. Nevertheless, for the considered class of compartmental PK–PD models operating within clinically relevant concentration ranges, local sensitivity information provides an efficient and computationally tractable approximation enabling structured Gauss–Newton optimization.

We also added explicit robustness limitation into discussion:

Since the optimization is performed offline using nominal PK–PD parameters, the resulting dosing strategies may degrade in performance when significant inter-patient variability, unmodeled physiological changes, adherence deviations, or structural model mismatch are present. Therefore, the proposed framework should presently be interpreted primarily as a model-informed nominal optimization methodology rather than a guaranteed robust control strategy.

Concern:

Structural assumptions: linearity of PK, static PD mapping, fixed dosing intervals, and impulsive approximation constrain the admissible dynamics; these assumptions limit generality and should be more explicitly framed as restrictions rather than general features.

(Editor: The framework relies on several simplifying assumptions, including: linear pharmacokinetics, static Hill-type pharmacodynamics, fixed dosing intervals, impulsive dosing approximation. These assumptions should be more clearly presented as methodological restrictions defining the scope of applicability of the proposed approach.)

Response:

We fully agree that the proposed framework relies on several structural assumptions. The primary objective of the present work was not to propose a universally general PK–PD framework, but rather to establish a mathematically structured and analytically tractable optimization methodology within repeated oral dosing.

The above assumptions were therefore adopted intentionally as methodological restrictions defining the scope of applicability of the current framework. Importantly, these assumptions are consistent with a large class of clinically relevant PK–PD models routinely used in pharmacometrics and control-oriented biomedical modeling.

In particular:

- Linear compartmental PK models remain one of the most widely used representations in pharmacokinetics due to their interpretability, identifiability, and ability to approximate many drugs operating in non-saturating concentration ranges.
- The static Hill-type PD mapping was selected because it captures the dominant nonlinear concentration–effect relationship while preserving analytical differentiability required for sensitivity-based Jacobian construction and Gauss–Newton optimization.
- Fixed dosing intervals were introduced primarily to preserve the stacked block structure of the nonlinear least-squares formulation and enable direct superposition of repeated impulsive responses.
- The impulsive dosing approximation reflects the inherently intermittent nature of oral administration and is valid whenever the administration time is negligible relative to the dominant pharmacokinetic time constants.

The manuscript has been revised to more explicitly present these assumptions as structural restrictions.

We added the following to the introduction:

Despite its generality within the class of compartmental PK–PD models, the present framework relies on several structural assumptions that define its current scope of applicability. Specifically, the methodology assumes:

- *linear compartmental pharmacokinetics,*
- *static concentration–effect pharmacodynamics represented by a Hill-type function,*
- *fixed equidistant dosing intervals,*
- *impulsive approximation of dose administration.*

These assumptions were intentionally adopted to obtain a mathematically tractable optimization framework permitting analytical sensitivity derivation, structured Jacobian computation, and efficient Gauss–Newton optimization. Consequently, the present work should be interpreted as establishing a structured methodological foundation for optimization of repeated oral dosing rather than as a universally general PK–PD framework applicable to all drug classes and physiological conditions.

We added the following to the Pharmacokinetic model section:

The assumption of linear pharmacokinetics constitutes an important modeling restriction of the present framework. The methodology is therefore primarily applicable to drugs operating within concentration ranges where absorption, distribution, and elimination processes remain approximately first-order and non-saturating.

This excludes drugs exhibiting strong nonlinearities such as saturable metabolism, concentration-dependent clearance, transporter saturation, autoinduction, target-mediated drug disposition, or nonlinear protein binding. Nevertheless, linear compartmental models remain highly relevant in practical pharmacokinetics because many therapeutics operate predominantly within approximately linear exposure ranges under standard dosing conditions.

We added the following to the Pharmacodynamic model section

Consequently, the framework does not presently capture dynamic pharmacodynamic phenomena such as receptor turnover, delayed signal transduction, tolerance development, hysteresis, indirect response mechanisms, or adaptive biological feedback. The static formulation was selected to isolate the influence of pharmacokinetic shaping on the therapeutic effect trajectory while preserving analytical differentiability.

We modified the following justification of impulsive approximation:

Consequently, the framework is primarily intended for intermittent oral or bolus-like administration and is not directly applicable to prolonged infusion therapies or continuously varying administration profiles.

We modified the following justification of fixed dosing intervals

In the present study, these doses are assumed to occur at fixed equidistant intervals. This assumption is necessary for mathematical tractability: it ensures that the response to each individual dose can be represented as a vector of known length and starting time, which allows stacking and superposition of the response vectors required for the nonlinear least squares problem. While exact adherence to dosing times may vary, small deviations from prescribed times generally have a limited effect on therapeutic outcomes for drugs with moderate half-lives and gradual pharmacodynamic [3,4]. Allowing fully variable dosing times would transform the problem into a more general hybrid optimal control problem with variable event timing, substantially increasing computational complexity and altering the structure of the sensitivity equations.

Concern:

Robustness and uncertainty: the framework is fully open-loop; no analysis is provided on how errors in PK/PD parameters affect the optimal solution.

(Editor: Include a discussion on robustness with respect to uncertainty in PK/PD parameters and modeling errors. Even if a complete robustness analysis is beyond the scope of the present work, the limitations of the current open-loop formulation should be acknowledged explicitly.)

Response:

We fully agree that the present framework constitutes an open-loop model-based optimization methodology and that no formal robustness analysis with respect to PK–PD parameter uncertainty or structural model mismatch was included in the current manuscript.

The primary objective of the present work was to establish a mathematically structured deterministic optimization framework for repeated oral dosing based on impulsive PK–PD dynamics. Consequently, the optimization was formulated using nominal PK–PD parameters.

We agree that uncertainties in pharmacokinetic and pharmacodynamic parameters will affect the resulting optimal dosing strategy. In particular, inaccuracies in elimination rates, absorption dynamics, receptor sensitivity, or concentration–effect relationships may alter both transient therapeutic onset and steady-state pharmacodynamic behavior. Since the optimization is performed offline without feedback correction, such discrepancies may lead to deviations between predicted and actual therapeutic responses.

To address this concern, the revised manuscript now explicitly acknowledges the limitations of the current open-loop formulation and expands the discussion regarding robustness, uncertainty, and model mismatch. In particular, we clarify that the present methodology should be interpreted as a nominal model-informed optimization framework rather than a guaranteed robust control strategy.

We added a dedicated subsection to the Discussion

The proposed framework is formulated as an open-loop nominal optimization problem and therefore its performance depends on the predictive accuracy of the underlying PK–PD model. Consequently, uncertainties in pharmacokinetic or pharmacodynamic parameters, structural model mismatch, inter-patient variability, adherence deviations, or unmodeled physiological disturbances may affect the resulting therapeutic response.

In particular, inaccuracies in elimination rates, absorption dynamics, receptor sensitivity, or concentration–effect relationships may alter both the transient onset and steady-state behavior of the optimized therapy. Since the present methodology performs optimization offline without feedback correction, such discrepancies may lead to deviations between the predicted and actual pharmacodynamic trajectories.

These limitations are intrinsic to deterministic open-loop optimization approaches relying on predicted system dynamics. Therefore, the present framework should primarily be interpreted as a nominal model-informed optimization methodology rather than a guaranteed robust control strategy.

Nevertheless, the present deterministic formulation constitutes an essential prerequisite for future robustness-oriented extensions. In control-theoretic and model-informed precision dosing frameworks, robust or adaptive optimization fundamentally relies on the existence of an underlying nominal model and corresponding parameter sensitivities.

Importantly, the analytical sensitivity equations derived in this work provide explicit quantitative information regarding how perturbations in PK–PD parameters influence the therapeutic trajectory. This creates a natural foundation for future incorporation of uncertainty-aware methodologies

In particular, repeated therapeutic measurements could be used to periodically update patient-specific PK–PD parameters and re-optimize the remaining dosing strategy online, effectively transforming the present open-loop formulation into an adaptive feedback optimization framework.

Concern:

Practical integration: although formulation and manufacturing constraints are discussed qualitatively, they are not incorporated into the optimization (e.g., discrete dose sets, limited formulations). (Editor: Clarify how practical constraints such as discrete dose levels, limited formulation choices, or manufacturability constraints could be incorporated into future extensions of the optimization framework.)

Response:

We fully agree that the current formulation does not explicitly incorporate practical pharmaceutical constraints such as discrete dose strengths, finite formulation sets, manufacturability limits, or technological formulation restrictions.

The primary objective of the present work was to establish a mathematically structured continuous optimization framework enabling analytical sensitivity derivation and efficient optimization of repeated oral dosing profiles. Consequently, dose magnitude and liberation-rate parameters were intentionally modeled as continuous optimization variables in order to obtain a tractable nonlinear least-squares formulation and derive structured Jacobian equations.

We agree that practical pharmaceutical implementation would require incorporation of additional constraints.

To address this concern, the revised manuscript now explicitly clarifies that the present optimization problem can be interpreted as a continuous relaxation of a more general constrained pharmaceutical co-design problem.

Importantly, the proposed framework naturally supports future incorporation of such practical constraints. In particular:

- discrete dose levels may be introduced through mixed-integer optimization formulations,
- finite formulation choices may be represented using discrete candidate sets,
- manufacturability limitations may be incorporated as inequality constraints on liberation dynamics,
- and clinically acceptable dosing schedules may be enforced through constrained optimization or regularization terms.

Because the present framework already provides analytical state sensitivities and structured optimization equations, these extensions can be incorporated systematically without fundamentally altering the underlying PK–PD modeling methodology.

The revised manuscript now includes an expanded discussion of these translational and manufacturability aspects and clarifies the relationship between the present continuous formulation and future clinically implementable constrained optimization strategies.

Practical implementation and constrained pharmaceutical optimization

In practical pharmaceutical applications, several additional constraints must typically be considered. These include:

- discrete tablet or capsule strengths,
- limited sets of available formulations,
- manufacturable release kinetics,
- excipient and technological limitations,
- and clinically acceptable administration schedules.

Consequently, the present methodology should be interpreted as a continuous relaxation of a more general constrained pharmaceutical co-design problem. Importantly, the proposed framework naturally enables systematic incorporation of such practical constraints.

Discrete dose strengths may be introduced through mixed-integer optimization formulations in which admissible dose values are selected from predefined therapeutic sets. Similarly, finite formulation choices may be represented through discrete candidate release profiles corresponding to available pharmaceutical technologies.

Manufacturability limitations may additionally be incorporated as inequality constraints imposed on liberation dynamics, release-rate bounds, smoothness of dissolution profiles, or achievable kinetic parameters associated with specific drug delivery technologies.

Under such conditions, the resulting optimization problem becomes a hybrid continuous-discrete optimization problem, potentially requiring mixed-integer nonlinear programming (MINLP), branch-and-bound techniques, evolutionary optimization, or hierarchical optimization methodologies.

Nevertheless, the analytical sensitivity framework derived in the present work remains highly valuable even in such constrained settings because it provides local gradient information for the continuous components of the optimization problem and substantially reduces the dimensionality of the clinically relevant decision space.
