# Supplementary material for: Optimal design of dose and drug pharmacokinetic characteristics to achieve the desired pharmacodynamic profile in repeated drug dosing
Source: PLoS One. 2026 Jul 22;21(7):e0354029. doi: 10.1371/journal.pone.0354029 (PMC13390843; doi:10.1371/journal.pone.0354029)
Supplement: Attachment — Submitted filename: Review response 3.pdf [file pone.0354029.s004.pdf]

# Revision notes on the manuscript PONE-D-26-06346R2 entitled Optimal design of dose and drug pharmacokinetic characteristics to achieve the desired pharmacodynamic profile in repeated drug dosing, submitted to PLOS One

Reviewer 3:

**Concern 1: Limited conceptual novelty:**
While now better positioned, the work remains largely an integration of known elements (PK-PD modeling, impulsive control, least-squares optimization). The main novelty (joint optimization of dose and liberation rate within a sensitivity-based framework) is valid but incremental. Claims of generality and "unified framework" are still somewhat overstated.

**Response:**

We thank the reviewer for this valuable observation. In response, we have revised the manuscript to moderate claims regarding novelty, generality, and the scope of applicability of the proposed methodology.

Specifically, references to a "unified framework", "general PK–PD framework", and similar broad formulations have been replaced by more precise descriptions emphasizing that the proposed approach is a structured optimization methodology for repeated oral dosing based on linear compartmental pharmacokinetic models coupled with static concentration–effect relationships. We now explicitly acknowledge that the work does not introduce a new pharmacokinetic model, pharmacodynamic model, or optimization algorithm, but rather integrates these established components into a sensitivity-based optimization framework.

To better clarify the methodological contribution, we have strengthened the discussion of the primary novelty of the work, namely the simultaneous optimization of dose magnitude and formulation-dependent liberation kinetics. In particular, we emphasize that, unlike conventional dosing optimization approaches that typically assume fixed formulation characteristics, the proposed methodology treats liberation rate as an explicit decision variable and therefore enables joint optimization of both therapy design and formulation-dependent release characteristics.

See the revised manuscript with tracked changes.

**Concern 2: Lack of quantitative robustness analysis:**
Although limitations are discussed, no numerical or theoretical analysis is provided to quantify sensitivity of the optimal solution to parameter perturbations. This remains a notable gap for a method intended for decision support.

**Response:**

We thank the reviewer for this important suggestion. In the revised manuscript, we have added a dedicated quantitative robustness analysis to assess the sensitivity of the proposed optimal dosing strategy with respect to pharmacokinetic parameter perturbations.

Specifically, we performed a Monte Carlo simulation study in which key PK parameters (absorption and elimination rates $k_a$, $k_{e1}$, $k_{23}$, and $k_{e3}$) were perturbed within ±30% uniform random variation. For each realization (patient), the full nonlinear least squares problem was re-solved using the Gauss–Newton optimization framework, and the resulting optimal dosing parameters and pharmacodynamic trajectories were evaluated.

The results demonstrate that the optimization algorithm is robust under parameter uncertainty. In particular, the variability in optimized dosing parameters remains bounded, and the induced pharmacodynamic response $E(t)$ preserves its agreement with the reference trajectory $E_r(t)$, with some dispersion around the mean response.

The new results and corresponding discussion have been added to the revised manuscript.

*To assess the robustness of the proposed optimal dosing framework beyond a single nominal parameter configuration, additional simulations were conducted under a set of different pharmacokinetic settings in terms of Monte Carlo analysis. Specifically, the absorption and elimination rate constants, as well as inter-compartmental transfer rates, were systematically varied to simulate a patient population with varying PK characteristics. The key non-optimizable patient-specific PK parameters, the rate constants $k_a$, $k_{e1}$, $k_{23}$, and $k_{e3}$, were independently perturbed using a uniform random distribution within ±30 of their nominal values.*

*In the first scenario, for each of $n_{MC} = 50$ realizations, the nonlinear least squares problem of the therapy design was re-solved using the Gauss–Newton optimization framework, resulting in a corresponding personalized optimal dosing policy $\boldsymbol{\theta}^*$.*

*The resulting distribution of optimized treatment parameters $\boldsymbol{\theta}^*$ depicted in Fig 10. indicates that the proposed optimization algorithm remains stable under parameter perturbations, with bounded variability of parameters across all dosing intervals. The most significant variability was observed in the magnitude of the leading dose. As demonstrated in Fig 11., despite variability in the PK dynamics, the resulting pharmacodynamic response $E(t)$ remains closely aligned with the desired reference trajectory $E_r(t)$. In particular, the ensemble mean response $E(t)$ (Mean) closely follows the nominal solution from Fig 7., while*

*the standard deviation $E(t) \pm STD$ remains relatively small throughout the dosing horizon, indicating moderate sensitivity to parameter variations. These results demonstrate that the proposed impulsive PK–PD optimization framework provides consistent therapeutic performance despite variations in pharmacokinetic parameters.*

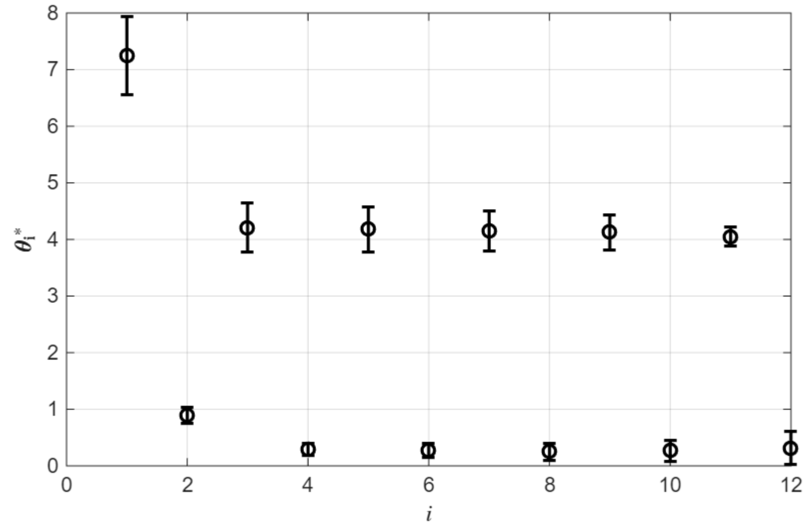


Fig 10. Distribution of optimized treatment parameters $\boldsymbol{\theta}^*$ obtained under Monte Carlo analysis of patient population with varying PK characteristics

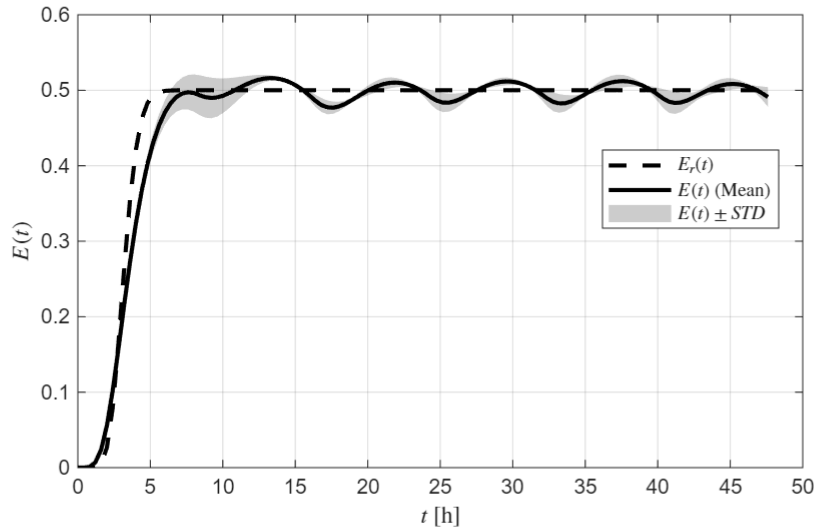


Fig 11. Ensemble mean pharmacodynamic response and its standard deviation obtained under Monte Carlo analysis of patient population with varying PK characteristics

*In the second scenario, the nominal-patient optimal therapy defined by Eq. (39) was applied to each of the $n_{MC} = 50$ Monte Carlo realizations in order to evaluate the robustness of the dosing recommendation under pharmacokinetic parameter uncertainty.*

*As shown in Fig. 12, the resulting pharmacodynamic response $E(t)$ remains satisfactory despite the uncertainty in the PK dynamics. However, compared with the personalized optimization results presented in Fig. 11, the variability of the response, quantified by the standard deviation $E(t) \pm$ STD, is increased. The standard deviation reaches approximately $\pm 0.05$ in terms of the relative therapeutic effect $E(t)$, corresponding to approximately $\pm 10\%$ variability with respect to the target therapeutic effect $0.5$. Despite this increased variability, the pharmacodynamic response remains within an acceptable range, indicating a reasonable degree of robustness of the optimal dosing regimen with respect to pharmacokinetic parameter uncertainty. In particular, the observed deviations do not appear to induce clinically relevant underdosing or toxicity within the considered simulation setting.*

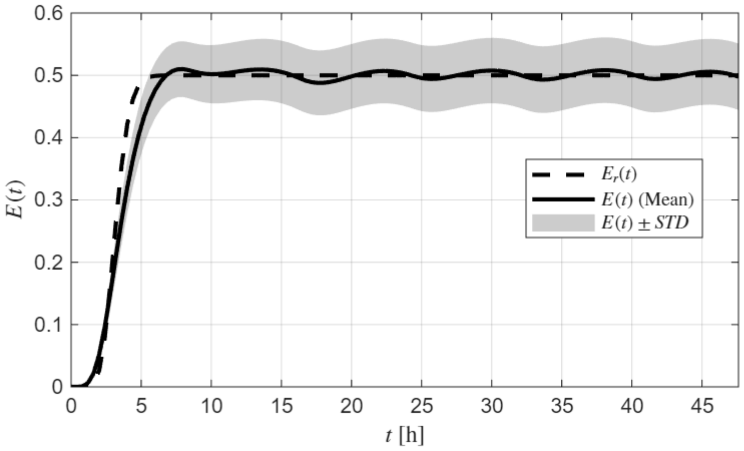


Fig 12. Ensemble mean pharmacodynamic response and its standard deviation obtained under Monte Carlo analysis of patient population with varying PK characteristics and nominal-patient optimal therapy

**Concern 3: Local nature of the optimization:**

The reliance on local sensitivities and Gauss-Newton is acknowledged, but there is no discussion of convergence, possible nonconvexity, or dependence on initialization. This is particularly relevant given the nonlinear PD mapping.

**Response:**

We thank the reviewer for this important observation. We agree that the original manuscript did not sufficiently discuss the implications of using a Gauss-Newton optimization approach. The optimization problem is generally nonconvex due to the nonlinear Hill-type pharmacodynamic mapping (nonlinear least squares), and therefore the Gauss-Newton algorithm provides only local convergence guarantees. Consequently, the obtained solution should be interpreted as a locally optimal solution whose final outcome may depend on the initialization.

To address this limitation, we have expanded the discussion in the results section and analyzed the effect of initialization on the convergence.

*Because the resulting optimization problem is generally nonconvex, the Gauss–Newton algorithm provides only local convergence guarantees. To investigate the influence of initialization, the optimization was repeated from 100 randomly perturbed initial parameter vectors. Each initialization was generated by independently perturbing the nominal initial guess for every optimized dose magnitude and liberation rate by a uniformly distributed random factor within ±50% of its nominal value, while keeping the fixed pharmacokinetic parameters unchanged.*

Fig. 9 presents a histogram of the final objective function values obtained from 100 optimization runs initialized from randomly generated parameter vectors. The distribution indicates that despite the substantial variability of the starting points, all optimization runs converged to very similar objective function values and yielded highly similar optimal dosing regimens. This behavior indicates that, within the tested initialization region, the optimization landscape does not exhibit practically significant competing local minima and that the obtained solution is only weakly dependent on initialization. The small differences observed among the final objective function values are likely attributable to the stopping criterion of the Gauss–Newton algorithm, which is based on the norm of the gradient rather than on a prescribed residual tolerance. Consequently, individual runs may terminate at slightly different points within the same local neighborhood of the optimum, leading to minor variations in the final objective values. Although a formal guarantee of global optimality cannot be provided, these results suggest that the proposed sensitivity-based Gauss–Newton procedure exhibits robust convergence toward the same locally optimal solution for clinically relevant initial parameter guess.

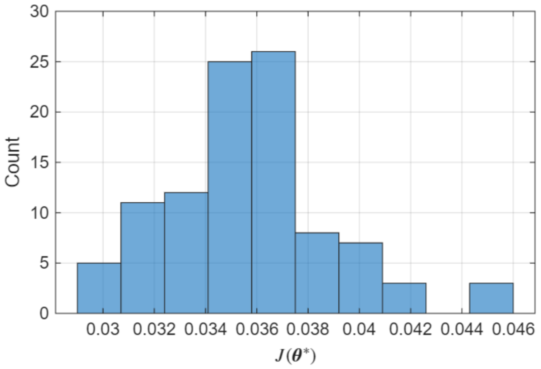


Fig 9. Histogram of the final objective function values obtained from multiple optimization runs initialized from randomly generated parameter vectors

**Concern 4: Validation limited to a single in-silico example:**
The illustrative example is clear but does not sufficiently demonstrate generality or robustness across scenarios (e.g., different PK structures or parameter regimes).

**Response:**

We agree that validation on a single nominal parameter setting may not fully demonstrate the generality of the proposed framework. To address this concern, we have extended the manuscript discussion and results to better clarify the applicability of the method across different pharmacokinetic characteristics.

In particular, we emphasize that the proposed impulsive optimal control formulation is not tied to a specific parameter set, but is instead defined on a general linear compartmental pharmacokinetic structure. To support this, we have performed additional simulations under mutiple substantially different pharmacokinetic parameter regimes, including variations in absorption and elimination rates as well as altered inter-compartmental transfer dynamics. Across all tested regimes (generated virtual patient population), the proposed Gauss–Newton-based optimization framework consistently converged and produced dosing strategies exhibiting the comparable qualitative structure (initial loading dose followed by smaller maintenance doses) and comparable tracking performance.

The corresponding Monte Carlo uncertainty analysis demonstrated that the proposed method is not restricted to a single numerical configuration, but rather exhibits consistent behavior across different pharmacokinetic parameter regimes.

See the response to Concern 2, for new added results.

Besides the quantitative Monte Carlo (statistical) analysis related to Concern 2, we added another interesting PK scenario to demonstrate how the optimal treatment would change with respect to the patient characteristics:

*As an additional illustrative example, a scenario with impaired drug absorption was considered. This condition was modeled by reducing the absorption rate constant to ($k_a = 0.01$ 1/h), representing substantially slower transfer of the drug into the systemic circulation. From a pharmacological perspective, impaired absorption is expected to require larger administered doses to achieve the same therapeutic effect. Furthermore, faster drug liberation may be advantageous to compensate, at least partially, for the delayed absorption and to maintain the onset of action prescribed by the reference pharmacodynamic trajectory ($E_r(t)$).*

*By optimizing the full dosing regime, which includes the dose size $D$ and the liberation rate $k_l$ for each dosing event in terms of Eq (33), the following parameters were determined*

$$\theta^1 = \begin{pmatrix} 24.65 \\ 0.94 \end{pmatrix}, \theta^2 = \begin{pmatrix} 12.3892 \\ 0.21 \end{pmatrix}, \theta^3 = \begin{pmatrix} 11.57 \\ 0.25 \end{pmatrix}, \theta^4 = \begin{pmatrix} 11.00 \\ 0.24 \end{pmatrix}, \theta^5 = \begin{pmatrix} 10.26 \\ 0.22 \end{pmatrix}, \theta^6 = \begin{pmatrix} 8.99 \\ 0.20 \end{pmatrix} \quad (40).$$

*Comparison with the optimal solution presented in Eq (39) shows that the optimized doses are approximately three times larger, while the corresponding liberation rates are approximately 20% higher. These changes are consistent with the reduced absorption capacity of the patient and illustrate how the proposed optimization framework automatically adapts both the administered dose and the formulation-dependent liberation kinetics to maintain the desired therapeutic response.*

*The resulting optimal pharmacodynamic profile is shown in Fig. 13. Despite the substantially impaired absorption, the optimized treatment strategy achieves a therapeutic response that remains closely aligned with the reference trajectory ($E_r(t)$), exhibiting behavior qualitatively similar to that observed in Fig. 7.*

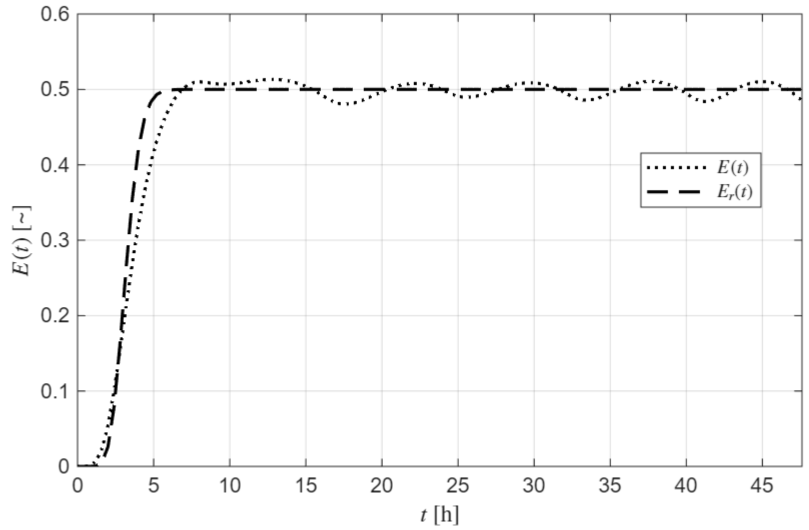


*Fig 13. Optimal therapeutic profile with variable dose size $D$ mg and dose-dependent liberation rate $k_l$ under impaired absorption ($k_a = 0.01$ 1/h)*
