# Supplementary material for: Optimal design of dose and drug pharmacokinetic characteristics to achieve the desired pharmacodynamic profile in repeated drug dosing
Source: PLoS One. 2026 Jul 22;21(7):e0354029. doi: 10.1371/journal.pone.0354029 (PMC13390843; doi:10.1371/journal.pone.0354029)
Supplement: Peer review history

# Peer review: Decision Letter 0

**DOI:** 10.1371/journal.pone.0354029.r001

**Authors:** Pasquale Palumbo

8 Mar 2026

PONE-D-26-06346

Optimal design of dose and drug pharmacokinetic characteristics to achieve the desired pharmacodynamic profile in repeated drug dosing

PLOS One

Dear Dr. Dodek,

Thank you for submitting your manuscript to PLOS ONE. After careful consideration, we feel that it has merit but does not fully meet PLOS ONE’s publication criteria as it currently stands. Therefore, we invite you to submit a revised version of the manuscript that addresses the points raised during the review process.

Please submit your revised manuscript by Apr 22 2026 11:59PM. If you will need more time than this to complete your revisions, please reply to this message or contact the journal office at plosone@plos.org. When you're ready to submit your revision, log on to https://www.editorialmanager.com/pone/ and select the 'Submissions Needing Revision' folder to locate your manuscript file.

Please include the following items when submitting your revised manuscript:

- A letter that responds to each point raised by the academic editor and reviewer(s). You should upload this letter as a separate file labeled 'Response to Reviewers'.
- A marked-up copy of your manuscript that highlights changes made to the original version. You should upload this as a separate file labeled 'Revised Manuscript with Track Changes'.
- An unmarked version of your revised paper without tracked changes. You should upload this as a separate file labeled 'Manuscript'.

If you would like to make changes to your financial disclosure, please include your updated statement in your cover letter. Guidelines for resubmitting your figure files are available below the reviewer comments at the end of this letter.

If applicable, we recommend that you deposit your laboratory protocols in protocols.io to enhance the reproducibility of your results. Protocols.io assigns your protocol its own identifier (DOI) so that it can be cited independently in the future. For instructions see: https://journals.plos.org/plosone/s/submission-guidelines#loc-laboratory-protocols. Additionally, PLOS ONE offers an option for publishing peer-reviewed Lab Protocol articles, which describe protocols hosted on protocols.io. Read more information on sharing protocols at https://plos.org/protocols?utm_medium=editorial-email&utm_source=authorletters&utm_campaign=protocols.

We look forward to receiving your revised manuscript.

Kind regards,

Pasquale Palumbo

Academic Editor

PLOS One

Journal Requirements:

When submitting your revision, we need you to address these additional requirements.

1. Please ensure that your manuscript meets PLOS ONE's style requirements, including those for file naming. The PLOS ONE style templates can be found at

https://journals.plos.org/plosone/s/file?id=wjVg/PLOSOne_formatting_sample_main_body.pdf and

https://journals.plos.org/plosone/s/file?id=ba62/PLOSOne_formatting_sample_title_authors_affiliations.pdf

2. Please note that PLOS One has specific guidelines on code sharing for submissions in which author-generated code underpins the findings in the manuscript. In these cases, we expect all author-generated code to be made available without restrictions upon publication of the work. Please review our guidelines at https://journals.plos.org/plosone/s/materials-and-software-sharing#loc-sharing-code and ensure that your code is shared in a way that follows best practice and facilitates reproducibility and reuse.

3. Please provide a complete Data Availability Statement in the submission form, ensuring you include all necessary access information or a reason for why you are unable to make your data freely accessible. If your research concerns only data provided within your submission, please write "All data are in the manuscript and/or supporting information files" as your Data Availability Statement.

4. If the reviewer comments include a recommendation to cite specific previously published works, please review and evaluate these publications to determine whether they are relevant and should be cited. There is no requirement to cite these works unless the editor has indicated otherwise.

Additional Editor Comments:

After careful evaluation, the manuscript has been reviewed by two experts in the field. While both reviewers acknowledge the clarity of mathematical derivations and the potential interest of applying control-theoretic approaches to pharmacokinetic/pharmacodynamic optimization, they have raised several important concerns that need to be addressed before the manuscript can be considered further.

Major Points to Address:

Positioning within the literature:

Both reviewers suggest that the manuscript would benefit from a clearer discussion of how your framework compares with existing PK/PD dose optimization methods. In particular, please consider including recent references suggested by Reviewer 1 (e.g., Chua & Tam, 2022; Chotsiri et al., 2025) and any other relevant studies that situate your approach within the broader PK/PD and optimal control literature.

Practical applicability

Reviewer 1 notes that your proposed optimized dosing strategy involves variable doses at each administration, which may be difficult to implement in real-world clinical practice. Both reviewers highlight that inter-patient variability, patient compliance, and manufacturing constraints (fixed dose strengths and release profiles) are important factors. Please clarify the scope of your approach and discuss how these practical constraints may influence the application or interpretation of your results.

Demonstration of advantage

Reviewer 1 requests a comparison between the proposed optimized dosing regimen and simpler, more standard strategies (e.g., constant-dose regimens) to illustrate the potential practical advantage of your approach.

Mathematical and technical clarifications

Reviewer 2 raised a number of technical points regarding notation, definitions, and the presentation of certain assumptions (e.g., the definition of variables, the constancy of parameters between doses, and characteristic time scales). Please carefully review the manuscript and clarify these aspects. Address the specific points noted in Reviewer 2’s comments to ensure mathematical rigor and clarity.

Editorial improvements

A thorough proofreading of the manuscript is recommended. Additionally, Reviewer 2 suggested providing references to support clinical claims, such as statements about the logistic form reflecting clinicians’ conceptualization of therapeutic courses.

In summary, the primary revisions required concern positioning, discussion, practical interpretation, and clarity, rather than fundamental methodological errors.

[Note: HTML markup is below. Please do not edit.]

Reviewers' comments:

Reviewer's Responses to Questions

**Comments to the Author**

1. Is the manuscript technically sound, and do the data support the conclusions?

The manuscript must describe a technically sound piece of scientific research with data that supports the conclusions. Experiments must have been conducted rigorously, with appropriate controls, replication, and sample sizes. The conclusions must be drawn appropriately based on the data presented.

Reviewer #1: Partly

Reviewer #2: Partly

**********

2. Has the statistical analysis been performed appropriately and rigorously?

Reviewer #1: N/A

Reviewer #2: N/A

**********

3. Have the authors made all data underlying the findings in their manuscript fully available?

The PLOS Data policy requires authors to make all data underlying the findings described in their manuscript fully available without restriction, with rare exception (please refer to the Data Availability Statement in the manuscript PDF file). The data should be provided as part of the manuscript or its supporting information, or deposited to a public repository. For example, in addition to summary statistics, the data points behind means, medians and variance measures should be available. If there are restrictions on publicly sharing data—e.g. participant privacy or use of data from a third party—those must be specified.

Reviewer #1: Yes

Reviewer #2: Yes

**********

4. Is the manuscript presented in an intelligible fashion and written in standard English?

PLOS ONE does not copyedit accepted manuscripts, so the language in submitted articles must be clear, correct, and unambiguous. Any typographical or grammatical errors should be corrected at revision, so please note any specific errors here.

Reviewer #1: Yes

Reviewer #2: No

**********

5. Review Comments to the Author

Please use the space provided to explain your answers to the questions above. You may also include additional comments for the author, including concerns about dual publication, research ethics, or publication ethics. (Please upload your review as an attachment if it exceeds 20,000 characters)

Reviewer #1: The manuscript illustrates the application of control theory to identifying optimal doses, based on pharmacodynamic criteria. However, major challenges for optimising dosing in patients are a) Inter-patient variability in pharmacokinetics and pharmacodynamics, b) Patient compliance (i.e. make the regimen as simple as possible) and c) commercial and logistic feasibility (minimise the number of dose strengths that have to manufactured, shipped and kept in stock by pharmacists). None of these aspects have been considered by this publication.

There are also some useful recent publications that are omitted from the references that would provide a broader view of the research landscape:

1. Chua HC, Tam VH. Optimizing Clinical Outcomes Through Rational Dosing Strategies: Roles of Pharmacokinetic/Pharmacodynamic Modeling Tools. Open Forum Infect Dis. 2022 Dec 16;9(12):ofac626. doi: 10.1093/ofid/ofac626.

2. Chotsiri P, Yodsawat P, Hoglund RM, Simpson JA, Tarning J. Pharmacometric and statistical considerations for dose optimization. CPT Pharmacometrics Syst Pharmacol. 2025; 14: 279-291. doi:10.1002/psp4.13271

The proposed optimal solution for the case study proposes administering a different dose level at each administration, which is not particularly feasible. The authors should demonstrate the increase of optimality of this approach vs the same dose level at each administration.

Reviewer #2: The manuscript presents a framework for optimizing repeated oral drug dosing and formulation-dependent release rates by integrating a linear compartmental pharmacokinetic (PK) model with a nonlinear Hill-type pharmacodynamic (PD) model using impulsive control theory and a Gauss-Newton nonlinear least-squares algorithm. While the mathematical derivation is clear, the core problem of optimizing dosing regimens to achieve a desired therapeutic profile is already well-established and broadly solved in the existing literature. For example, the study by Bachmann et al. (OptiDose: Computing the Individualized Optimal Drug Dosing Regimen Using Optimal Control. J Optim Theory Appl 189, 46–65, 2021; https://doi.org/10.1007/s10957-021-01819-w) already addresses this optimal control challenge in a much more general and comprehensive manner. In contrast, this manuscript focuses on a very specific and simplified scenario restricted to linear pharmacokinetics and static Hill-function pharmacodynamics, completely neglecting the underlying disease dynamics. Furthermore, since the proposed optimization task is performed offline rather than in real-time, the computational burden is minimal, meaning the problem can be easily handled by any standard off-the-shelf numerical optimizer. Consequently, because the problem is not computationally difficult and the approach lacks broader applicability, the explicitly derived sensitivity formulas and Jacobians offer limited new scientific value or methodological advancement to the field.

My other comments:

1. The practical applicability of the proposed method is unclear. While the authors emphasize oral dosing in the introduction, prescribing an exact optimal dose is strictly limited by commercially available pill sizes (i.e., patients can only take predefined doses or perhaps halve the pills, restricting the input to discrete values). Furthermore, adjusting the liberation rate per dosing event is even more unrealistic due to manufacturing constraints, as pills are produced with fixed release profiles. The authors should clarify how this continuous optimization approach can be translated into real-world clinical practice.

2. In lines 104-108, the authors state: "Impulsive control theory has also been applied... These methods, however, are inherently conservative and typically converge to uniform dosing patterns." Interestingly, the results of this manuscript ultimately converge to a very similar uniform pattern. In lines 581-583, the authors conclude: "The most important conclusion is that the optimal therapy constitutes of a larger leading dose... followed by smaller maintenance doses with much slower liberation, which is actually in compliance with the basic intuition." Thus, the final outcome of the proposed method does not significantly differ from the standard approaches the authors criticize.

3. Lines 181-182: "The Dirac impulse defined in Eq (2) provides an appropriate abstraction for oral drug dosing, as such therapies consist of intermittent, event-driven interventions." The use of the term "event-driven" is misleading here. According to Eq (2), drug administration occurs at strictly fixed, equidistant time intervals ($T_D$), making it time-triggered rather than event-driven. Moreover, assuming perfectly equidistant dosing intervals is clinically unrealistic, as patient compliance rarely allows for taking pills at the exact prescribed minute. The authors should justify why fixing the dosing intervals is necessary for their algorithm.

4. Lines 182-185: "Each administration introduces the prescribed dose into the gastrointestinal compartment over a time interval that is negligible compared to the characteristic time scales of pharmacokinetic processes." The authors should be more precise mathematically. Do they mean that the duration of drug intake is negligible compared to the smallest time constant (or half-life) of the PK model? A clearer mathematical or physiological definition of these "characteristic time scales" would be helpful.

5. The authors should explicitly state that the parameters $\theta^i$ remain constant between the consecutive dosing injections (discrete events).

6. The variable $j$ in Eq (4) is undefined. Does it represent the number of administered injections up to time $t$? This needs to be explicitly defined. Furthermore, $j$ is reused as a parameter index in Eq (14), which causes mathematical ambiguity and should be corrected.

7. Hyphens and dashes are used incorrectly or interchangeably in several places, for example in lines 283 and 427. Please review the punctuation for standard academic formatting.

8. Definite articles are missing in several places throughout the manuscript. For instance, in line 139: "One of central" should be "One of the central"; and in line 249: "Consider vector" should be "Consider the vector". A thorough proofreading is recommended.

9. Lines 466-469: "The logistic form was selected because it naturally reflects how clinicians conceptualize an ideal therapeutic course..." This is a strong clinical claim. Please provide appropriate references from the medical or pharmacological literature to support the statement that clinicians conceptualize the ideal therapeutic course in this specific mathematical form.

**********

6. PLOS authors have the option to publish the peer review history of their article (what does this mean?). If published, this will include your full peer review and any attached files.

If you choose “no”, your identity will remain anonymous but your review may still be made public.

**Do you want your identity to be public for this peer review?**  For information about this choice, including consent withdrawal, please see our Privacy Policy.

Reviewer #1: No

Reviewer #2: No

**********

[NOTE: If reviewer comments were submitted as an attachment file, they will be attached to this email and accessible via the submission site. Please log into your account, locate the manuscript record, and check for the action link "View Attachments". If this link does not appear, there are no attachment files.]

To ensure your figures meet our technical requirements, please review our figure guidelines: https://journals.plos.org/plosone/s/figures

You may also use PLOS’s free figure tool, NAAS, to help you prepare publication quality figures: https://journals.plos.org/plosone/s/figures#loc-tools-for-figure-preparation.

NAAS will assess whether your figures meet our technical requirements by comparing each figure against our figure specifications.

# Author comment: Author response to Decision Letter 1

**DOI:** 10.1371/journal.pone.0354029.r002

8 Apr 2026

See file Review response.pdf for complete response to the reviewers

Attachment Submitted filename: Review response.pdf

# Peer review: Decision Letter 1

**DOI:** 10.1371/journal.pone.0354029.r003

**Authors:** Pasquale Palumbo

15 May 2026

PONE-D-26-06346R1

Optimal design of dose and drug pharmacokinetic characteristics to achieve the desired pharmacodynamic profile in repeated drug dosing

PLOS One

Dear Dr. Dodek,

Thank you for submitting your manuscript to PLOS ONE. After careful consideration, we feel that it has merit but does not fully meet PLOS ONE’s publication criteria as it currently stands. Therefore, we invite you to submit a revised version of the manuscript that addresses the points raised during the review process.

Please submit your revised manuscript by Jun 29 2026 11:59PM. If you will need more time than this to complete your revisions, please reply to this message or contact the journal office at plosone@plos.org. When you're ready to submit your revision, log on to https://www.editorialmanager.com/pone/ and select the 'Submissions Needing Revision' folder to locate your manuscript file.

Please include the following items when submitting your revised manuscript:

- A letter that responds to each point raised by the academic editor and reviewer(s). You should upload this letter as a separate file labeled 'Response to Reviewers'.
- A marked-up copy of your manuscript that highlights changes made to the original version. You should upload this as a separate file labeled 'Revised Manuscript with Track Changes'.
- An unmarked version of your revised paper without tracked changes. You should upload this as a separate file labeled 'Manuscript'.

If you would like to make changes to your financial disclosure, please include your updated statement in your cover letter. Guidelines for resubmitting your figure files are available below the reviewer comments at the end of this letter.

If applicable, we recommend that you deposit your laboratory protocols in protocols.io to enhance the reproducibility of your results. Protocols.io assigns your protocol its own identifier (DOI) so that it can be cited independently in the future. For instructions see: https://journals.plos.org/plosone/s/submission-guidelines#loc-laboratory-protocols. Additionally, PLOS ONE offers an option for publishing peer-reviewed Lab Protocol articles, which describe protocols hosted on protocols.io. Read more information on sharing protocols at https://plos.org/protocols?utm_medium=editorial-email&utm_source=authorletters&utm_campaign=protocols.

As the corresponding author, your ORCID iD is verified in the submission system and will appear in the published article. PLOS supports the use of ORCID, and we encourage all coauthors to register for an ORCID iD and use it as well. Please encourage your coauthors to verify their ORCID iD within the submission system before final acceptance, as unverified ORCID iDs will not appear in the published article. *Only*  the individual author can complete the verification step; PLOS staff *cannot*  verify ORCID iDs on behalf of authors.

We look forward to receiving your revised manuscript.

Kind regards,

Pasquale Palumbo

Academic Editor

PLOS One

Journal Requirements:

1. If the reviewer comments include a recommendation to cite specific previously published works, please review and evaluate these publications to determine whether they are relevant and should be cited. There is no requirement to cite these works unless the editor has indicated otherwise.

2. Please review your reference list to ensure that it is complete and correct. If you have cited papers that have been retracted, please include the rationale for doing so in the manuscript text, or remove these references and replace them with relevant current references. Any changes to the reference list should be mentioned in the rebuttal letter that accompanies your revised manuscript. If you need to cite a retracted article, indicate the article’s retracted status in the References list and also include a citation and full reference for the retraction notice.

Additional Editor Comments:

The manuscript has undergone an additional round of evaluation. One of the original reviewers, who had raised concerns during the first review round, is now satisfied with the revisions and supports publication. Another original reviewer, who had provided a strongly critical assessment in the first round, did not provide a second-round report. Because of the divergence in the initial reviews, an additional independent evaluation was requested.

The additional review considers the manuscript technically sound, mathematically consistent, and clearly written, with a solid systems-and-control perspective. In particular, the following aspects were identified as meaningful contributions:

the joint optimization of dose magnitude and formulation/liberation rate,

the analytical derivation of sensitivities and Jacobian structure,

the direct optimization of the pharmacodynamic trajectory.

At the same time, the contribution is viewed primarily as an incremental methodological advance rather than a fundamentally new paradigm. Several important limitations and assumptions should therefore be discussed more explicitly in the manuscript.

The revised version should address the following points:

Model dependence and identifiability:

Expand the discussion regarding the dependence of the proposed optimization framework on the accuracy of the underlying PK/PD model. In particular, discuss issues related to parameter identifiability, conditioning of the Jacobian, and sensitivity to model mismatch.

Structural assumptions and limitations:

The framework relies on several simplifying assumptions, including: linear pharmacokinetics, static Hill-type pharmacodynamics, fixed dosing intervals, impulsive dosing approximation.

These assumptions should be more clearly presented as methodological restrictions defining the scope of applicability of the proposed approach.

Robustness and uncertainty:

Include a discussion on robustness with respect to uncertainty in PK/PD parameters and modeling errors. Even if a complete robustness analysis is beyond the scope of the present work, the limitations of the current open-loop formulation should be acknowledged explicitly.

Practical constraints and implementation:

Clarify how practical constraints such as discrete dose levels, limited formulation choices, or manufacturability constraints could be incorporated into future extensions of the optimization framework.

[Note: HTML markup is below. Please do not edit.]

Reviewers' comments:

Reviewer's Responses to Questions

**Comments to the Author**

1. If the authors have adequately addressed your comments raised in a previous round of review and you feel that this manuscript is now acceptable for publication, you may indicate that here to bypass the “Comments to the Author” section, enter your conflict of interest statement in the “Confidential to Editor” section, and submit your "Accept" recommendation.

Reviewer #1: All comments have been addressed

Reviewer #3: (No Response)

**********

2. Is the manuscript technically sound, and do the data support the conclusions?

The manuscript must describe a technically sound piece of scientific research with data that supports the conclusions. Experiments must have been conducted rigorously, with appropriate controls, replication, and sample sizes. The conclusions must be drawn appropriately based on the data presented.

Reviewer #1: Yes

Reviewer #3: Yes

**********

3. Has the statistical analysis been performed appropriately and rigorously?

Reviewer #1: N/A

Reviewer #3: Yes

**********

4. Have the authors made all data underlying the findings in their manuscript fully available?

The PLOS Data policy requires authors to make all data underlying the findings described in their manuscript fully available without restriction, with rare exception (please refer to the Data Availability Statement in the manuscript PDF file). The data should be provided as part of the manuscript or its supporting information, or deposited to a public repository. For example, in addition to summary statistics, the data points behind means, medians and variance measures should be available. If there are restrictions on publicly sharing data—e.g. participant privacy or use of data from a third party—those must be specified.

Reviewer #1: Yes

Reviewer #3: Yes

**********

5. Is the manuscript presented in an intelligible fashion and written in standard English?

PLOS ONE does not copyedit accepted manuscripts, so the language in submitted articles must be clear, correct, and unambiguous. Any typographical or grammatical errors should be corrected at revision, so please note any specific errors here.

Reviewer #1: Yes

Reviewer #3: Yes

**********

6. Review Comments to the Author

Please use the space provided to explain your answers to the questions above. You may also include additional comments for the author, including concerns about dual publication, research ethics, or publication ethics. (Please upload your review as an attachment if it exceeds 20,000 characters)

Reviewer #1: All of my comments have noew been addressed. MThe authors' efforts are appreciated. The manuscript is much clearer now.

Reviewer #3: This manuscript presents a mathematically consistent framework for optimizing repeated oral dosing by combining linear compartmental PK models, nonlinear Hill-type PD, impulsive control, and sensitivity-based Gauss–Newton optimization. The work is clearly written and technically competent, with a strong systems-and-control perspective.

The core elements—compartmental PK, Hill PD models, impulsive dosing, and nonlinear least-squares optimization—are individually well established. Optimal dosing via control-theoretic or optimal-control frameworks (including adjoint-based approaches such as OptiDose) and exposure-based PK/PD metrics (AUC/MIC, Cmax/MIC) form a mature literature.

Accordingly, the manuscript does not introduce a fundamentally new modeling paradigm. Its novelty is better characterized as a structured integration and reformulation of existing ideas.

The main genuine and relevant contributions are:

1. Joint optimization of dose magnitude and formulation parameter (liberation rate).

Unlike most PK/PD optimization approaches that treat PK parameters as fixed, the inclusion of a per-dose liberation rate as an optimization variable is a meaningful extension, effectively enlarging the control/design space. This coupling between dosing and formulation is relatively uncommon and constitutes the strongest conceptual contribution.

2. Reformulation as a structured nonlinear least-squares problem with analytical sensitivities.

By exploiting linear PK and impulsive superposition, the authors derive explicit state–parameter sensitivities and construct an exact Jacobian for the pharmacodynamic output. This enables an efficient Gauss–Newton scheme and provides interpretability that is typically absent in adjoint-based or black-box methods.

While not conceptually new, this is a **methodologically solid and useful refinement**.

3. Direct optimization of the time-domain PD trajectory.

The focus on shaping the full therapeutic effect profile (rather than scalar exposure metrics) aligns the work with control-theoretic approaches and represents a valuable perspective within pharmacometrics, though similar ideas exist in optimal control and MPC formulations.

Overall, the paper should be regarded as an incremental contribution with a notable design-variable extension, rather than a breakthrough.

Technical strengths are a clear formulation of the impulsive PK system and consistent derivation of sensitivity equations and Jacobian; the coherent use of Gauss–Newton tailored to the least-squares structure; the emergence of loading–maintenance strategies from the optimization. The approach is modular and potentially extendable to higher-dimensional compartment models.

Main limitations and concerns:

From a systems/inference perspective, several important aspects are underdeveloped:

1) Model dependence and identifiability: the method assumes an accurately identified PK–PD model, but the optimization relies on local sensitivities. There is no discussion of parameter identifiability, conditioning of the Jacobian, or robustness to model mismatch—critical issues in such inverse problems.

2) Structural assumptions: linearity of PK, static PD mapping, fixed dosing intervals, and impulsive approximation constrain the admissible dynamics; these assumptions limit generality and should be more explicitly framed as restrictions rather than general features.

3) Robustness and uncertainty: the framework is fully open-loop; no analysis is provided on how errors in PK/PD parameters affect the optimal solution.

4) Practical integration: although formulation and manufacturing constraints are discussed qualitatively, they are not incorporated into the optimization (e.g., discrete dose sets, limited formulations).

Conclusion:

The manuscript is technically sound and presents a well-executed integration of control-theoretic and PK–PD modeling tools. Its main novelty lies in the joint optimization of dosing and formulation parameters within a sensitivity-based least-squares framework, supported by an analytically tractable structure. However, the contribution remains incremental rather than conceptual, and its practical impact depends on addressing issues of model uncertainty, identifiability, and constrained optimization. These issues should be addressed!

**********

7. PLOS authors have the option to publish the peer review history of their article (what does this mean?). If published, this will include your full peer review and any attached files.

If you choose “no”, your identity will remain anonymous but your review may still be made public.

**Do you want your identity to be public for this peer review?**  For information about this choice, including consent withdrawal, please see our Privacy Policy.

Reviewer #1: No

Reviewer #3: No

**********

[NOTE: If reviewer comments were submitted as an attachment file, they will be attached to this email and accessible via the submission site. Please log into your account, locate the manuscript record, and check for the action link "View Attachments". If this link does not appear, there are no attachment files.]

To ensure your figures meet our technical requirements, please review our figure guidelines: https://journals.plos.org/plosone/s/figures

You may also use PLOS’s free figure tool, NAAS, to help you prepare publication quality figures: https://journals.plos.org/plosone/s/figures#loc-tools-for-figure-preparation.

NAAS will assess whether your figures meet our technical requirements by comparing each figure against our figure specifications.

# Author comment: Author response to Decision Letter 2

**DOI:** 10.1371/journal.pone.0354029.r004

20 May 2026

See the attached file Review response 2.pdf

Attachment Submitted filename: Review response 2.pdf

# Peer review: Decision Letter 2

**DOI:** 10.1371/journal.pone.0354029.r005

**Authors:** Pasquale Palumbo

26 May 2026

PONE-D-26-06346R2

Optimal design of dose and drug pharmacokinetic characteristics to achieve the desired pharmacodynamic profile in repeated drug dosing

PLOS One

Dear Dr. Dodek,

Thank you for submitting your manuscript to PLOS ONE. After careful consideration, we feel that it has merit but does not fully meet PLOS ONE’s publication criteria as it currently stands. Therefore, we invite you to submit a revised version of the manuscript that addresses the points raised during the review process.

Please submit your revised manuscript by Jul 10 2026 11:59PM. If you will need more time than this to complete your revisions, please reply to this message or contact the journal office at plosone@plos.org. When you're ready to submit your revision, log on to https://www.editorialmanager.com/pone/ and select the 'Submissions Needing Revision' folder to locate your manuscript file.

Please include the following items when submitting your revised manuscript:

- A letter that responds to each point raised by the academic editor and reviewer(s). You should upload this letter as a separate file labeled 'Response to Reviewers'.
- A marked-up copy of your manuscript that highlights changes made to the original version. You should upload this as a separate file labeled 'Revised Manuscript with Track Changes'.
- An unmarked version of your revised paper without tracked changes. You should upload this as a separate file labeled 'Manuscript'.

If you would like to make changes to your financial disclosure, please include your updated statement in your cover letter. Guidelines for resubmitting your figure files are available below the reviewer comments at the end of this letter.

If applicable, we recommend that you deposit your laboratory protocols in protocols.io to enhance the reproducibility of your results. Protocols.io assigns your protocol its own identifier (DOI) so that it can be cited independently in the future. For instructions see: https://journals.plos.org/plosone/s/submission-guidelines#loc-laboratory-protocols. Additionally, PLOS ONE offers an option for publishing peer-reviewed Lab Protocol articles, which describe protocols hosted on protocols.io. Read more information on sharing protocols at https://plos.org/protocols?utm_medium=editorial-email&utm_source=authorletters&utm_campaign=protocols.

As the corresponding author, your ORCID iD is verified in the submission system and will appear in the published article. PLOS supports the use of ORCID, and we encourage all coauthors to register for an ORCID iD and use it as well. Please encourage your coauthors to verify their ORCID iD within the submission system before final acceptance, as unverified ORCID iDs will not appear in the published article. *Only*  the individual author can complete the verification step; PLOS staff *cannot*  verify ORCID iDs on behalf of authors.

We look forward to receiving your revised manuscript.

Kind regards,

Pasquale Palumbo

Academic Editor

PLOS One

Journal Requirements:

If the reviewer comments include a recommendation to cite specific previously published works, please review and evaluate these publications to determine whether they are relevant and should be cited. There is no requirement to cite these works unless the editor has indicated otherwise.

Please review your reference list to ensure that it is complete and correct. If you have cited papers that have been retracted, please include the rationale for doing so in the manuscript text, or remove these references and replace them with relevant current references. Any changes to the reference list should be mentioned in the rebuttal letter that accompanies your revised manuscript. If you need to cite a retracted article, indicate the article’s retracted status in the References list and also include a citation and full reference for the retraction notice.

**Additional Editor Comments:**

According to Reviewer 3 comments, only minor, though important issues remain to be addressed, especially the ones focused on limited conceptual novelty and lack of quantitative robustness analysis (it should be carried out at least in a numerical way).

[Note: HTML markup is below. Please do not edit.]

Reviewers' comments:

Reviewer's Responses to Questions

**Comments to the Author**

1. If the authors have adequately addressed your comments raised in a previous round of review and you feel that this manuscript is now acceptable for publication, you may indicate that here to bypass the “Comments to the Author” section, enter your conflict of interest statement in the “Confidential to Editor” section, and submit your "Accept" recommendation.

Reviewer #3: (No Response)

**********

2. Is the manuscript technically sound, and do the data support the conclusions?

The manuscript must describe a technically sound piece of scientific research with data that supports the conclusions. Experiments must have been conducted rigorously, with appropriate controls, replication, and sample sizes. The conclusions must be drawn appropriately based on the data presented.

Reviewer #3: Partly

**********

3. Has the statistical analysis been performed appropriately and rigorously?

Reviewer #3: Yes

**********

4. Have the authors made all data underlying the findings in their manuscript fully available?

The PLOS Data policy requires authors to make all data underlying the findings described in their manuscript fully available without restriction, with rare exception (please refer to the Data Availability Statement in the manuscript PDF file). The data should be provided as part of the manuscript or its supporting information, or deposited to a public repository. For example, in addition to summary statistics, the data points behind means, medians and variance measures should be available. If there are restrictions on publicly sharing data—e.g. participant privacy or use of data from a third party—those must be specified.

Reviewer #3: Yes

**********

5. Is the manuscript presented in an intelligible fashion and written in standard English?

PLOS ONE does not copyedit accepted manuscripts, so the language in submitted articles must be clear, correct, and unambiguous. Any typographical or grammatical errors should be corrected at revision, so please note any specific errors here.

Reviewer #3: Yes

**********

6. Review Comments to the Author

Please use the space provided to explain your answers to the questions above. You may also include additional comments for the author, including concerns about dual publication, research ethics, or publication ethics. (Please upload your review as an attachment if it exceeds 20,000 characters)

Reviewer #3: The revised manuscript demonstrates a clear and substantial effort by the Authors to address the main concerns previously raised. In particular, the revision significantly improves the transparency of the methodological assumptions, the positioning with respect to existing approaches, and the discussion of limitations.

Compliance with major previous comments

Model dependence and identifiability:

The Authors now explicitly acknowledge that the framework relies on a correctly identified PK--PD model and discuss issues such as parameter non-identifiability, correlations, and Jacobian ill-conditioning. The inclusion of a dedicated discussion on inverse-problem aspects and the role of Tikhonov regularization is appropriate and addresses the original concern in a satisfactory way.

Robustness and open-loop nature:

The manuscript now clearly states that the proposed method is an open-loop, nominal optimization approach whose performance depends on model accuracy. The lack of formal robustness guarantees is explicitly recognized, and possible extensions toward adaptive/feedback schemes are outlined. This clarification is adequate and resolves the previous ambiguity.

Structural assumptions and scope:

The Authors have substantially improved the exposition by clearly stating the restrictive assumptions (linear PK, static PD, impulsive dosing, fixed intervals) and framing them as methodological choices for tractability rather than general features. The resulting scope of applicability is now realistically and correctly presented.

Positioning with respect to existing approaches:

The revised introduction provides a more balanced comparison with optimal control, pharmacometric, and exposure-based approaches (including OptiDose). The contribution is now more appropriately framed as a structured formulation exploiting analytical sensitivities and joint optimization of dose and formulation parameters.

Practical constraints and implementation:

The Authors added a detailed discussion of real-world constraints (discrete doses, manufacturability, adherence) and correctly interpret the proposed formulation as a continuous relaxation of a constrained co-design problem. This addresses the previous concern, although at a conceptual level.

Remaining issues.

Despite the improvements, several aspects still limit the scientific strength of the manuscript:

Limited conceptual novelty:

While now better positioned, the work remains largely an integration of known elements (PK-PD modeling, impulsive control, least-squares optimization). The main novelty (joint optimization of dose and liberation rate within a sensitivity-based framework) is valid but incremental. Claims of generality and "unified framework" are still somewhat overstated.

Lack of quantitative robustness analysis:

Although limitations are discussed, no numerical or theoretical analysis is provided to quantify sensitivity of the optimal solution to parameter perturbations. This remains a notable gap for a method intended for decision support.

Local nature of the optimization:

The reliance on local sensitivities and Gauss-Newton is acknowledged, but there is no discussion of convergence, possible nonconvexity, or dependence on initialization. This is particularly relevant given the nonlinear PD mapping.

Validation limited to a single in-silico example:

The illustrative example is clear but does not sufficiently demonstrate generality or robustness across scenarios (e.g., different PK structures or parameter regimes).

Overall recommendation.

The Authors have adequately addressed the main methodological and conceptual concerns raised in the previous review, and the manuscript is now significantly clearer, more balanced, and scientifically better grounded.

The remaining issues are primarily related to scope, strength of claims, and depth of validation, rather than correctness. Provided that claims of novelty and generality are kept appropriately moderate, the manuscript can be considered suitable for publication in a generalist journal such as PLOS ONE.

Further strengthening would be achieved by including even a minimal robustness or sensitivity-to-model-mismatch analysis; however, this is not strictly required for acceptance.

**********

7. PLOS authors have the option to publish the peer review history of their article (what does this mean?). If published, this will include your full peer review and any attached files.

If you choose “no”, your identity will remain anonymous but your review may still be made public.

**Do you want your identity to be public for this peer review?**  For information about this choice, including consent withdrawal, please see our Privacy Policy.

Reviewer #3: No

**********

[NOTE: If reviewer comments were submitted as an attachment file, they will be attached to this email and accessible via the submission site. Please log into your account, locate the manuscript record, and check for the action link "View Attachments". If this link does not appear, there are no attachment files.]

To ensure your figures meet our technical requirements, please review our figure guidelines: https://journals.plos.org/plosone/s/figures

You may also use PLOS’s free figure tool, NAAS, to help you prepare publication quality figures: https://journals.plos.org/plosone/s/figures#loc-tools-for-figure-preparation.

NAAS will assess whether your figures meet our technical requirements by comparing each figure against our figure specifications.

# Author comment: Author response to Decision Letter 3

**DOI:** 10.1371/journal.pone.0354029.r006

17 Jun 2026

See the attached file Review response 3.pdf for the full response to the reviewers

Attachment Submitted filename: Review response 3.pdf

# Peer review: Decision Letter 3

**DOI:** 10.1371/journal.pone.0354029.r007

**Authors:** Pasquale Palumbo

2 Jul 2026

Optimal design of dose and drug pharmacokinetic characteristics to achieve the desired pharmacodynamic profile in repeated drug dosing

PONE-D-26-06346R3

Dear Dr. Dodek,

We’re pleased to inform you that your manuscript has been judged scientifically suitable for publication and will be formally accepted for publication once it meets all outstanding technical requirements.

Within one week, you’ll receive an e-mail detailing the required amendments. When these have been addressed, you’ll receive a formal acceptance letter and your manuscript will be scheduled for publication.

An invoice will be generated when your article is formally accepted. Please note, if your institution has a publishing partnership with PLOS and your article meets the relevant criteria, all or part of your publication costs will be covered. Please make sure your user information is up-to-date by logging into Editorial Manager at Editorial Manager® and clicking the ‘Update My Information' link at the top of the page. For questions related to billing, please contact billing support.

If your institution or institutions have a press office, please notify them about your upcoming paper to help maximize its impact. If they’ll be preparing press materials, please inform our press team as soon as possible -- no later than 48 hours after receiving the formal acceptance. Your manuscript will remain under strict press embargo until 2 pm Eastern Time on the date of publication. For more information, please contact onepress@plos.org.

Kind regards,

Pasquale Palumbo

Academic Editor

PLOS One

Additional Editor Comments (optional):

The Authors addressed all the comments of Reviewer 3: the manuscript is ready for publication

Reviewers' comments:

Reviewer's Responses to Questions

**Comments to the Author**

1. If the authors have adequately addressed your comments raised in a previous round of review and you feel that this manuscript is now acceptable for publication, you may indicate that here to bypass the “Comments to the Author” section, enter your conflict of interest statement in the “Confidential to Editor” section, and submit your "Accept" recommendation.

Reviewer #3: All comments have been addressed

**********

2. Is the manuscript technically sound, and do the data support the conclusions?

The manuscript must describe a technically sound piece of scientific research with data that supports the conclusions. Experiments must have been conducted rigorously, with appropriate controls, replication, and sample sizes. The conclusions must be drawn appropriately based on the data presented.

Reviewer #3: Yes

**********

3. Has the statistical analysis been performed appropriately and rigorously?

Reviewer #3: Yes

**********

4. Have the authors made all data underlying the findings in their manuscript fully available?

The PLOS Data policy requires authors to make all data underlying the findings described in their manuscript fully available without restriction, with rare exception (please refer to the Data Availability Statement in the manuscript PDF file). The data should be provided as part of the manuscript or its supporting information, or deposited to a public repository. For example, in addition to summary statistics, the data points behind means, medians and variance measures should be available. If there are restrictions on publicly sharing data—e.g. participant privacy or use of data from a third party—those must be specified.

Reviewer #3: Yes

**********

5. Is the manuscript presented in an intelligible fashion and written in standard English?

PLOS ONE does not copyedit accepted manuscripts, so the language in submitted articles must be clear, correct, and unambiguous. Any typographical or grammatical errors should be corrected at revision, so please note any specific errors here.

Reviewer #3: Yes

**********

6. Review Comments to the Author

Please use the space provided to explain your answers to the questions above. You may also include additional comments for the author, including concerns about dual publication, research ethics, or publication ethics. (Please upload your review as an attachment if it exceeds 20,000 characters)

Reviewer #3: (No Response)

**********

7. PLOS authors have the option to publish the peer review history of their article (what does this mean?). If published, this will include your full peer review and any attached files.

If you choose “no”, your identity will remain anonymous but your review may still be made public.

**Do you want your identity to be public for this peer review?**  For information about this choice, including consent withdrawal, please see our Privacy Policy.

Reviewer #3: No

**********

# Editor report: Acceptance letter

**DOI:** 10.1371/journal.pone.0354029.r008

**Authors:** Pasquale Palumbo

PONE-D-26-06346R3

PLOS One

Dear Dr. Dodek,

I'm pleased to inform you that your manuscript has been deemed suitable for publication in PLOS One. Congratulations! Your manuscript is now being handed over to our production team.

At this stage, our production department will prepare your paper for publication. This includes ensuring the following:

* All references, tables, and figures are properly cited

* All relevant supporting information is included in the manuscript submission,

* There are no issues that prevent the paper from being properly typeset

You will receive further instructions from the production team, including instructions on how to review your proof when it is ready. Please keep in mind that we are working through a large volume of accepted articles, so please give us a few days to review your paper and let you know the next and final steps.

Lastly, if your institution or institutions have a press office, please let them know about your upcoming paper now to help maximize its impact. If they'll be preparing press materials, please inform our press team within the next 48 hours. Your manuscript will remain under strict press embargo until 2 pm Eastern Time on the date of publication. For more information, please contact onepress@plos.org.

You will receive an invoice from PLOS for your publication fee after your manuscript has reached the completed accept phase. If you receive an email requesting payment before acceptance or for any other service, this may be a phishing scheme. Learn how to identify phishing emails and protect your accounts at https://explore.plos.org/phishing.

If we can help with anything else, please email us at customercare@plos.org.

Thank you for submitting your work to PLOS ONE and supporting open access.

Kind regards,

PLOS ONE Editorial Office Staff

on behalf of

Prof. Pasquale Palumbo

Academic Editor

PLOS One
